# Post-acquisition super resolution for cryo-electron microscopy

**DOI:** 10.1107/S2052252526005348

**Published:** 2026-09-02

**Authors:** Raymond N. Burton Smith, Kazuyoshi Murata

**Affiliations:** a Exploratory Research Center on Life and Living Systems (ExCELLS) National Institutes of Natural Sciences Okazaki Aichi Japan; b National Institute for Physiological Sciences National Institutes of Natural Sciences Okazaki Aichi Japan; c Department of Physiological Sciences, School of Life Science The Graduate University for Advanced Studies (SOKENDAI) Okazaki Aichi Japan; Max Planck Institute of Molecular Physiology, Germany

**Keywords:** cryo-electron microscopy, cryo-EM, image processing, super resolution, Nyquist frequency

## Abstract

Recently, reports have demonstrated achieving resolutions beyond the physical Nyquist limit using super resolution acquisition. Here, we demonstrate exceeding this limitation by pre-processing the raw micrograph movies from counting mode data that have already reached the physical Nyquist reconstruction resolution. To demonstrate functionality, micrograph movies of multiple datasets were pre-processed, showing that it is possible to exceed the physical Nyquist limit via pixel doubling before motion correction. We call this ‘post-acquisition super resolution’, or PASR. While this was originally developed for processing of giant virus datasets, where acquiring at high magnification is not always possible or desirable, it is also shown to work for smaller objects such as adeno-associated virus and apoferritin, both of which are high symmetry, and jack bean urease, with lower symmetry. PASR reduces the magnification required to achieve desired resolutions, which may increase collection efficiency. Further, PASR may be applied on top of acquisition-time super resolution data collection, allowing results to exceed not just the physical sampling limit, but also the super resolution sampling limit. However, to benefit from PASR, data must already reach the Nyquist limit.

## Abstract 2

Post-acquisition super resolution (PASR) is a pre-processing step which can be utilized to exceed the resolution limit of already-acquired data if the limit is reached. It is designed to be as minimally invasive to all general single particle workflows as possible, allowing users to choose which suite they prefer.

## 1. Introduction

In cryo-electron microscopy (cryo-EM), while higher magnification does not always mean higher resolution (Kayama *et al.*, 2021 ▸), the magnification at which data were acquired always causes a limit on the maximum resolution attainable with a dataset. This is derived from the work of H. Nyquist (Nyquist, 1928 ▸) and C. Shannon (Shannon, 1949 ▸) at Bell Laboratories for electronic communications and is applicable to any digital signal processing. These theories, often called the Nyquist theorem or Shannon theorem (or a combination of the two), lead to what in cryo-EM is frequently called the ‘Nyquist limit’. In brief, the Nyquist limit is half the sampling rate of the detector (or the reciprocal of double the pixel size of a digital image) and can be considered the limit on the resolution at a given frequency (or magnification) unless advanced techniques are utilized during acquisition. Often in cryo-EM, however, other factors, such as the microscope environment and sample conditions, come into play before the Nyquist limit is reached. Nonetheless, if this limit is reached, increasing the resolution of cryo-EM 3D reconstructions traditionally required recollection of the data at higher magnification. We recently demonstrated reaching this limit with a single particle analysis (SPA) of a giant virus, Melbournevirus (Burton-Smith *et al.*, 2026 ▸), and the same effect (reaching the Nyquist limit) will have been observed by any cryo-EM users who have downsampled/binned their data to speed up the earlier stages of processing.

**Figure 1:**
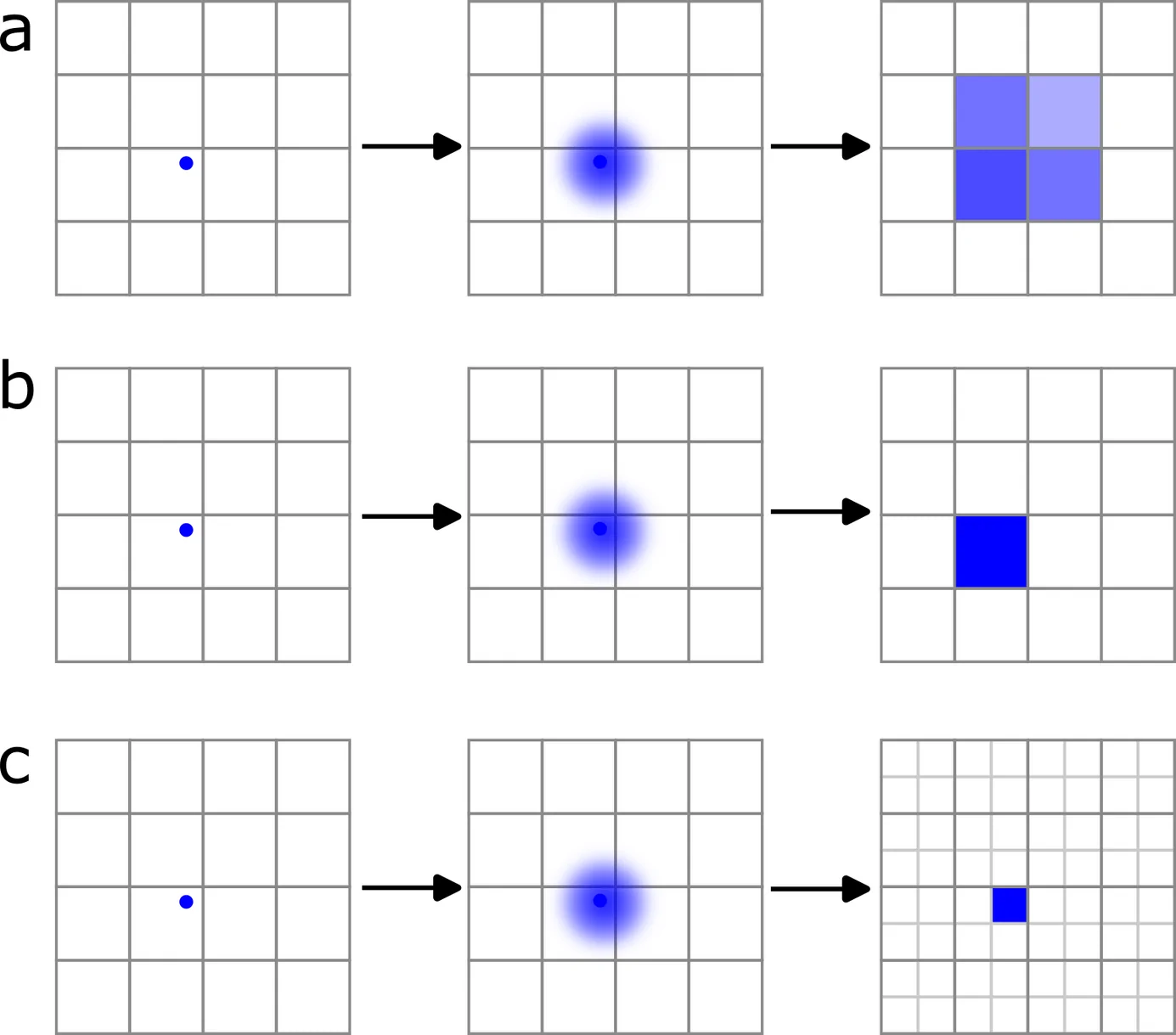
Diagrammatic representation of electron detection on a modern cryo-EM direct detector. (*a*) Integrating (or linear) mode; in this mode, the detector acts in a similar way to old scintillator-coupled CCD devices. When an electron strikes a pixel (left), a so-called ‘electron event’, the charge diffuses through the silicon (centre) such that multiple pixels are read out by the controller with a partial signal from each. This makes the image more blurred. (*b*) Counting mode; in this mode, upon an electron event the controller calculates the pixel with the greatest signal contribution and assigns all the ‘event’ to that single pixel. (*c*) Super resolution mode; this is an enhanced version of counting mode, where the controller estimates where the electron first interacted with a pixel with sub-pixel (quadrant) accuracy via a centroiding algorithm. Adapted with permission from images on Gatan’s website (https://www.gatan.com/improving-dqe-counting-and-super-resolution).

Direct electron detectors (DEDs) are near ubiquitous in cryo-EM facilities now, having gained in popularity due to their contribution towards high-resolution data processing and enhancing acquisition flexibility. DEDs contributed to the ‘resolution revolution’ (Kühlbrandt, 2014 ▸) along with GPU acceleration and other software developments and greater microscope stability. This created an explosion of interest in cryo-EM (Iudin *et al.*, 2023 ▸). The earliest DEDs, such as the FEI (now Thermo Fisher Scientific) Falcon 1 and the Direct Electron DE-12 (Ruskin *et al.*, 2013 ▸), functioned similarly to the scintillator-coupled CCDs which had previously been popular, with a ‘linear’’ or integrating recording mode [Fig. 1 ▸(*a*)], although their physics are fundamentally different, being directly exposed to the electron beam and using a CMOS (complementary metal/oxide semiconductor) technology rather than the earlier digital cameras using a CCD (charge coupled device) sensor more akin to those in modern digital cameras and smartphones. With the release of the Gatan K2 Summit (AMETEK, USA), a ‘counting mode’ became available. In this mode, the controller estimates where the electron originally interacted with the sensor via a thresholding algorithm and defines that pixel as the whole signal [Fig. 1 ▸(*b*)], or finally, super resolution acquisition [Fig. 1 ▸(*c*)], where the localization of the electron signal is estimated by a centroiding algorithm into a quadrant of the pixel. Along with dose fractionation, allowing the acquisition of ‘movies’ of cryo-EM data to compensate for stage instability and particle movement (Brilot *et al.*, 2012 ▸; Campbell *et al.*, 2015 ▸), direct detectors contributed towards great advances in achievable resolution.

Super resolution is a technique now widely used across a range of disciplines for acquisition of data, although implementations vary (Betzig & Trautman, 1992 ▸; Chiu *et al.*, 2015 ▸; Guerra, 1995 ▸; Poot *et al.*, 2013 ▸). In cryo-EM, the first commercially available camera/detector with super resolution capability was the Gatan K2 Summit, and super resolution is now available in direct detectors from Gatan (Pleasanton, California, USA), Thermo Fisher Scientific (Waltham, Massachussetts, USA) and Direct Electron (San Diego, California, USA), but it is not ubiquitous. In cryo-EM, the technique involves identification of an electron event (where an electron strikes the detector in such a way as to be recorded) in a pixel where its exact location is algorithmically estimated [Fig. 1 ▸(*c*)], but unless using the electron event representation (EER) format, it must be explicitly selected at acquisition time for data to be output in this manner. The EER file format developed for the Falcon 4 DED allows three levels of flexibility for processing a single recorded output, corresponding to 4K (equivalent to counting mode), 8K (equivalent to super resolution) and 16K [which we refer to as ‘double super resolution’ (DSR) as it has not been formally named] micrograph output (Guo *et al.*, 2020 ▸). A great deal of use has been made of super resolution acquisition since it became available to cryo-EM facilities, and many super resolution datasets are available publicly in the EMPIAR database (Iudin *et al.*, 2023 ▸; Iudin *et al.*, 2016 ▸). Reconstructions ‘past Nyquist’ have been demonstrated using super resolution data (Feathers *et al.*, 2021 ▸), although these data were still explicitly recorded at acquisition time with super resolution mode. There has also been interest in neural network optimization similar in effect to our work presented here (Huang *et al.*, 2023 ▸).

However, giant structures (particles >150 nm) present a significant challenge to cryo-EM. Resolution limitations caused by defocus gradients can be compensated for by using Ewald sphere correction (DeRosier, 2000 ▸; Wolf *et al.*, 2006 ▸; Russo & Henderson, 2018 ▸; Zivanov *et al.*, 2018 ▸) or ‘block-based’ reconstruction methods (Zhu *et al.*, 2018 ▸) or a combination of the two. Ewald sphere correction has been demonstrated to improve resolution of objects as small as apoferritin (Nakane *et al.*, 2020 ▸; Yip *et al.*, 2020 ▸). Ultimately, for giant structures the object of interest is extremely large for high resolution single particle reconstruction (SPR) cryo-EM due to difficulties in both data acquisition and processing. As a result, data acquisition for giant structures is a careful balancing act between a high enough magnification to achieve (near) atomic resolution and low enough magnification to avoid needing to collect tens or hundreds of thousands of micrographs to acquire a similar number of usable particles. One previous work on giant viruses demonstrated the need to acquire almost one micrograph per selected particle (Wang *et al.*, 2019 ▸).

We previously reported (Burton-Smith *et al.*, 2026 ▸) a reconstruction of Melbournevirus, a giant virus with a maximum dimension of ∼250 nm, to a resolution of 4.9 Å for the whole viral particle and to 4.42 Å (the maximum attainable resolution from that dataset) for block-based reconstructions of the capsid at the two-, three- and fivefold axes. Subsequently, however, we were able to apply Bayesian polishing (Zivanov *et al.*, 2019 ▸) to the whole virus to reach the Nyquist limit for the whole virus reconstruction (EMD-37169).

As reported, when using the block-based reconstruction method for Melbournevirus, after localized defocus refinement the solvent-corrected gold-standard FSC curves reached the Nyquist limit above the 0.143 metric, indicating that if data had been collected at higher magnification, higher resolution would have been achieved (Burton-Smith *et al.*, 2026 ▸). This caused us to ponder: would pre-processing of the counting mode data to simulate ‘super resolution’ data permit us to break this resolution limit as super resolution at acquisition time has done? Would it cause artefacts in the resulting reconstruction? In short; yes, it does, and no, it does not, respectively.

**Figure 2:**
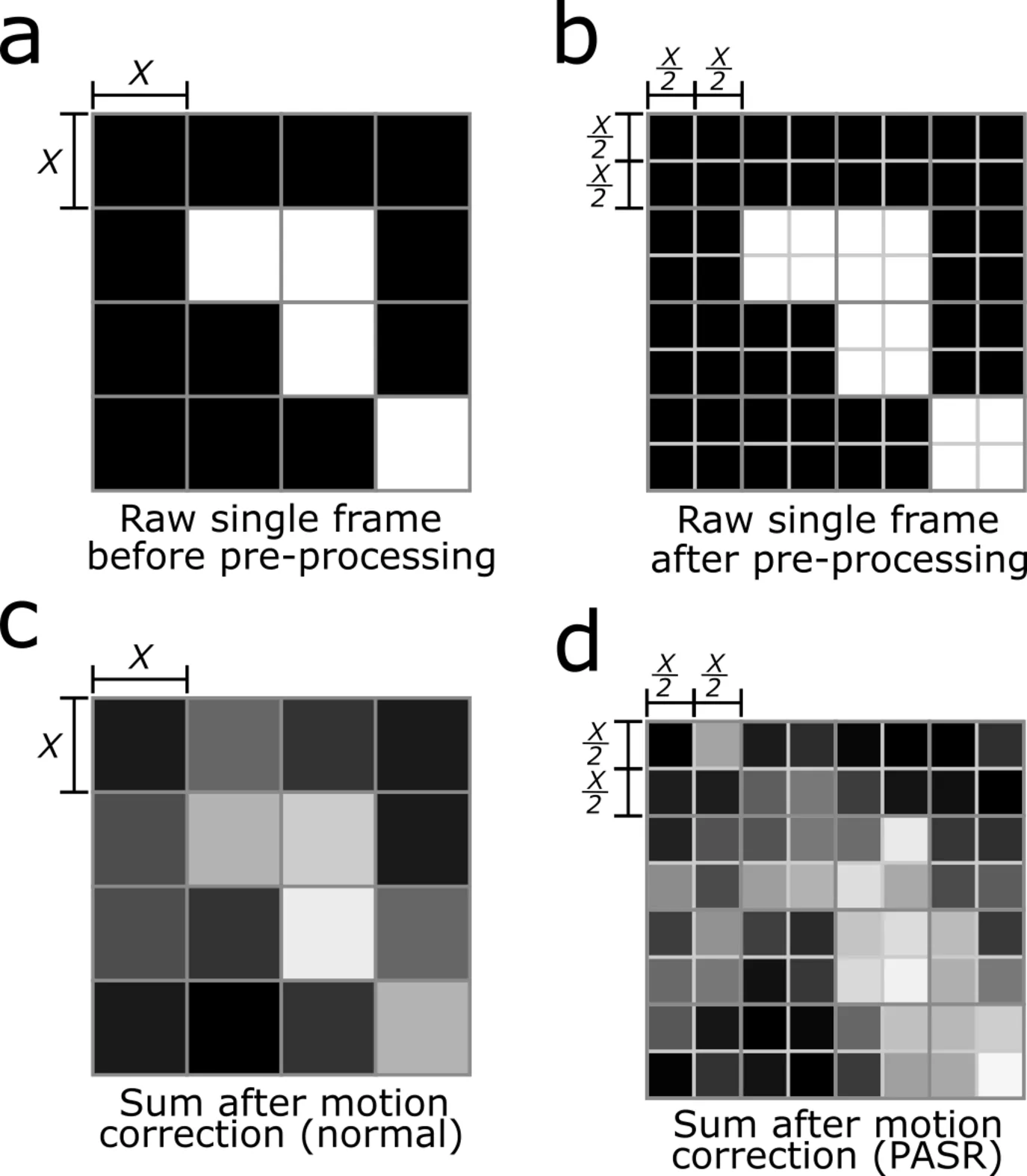
Diagrammatic representation of the post-acquisition super resolution (PASR) method. (*a*) A normal micrograph frame where each pixel has the effective sampling rate of *x* × *x* Å. (*b*) The raw frames are pre-processed using a script in GNU Octave or Python to make each pixel a 2 × 2 grid of ½*x* × ½*x* Å across all pixels and all frames of the micrograph. After motion correction, each full pixel from the original micrograph movie is unique (*c*), while with the PASR pre-processed micrograph movies, each sub-pixel in the resulting micrograph is unique (*d*).

Here, we demonstrate the potential of a ‘post-acquisition’ super-resolution-like pre-processing step, where each pixel of each frame of a counting mode micrograph [Fig. 2 ▸(*a*)], which would normally be motion-corrected as discrete pixels [Fig. 2 ▸(*c*)], is split into four identical sub-pixels before motion correction [Fig. 2 ▸(*b*)]. After motion correction is performed, each pixel in the resulting sum is unique [Fig. 2 ▸(*d*)]. This is the ‘dithering’ method, first used in astrophotography (Adorf & Hook, 1995 ▸; Hook & Fruchter, 2000 ▸), applied to cryo-EM. As such, we require at least a small amount of motion between frames, but of course too much movement becomes detrimental. PASR does not require complex multi-pass neural network processing to achieve resolution improvements like cryo-ZSSR (Huang *et al.*, 2023 ▸), focusing on minimal changes to the dominant cryo-EM processing pipelines to avoid complexity for the end user.

As this effectively simulates super resolution without the sub-pixel localization, but rather dithering during frame alignment, we call this ‘post-acquisition super resolution’, or PASR. We demonstrate this for two publicly available datasets of higher and lower symmetry: apoferritin, an octahedral symmetry complex which is now a favourite in the cryo-EM field for benchmarking and testing (Kayama *et al.*, 2021 ▸; Nakane *et al.*, 2020 ▸; Danev *et al.*, 2019 ▸; Hamaguchi *et al.*, 2019 ▸; Noble *et al.*, 2018 ▸) and which currently holds the world record for attained cryo-EM resolution (Yip *et al.*, 2020 ▸), and jack bean urease (Feathers *et al.*, 2021 ▸), as it is lower symmetry and a public dataset was available on EMPIAR (Iudin *et al.*, 2016 ▸; Iudin *et al.*, 2023 ▸). These allowed us to compare the results of super resolution acquisition and our PASR technique and permitted testing for 200 kV data. Three other datasets of higher symmetry were also tested; first, an adeno-associated virus (AAV) dataset generously provided by a collaborator from a different study, which allowed us to test the application of PASR to correlative double sampling (CDS) acquisition. Second, a subset of an apoferritin dataset acquired at nearly 2 Å pixel^−1^, and third, by reprocessing our previously reported Melbournevirus dataset (Burton-Smith *et al.*, 2026 ▸) which inspired this work. We further carry out a brief demonstration of the PASR method on already-motion-corrected micrographs, albeit with inferior results compared with application to ‘movies’ prior to motion correction, which is as we expected. Finally, we demonstrate that PASR may be applied to acquisition-time super resolution (ASR) data, to exceed the super resolution Nyquist limit as well.

## 2. Results

### 2.1. Motion correction and CTF estimation

We tested motion correction of sub-pixelized movie frames with both *MotionCor2* (Zheng *et al.*, 2017 ▸) and the *RELION* implementation (Zivanov *et al.*, 2018 ▸), both of which proceeded with no errors. A high-zoom visual examination of motion-corrected micrographs using *Fiji*/*ImageJ2* (Schindelin *et al.*, 2012 ▸) revealed no abnormalities [such as pixels with values of infinity or ‘not a number’ (NaN)], with individual pixels differing from neighbouring pixels (data not shown). We tested datasets from a range of detectors and accelerating voltages to see whether acquisition hardware affected the effectiveness of PASR (Table 1 ▸).

**Figure 3:**
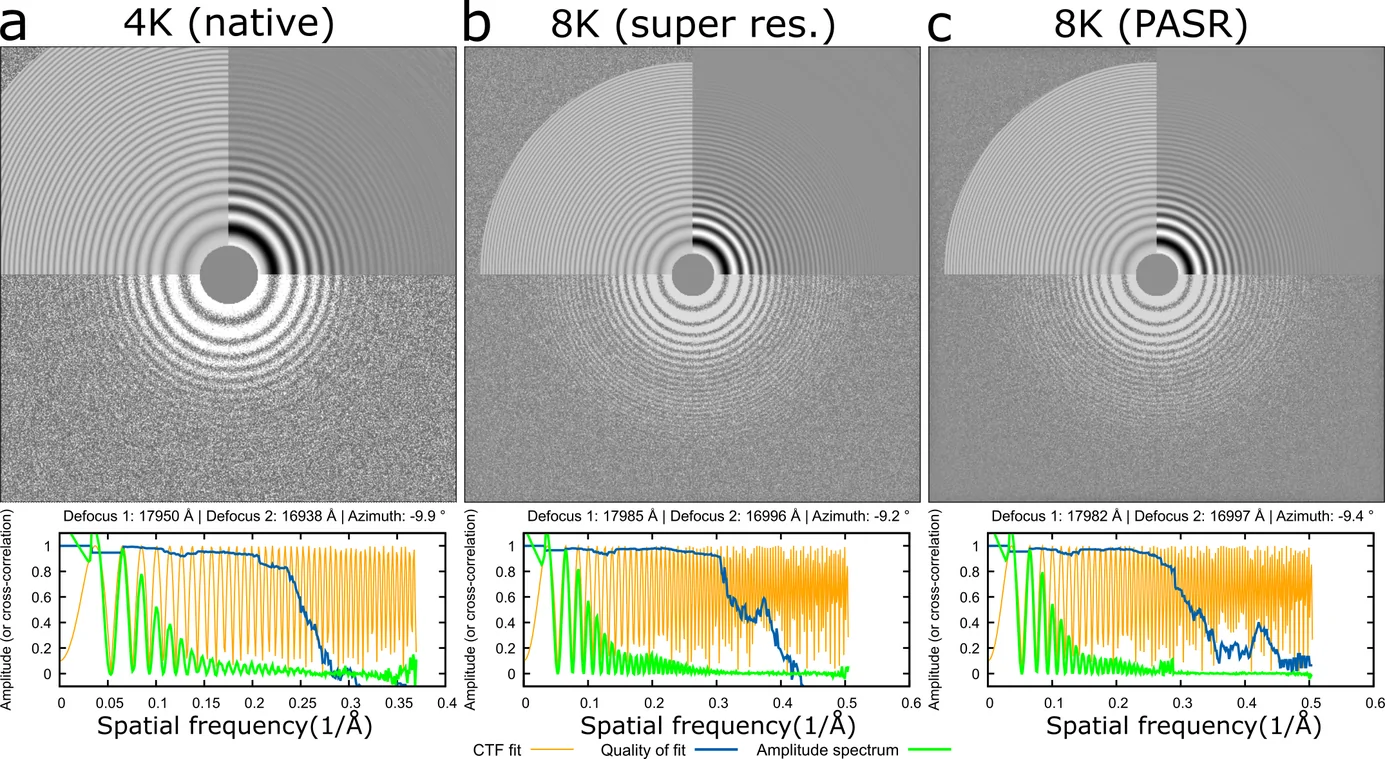
Comparison of contrast transfer function (CTF) estimation for 4K (*a*: native), 8K (*b*: super resolution) and 8K (*c*: PASR-preprocessed 4K micrographs) from the same micrograph. The original micrograph was collected using the EER file format and converted to either 4K TIFF or 8K TIFF using the *RELION* function* relion_convert_to_tiff*. *CTFFIND* was used to calculate parameters for each with the following settings: FFT box size: 512 pixels, minimum resolution: 30 Å, maximum resolution: 3 Å, minimum defocus: 1000 Å, maximum defocus: 50 000 Å, defocus step size: 100 Å. For each condition, the power spectrum is displayed, showing clear Thon rings, along with the *CTFFIND*-derived rotational average and simulated fit (top). Underneath, 1D diagnostic plots from *CTFFIND* are shown, demonstrating highly similar calculated defocus and astigmatism values. The sampling frequency of the 4K micrograph is 1.912 Å pixel^−1^, while for the super resolution and PASR micrographs the sampling frequency is 0.956 Å pixel^−1^.

If we were to encounter issues, CTF (contrast transfer function) estimation was expected to be the first point of the processing pipeline to exhibit it. Fig. 3 ▸ shows power spectra from the same original apoferritin micrograph from dataset 1 (hereafter, Apo64K) when processed at 4K (native sampling) (Supplementary Fig. 1), 8K super resolution (Supplementary Fig. 2) and 8K PASR (Supplementary Fig. 3) settings (Supplementary Table 1). Underneath each power spectrum in Fig. 3 ▸ is the 1D diagnostic plot from *CTFFIND* 4.1.14 (Rohou & Grigorieff, 2015 ▸), showing that the estimated defocus values and azimuth are comparable between the three. The 4K micrograph power spectrum is ‘zoomed in’ as the sampling limit is 3.82 Å rather than 1.91 Å as is the case for super resolution and PASR samplings. Occasionally, a few micrographs ‘fail’ CTF estimation – where the simulated CTF fits the Thon rings poorly – with PASR-processed images but not in the original data (see Supplementary Tables 1–6). This may be caused by the decrease in contrast [Supplementary Figs. 1 ▸(*a*)–3(*a*)] for super resolution and PASR micrographs relative to micrographs acquired at physical Nyquist sampling. Micrographs that ‘pass’ CTF estimation demonstrate very similar defocus, astigmatism and maximum resolution estimates (Fig. 3 ▸, Supplementary Tables 1–6). Only *CTFFIND4* (Rohou & Grigorieff, 2015 ▸) was used for *RELION*. On the same micrograph, the patch CTF estimation of *Cryo­SPARC* (Punjani *et al.*, 2017 ▸) reports similar values to *CTFFIND* for the 8K super resolution and 8K PASR data [Supplementary Figs. 4(*c*) and 5(*c*)].

### 2.2. Particle picking and 2D classification

Laplacian-of-Gaussian (LoG) (Zivanov *et al.*, 2018 ▸) and template-based (Scheres, 2015 ▸) autopicking methods were unaffected (Supplementary Figs. 6–13) by PASR processing, resulting in similar numbers of particles when used for a dataset (Supplementary Tables 1–6) with the same parameters.

**Figure 4:**
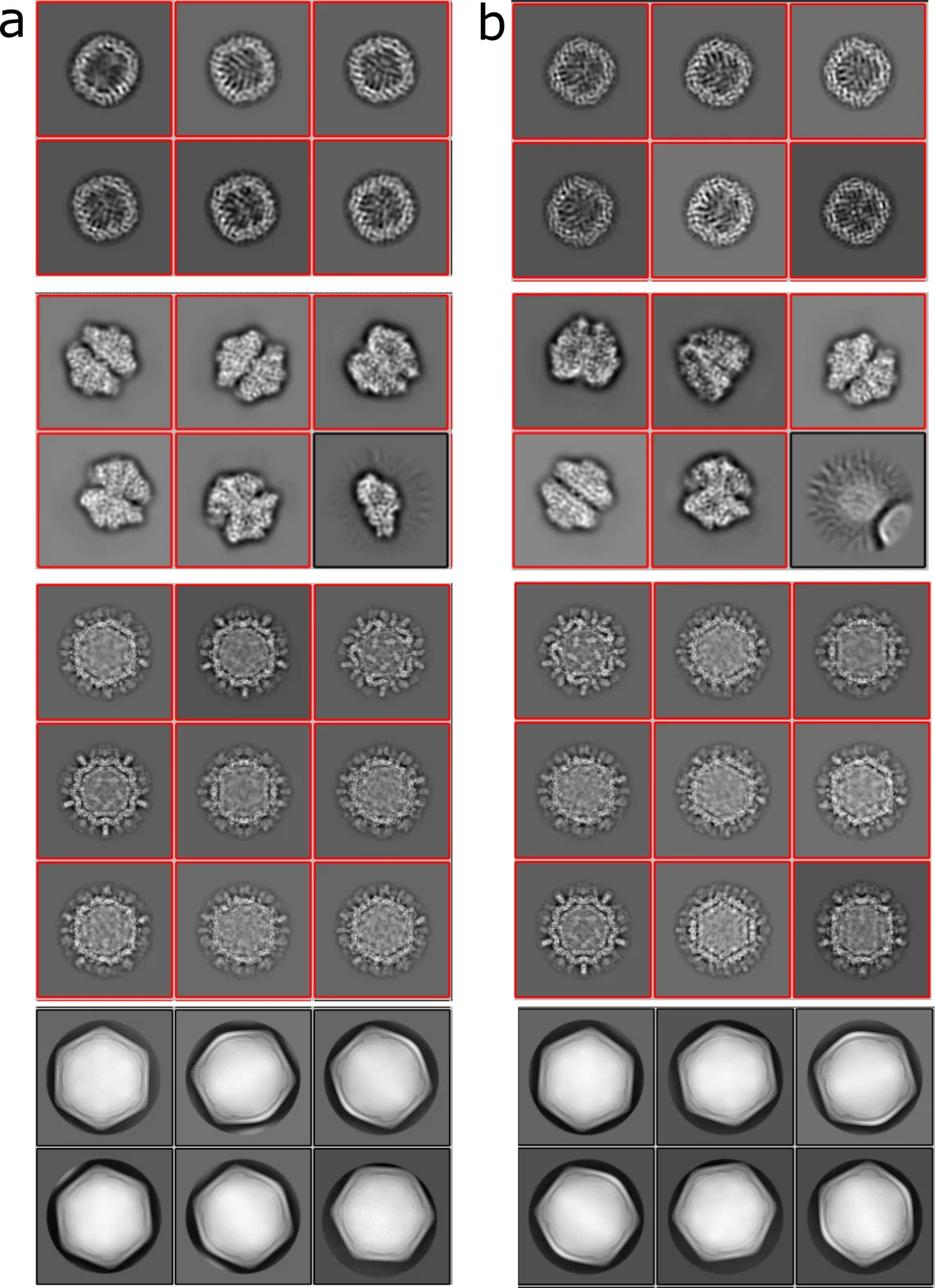
Example 2D classes from different datasets showing (*a*) classes from original data and (*b*) classes from PASR data. Classes from apoferritin, jack bean urease, nodavirus and Melbournevirus processing are shown from top to bottom. Particles from different datasets are not on the same scale.

2D classification appears unaffected by PASR processing. Fig. 4 ▸ shows some example classes from different datasets and PASR is visually indistinguishable from native 2D classes. This is as expected, as early stages of classification are usually carried out on downsampled data. Interestingly, the PASR data for apoferritin (EMPIAR-10216) (Supplementary Table 2) classified into far fewer classes (Supplementary Fig. 14) than the binned or original data (Supplementary Figs. 15 and 16) when using the same settings, but the number of particles contained within these good classes was approximately the same (Supplementary Table 2). Conversely, for the Apo64K dataset (Supplementary Table 1, Supplementary Fig. 1), the 4K sampling data had fewer clear classes and contained fewer particles in total than the 8K sampling (Supplementary Fig. 2) or PASR (Supplementary Fig. 3) datasets.

### 2.3. Initial model generation

Initial model generation remains one of the more challenging aspects of cryo-EM single particle analysis and much effort has been put into improving it (Zivanov *et al.*, 2018 ▸; Punjani *et al.*, 2017 ▸; Gomez-Blanco *et al.*, 2019 ▸; Grant *et al.*, 2018 ▸; Kimanius *et al.*, 2021 ▸; Reboul *et al.*, 2018 ▸). We tested initial model generation throughout the datasets. For every case except one, the *RELION* initial model algorithm was successful in generating an acceptable initial model which resembled the 2D classes used. For the urease dataset (Supplementary Table 3), the PASR-processed dataset failed to generate a good initial model in *RELION*. To generate a good initial model from the PASR dataset we exported the particle stack to *cisTEM* (Grant *et al.*, 2018 ▸) to successfully generate an initial model. As we have previously had success with *cisTEM* initial model generation where *RELION* initial model generation fails, we do not consider this an impediment to PASR. In a subsequent processing run, initial model generation was successful the first time. Initial model generation with the Variable-metric Gradient Descent with Adaptive Moments (VDAM) algorithm of *RELION* 4 (Kimanius *et al.*, 2021 ▸) was successful with the 8K PASR apoferritin dataset but needed to be carried out a second time on the 8K super resolution dataset before a good initial model was generated. Initial model generation in *Cryo­SPARC* was successful on the first attempt for the PASR data but the second attempt for the super resolution data with default parameters, whether symmetry is imposed or not during initial model generation. With parameters better optimized for apoferritin (20 Å starting resolution, 8 Å final resolution, in the same manner as in *cisTEM*) initial model generation was successful first time for both.

### 2.4. 3D classification and imaging parameter refinements

3D classification (when used, *e.g.*, Melbournevirus) and refinement displayed no anomalies in processing. Defocus and astigmatism refinement were unaffected, with refined values within expected tolerances (*i.e.* variance between normal, super resolution and PASR defocus estimates was small) given those obtained with the original datasets. Magnification anisotropy and beam tilt estimation were also within tolerance. Bayesian polishing likewise showed no adverse effects, although, like native super resolution data, the PASR datasets took longer to analyse in the first step due to the increased size of the micrographs.

### 2.5. Final map evaluation

**Figure 5:**
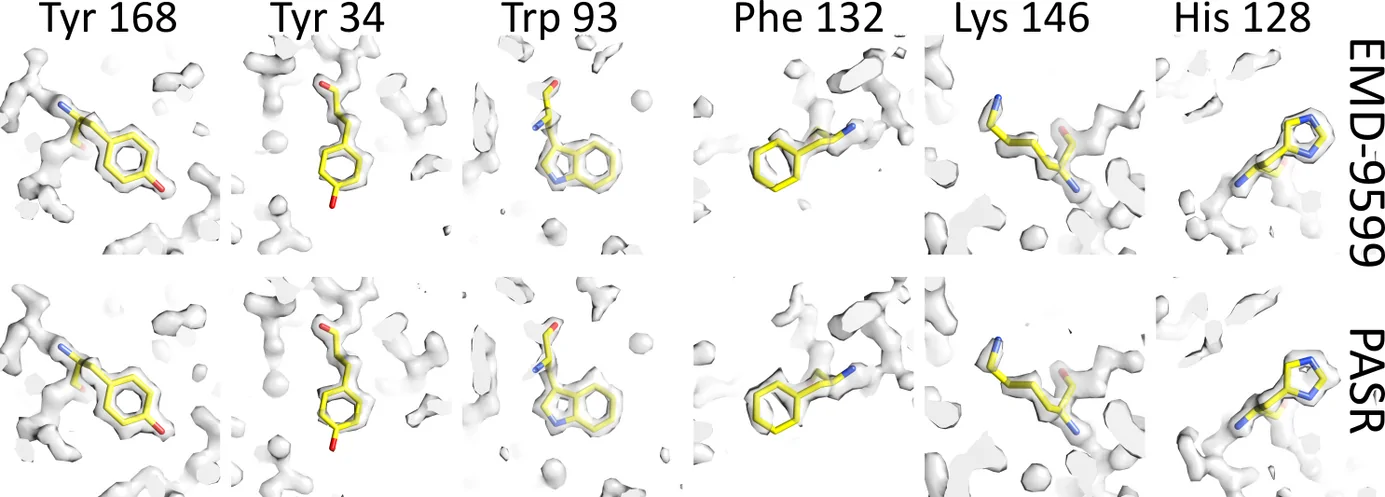
Comparing side chains of PASR-processed apoferritin data achieving <2 Å resolution and directly comparing with EMD-9599 at 1.63 Å resolution. When reprocessing EMPIAR-10216, 1.71 Å resolution was achieved while PASR achieved 1.78 Å resolution. Six example side chains of varying quality are shown; In EMD-9599, the five-member-ring nature of His128 is clear, while with PASR the hole is less well defined. Lys146 and Phe132 are comparable. Trp93 shows clear holes for both the five- and six-member rings for both the original data and PASR. For both the original data and PASR processing, the density of tyrosine is dependent on location; Tyr34 is weaker, while Tyr168 is clear and strong with a clear hole in the six-membered ring. Density maps are displayed at 5σ. Even at <2 Å resolution, PASR is competitive in terms of clarity with data collected at the higher magnification in counting mode.

PASR data suffer a marginal loss in reported final resolution compared with the original data for apoferritin (EMPIAR-10216) (Supplementary Table 2) and urease (EMPIAR-10549) (Supplementary Table 3), although this may be in part because of the need to first downsample the data from the original data uploaded to EMPIAR. However, the side chains look comparable (Figs. 5 ▸ and 6 ▸). *Q*-scores and map-to-model FSCs are also extremely close between the original and PASR data; *e.g.* 0.86 for the reprocessing of EMPIAR-10216 and 0.85 for the PASR processing (Supplementary Tables 1–3 and 5–7). As *Q*-scores should, the value scales lower with a strong resolution dependency. While PASR alignments report slightly worse resolution (0.2 Å decrease by gold-standard FSC) than the 8K sampled micrographs for our apoferritin dataset, once again, the side chains look comparable (Fig. 7 ▸). For both Melbournevirus (Fig. 8 ▸) (Supplementary Table 4) and AAV (Supplementary Table 5), PASR data are a visible improvement over the original data. Local resolution estimation of the PASR Melbournevirus blocks exceeds the Nyquist limit of the original data and is in line with local resolution estimates to be expected given the gold-standard FSC reported [Fig. 8 ▸(*a*)]. Model building and refinement for the PASR-processed Melbournevirus capsid improved the *Q*-score for the major capsid protein (MCP) relative to the 4.4 Å map (Supplementary Table 4), although, as most proteins are ‘hypothetical’, work is ongoing to identify and build a full capsid model. The PASR maps have been used to build models into locations based on interactomics data (Mühlberg *et al.*, 2025 ▸) and into another related virus, Jyvaskylavirus (Almeida *et al.*, 2025 ▸). Other PASR-processed datasets show the same when compared with Nyquist limited data (Supplementary Figs. 1–16).

PASR can function on pre-motion-corrected micrographs if the data reach the Nyquist limit if applied to either the raw micrographs or a particle stack. However, the improvement is markedly less (Supplementary Fig. 17) than for either native data or on PASR applied prior to motion correction (Supplementary Figs. 1–5). We used nodavirus from EMPIAR-10203 (Ho *et al.*, 2018 ▸) (Supplementary Table 6) to test further and downsampled the micrographs in Fourier space by a factor of two. This made the effective sampling frequency 2.12 Å pixel^−1^. A reconstruction with these binned data reached the sampling limit (Supplementary Fig. 18). Applying PASR to these downsampled micrographs and then reprocessing permitted recovery of information up to a gold-standard FSC of 3.21 Å (Supplementary Fig. 19). These data were originally published at 3.28 Å (Ho *et al.*, 2018 ▸). *Q*-scores and map-to-model FSCs are acceptable for the reported values (Supplementary Table 6). We have previously reprocessed these data and achieved 2.9 Å (Supplementary Fig. 20).

### 2.6. Application tests for PASR

Rather than reprocess everything again in *Cryo­SPARC* (Punjani *et al.*, 2017 ▸), we tested the Apo64K dataset (Supplementary Table 1, Supplementary Figs. 4 and 5). This subset was originally collected as part of a much larger dataset (Burton-Smith *et al.*, to be published) on the Titan Krios G4 (Thermo Fisher Scientific) at the Exploratory Research Center on Life and Living Systems with a Falcon 4 camera. As the Falcon 4 EER format allows sampling at 4K (native), 8K (super resolution) or 16K (DSR), we pre-processed TIFF converted micrographs at 4K with PASR and compared them with 8K sampling. The *Cryo­SPARC* pipeline shows no anomalous results with PASR pre-processed data (Supplementary Fig. 5). All reconstructions resulted in maps which were visually appropriate for the reported resolution with no artefacts visible in the maps, half maps or diagnostic metric output. *Q*-scores are again in line with values that should be expected for maps of the reported resolution (0.81 for the native super resolution map and 0.79 for the PASR map), as are map-to-model FSCs.

We zero-padded the half maps from one of the Melbourne­virus reconstructions, rescaled the mask appropriately and carried out post processing (Supplementary Fig. 21). As is expected with zero padding, the FSC of the unmasked half maps falls to zero at the original sampling limit and flatlines [green curve, Supplementary Fig. 21(*a*)]. However, the phase-randomized FSC had fallen to zero where randomization begins but increases again once past the original sampling limit [red curve, Supplementary Fig. 21(*a*)]. When *RELION* was left to estimate the *B* factor, rather than manually imposing one, the estimated *B* factor exceeded −1500. Furthermore, local resolution estimation reports exactly half the local resolution estimation from the non-zero padded data [Supplementary Fig. 21(*b*)]. PASR-processed data do not demonstrate these artefacts. Finally, the map looks like one that has been rescaled (resampled) after completion [*e.g.* in *UCSF Chimera* (Pettersen *et al.*, 2004 ▸; Goddard *et al.*, 2007 ▸) or with *relion_image_handler*] [Supplementary Fig. 21(*c*)].

A final test was carried out on data collected on a CRYOARM 300 (JEOL) equipped with a Gatan K3 direct detector. Data were acquired with super resolution acquisition (ASR) and the micrographs pre-processed with the PASR script. The dataset was processed with *RELION* (3.1). From a physical Nyquist limit of 1.9708 Å pixel^−1^ (after calibration) – and super resolution sampling equivalent to 0.9854 Å pixel^−1^ – the PASR micrographs were equivalent to 0.4927 Å pixel^−1^. Processing proceeded normally, but it should be noted that the hardware demands of Bayesian polishing were extremely high. Supplementary Fig. 22 contains a representative micrograph, showing that almost the entire area of a standard QuantiFoil R1.2/1.3 grid hole can be acquired, as well as 2D classes, the FSC curve and some representative side chains. Nevertheless, the reconstruction achieved 1.67 Å (236% higher than the physical sampling limit and 118% higher than the super resolution sampling limit). The EMDB map and PDB model for this result have been deposited as EMD-65822 and 9wal, respectively. The *Q*-score for the model fitted to the map is 0.91 irrespective of whether water molecules are built into the map. The map-to-model FSC is 1.68 Å.

During review, the question of potential over-fitting and accurate sub-pixel positioning was raised. To this end, we created a short script (also available on GitHub) which ‘flips’ sub-pixels either in columns, rows or both on a per (physical) pixel basis. Testing the resulting sub-pixel flips demonstrates that now-standard motion correction, when applied to PASR pre-processed movies, yields meaningful sub-pixel alignment, permitting recovery of higher-resolution information than the original data would support. Both column- and row-based sub-pixel flipping reduces reported resolution and map quality (and reported quality metrics such as map-to-model FSC and *Q*-score), while the combined flip exacerbates this further (Supplementary Figs. 23–27 and Table 9). This is also linked to pixel size – when testing a small counting mode dataset (where the pixel size was ∼30% of the reported final resolution), we noted an ∼10% loss in resolution for individual column or row flipping, and ∼25% when both were applied. Applying pixel flipping to data where the pixel size was larger, the effect was more dramatic – completely ruining what had been a good-quality map and FSC, turning both map and FSC pathological (Supplementary Fig. 23). Similarly, *Q*-scores were severely impacted. For this same data when PASR processed, the reported resolution loss was significant, but did not turn the map or FSC pathological (Supplementary Fig. 24). For higher-magnification data, positions of smaller densities – which could be attributed to water or other small molecules (or noise) – were impacted when both row and column pixels were flipped (Supplementary Fig. 25), although this might be attributed to the loss of resolution, and filtering imposed in post-processing. Whether carrying out simple reconstructions or a full re-refinement of the dataset, the loss in reported resolution was similar (Supplementary Table 9). Native super resolution and PASR data behaved the same when tested with pixel flipping (Supplementary Figs. 26 and 27).

## 3. Discussion

Here we demonstrate a super resolution pre-processing step for cryo-EM data that are conventionally limited by Nyquist. All data subjected to PASR pre-processing behaved as one would normally expect when using the popular *RELION* suite (Fernandez-Leiro & Scheres, 2017 ▸; Scheres, 2012 ▸). The dataset tested in *Cryo­SPARC* (Punjani *et al.*, 2017 ▸) also behaved normally. The dataset requiring initial model generation in *cisTEM* (Grant *et al.*, 2018 ▸) also behaved normally, although a full processing pipeline was not carried out.

While in some datasets fewer micrographs passed CTF estimation when PASR pre-processed, most micrographs were still perfectly acceptable, and defocus, astigmatism and maximum resolution estimated by *CTFFIND* (Rohou & Grigorieff, 2015 ▸) for original and PASR pre-processed micrographs showed highly similar defocus, astigmatism and fit estimates; often <10 Å deviation in defocus (Fig. 3 ▸). Perhaps the micrographs that failed CTF estimation with PASR pre-processing did so due to decreased contrast, as both super resolution and PASR data demonstrate lower contrast than ‘native’ data, and downsampling native data also decreases the number of micrographs that pass CTF estimation. Or perhaps they were close to failing and may have done so with a different version of *CTFFIND*, as we have noted that some releases of *CTFFIND* appear more forgiving than others for difficult micrographs. A weak crystalline ice signal in the CTF is over-emphasized in some PASR micrographs [Fig. 3 ▸(*c*)], which may also play a role. CTF estimation worked when using both the motion-corrected micrographs for CTF estimation (Supplementary Figs. 6–16) and the independent power spectrum (Supplementary Figs. 1–3) which can be optionally generated by *RELION*. Patch CTF in *Cryo­SPARC* (Punjani *et al.*, 2017 ▸) completed without error (Supplementary Figs. 4 and 5).

Previously, the solution to reaching the Nyquist limit would be to collect more data at a higher magnification. This obviously requires further microscope time, and more storage space. However, sometimes this is not possible, or is even undesirable due to the type of sample. The resolution limit in the Melbournevirus dataset (Burton-Smith *et al.*, 2026 ▸) was the *raison d’être* for this investigation. Acquiring giant virus datasets at higher magnifications can lead to tens or hundreds of thousands of micrographs for the same number of, or fewer, usable particles (Wang *et al.*, 2019 ▸), possibly without achieving a resolution commensurate with that magnification due to other factors that influence cryo-EM SPA and particularly giant virus reconstructions. Size is not the only challenge in processing giant viruses. For example, they can demonstrate pseudosymmetry – the block-based reconstruction technique was applied to PBCV-1 with icosahedral symmetry initially (Fang *et al.*, 2019 ▸), however it is not icosahedral in form (Shao *et al.*, 2022 ▸). Likewise, Mimivirus was initially analysed as icosahedral in form (Xiao *et al.*, 2005 ▸), but has a ‘stargate’ which means it demonstrates only fivefold symmetry (Xiao *et al.*, 2009 ▸) and potentially asymmetry. Capsids can also be frustratingly heterogeneous (Watanabe *et al.*, 2022 ▸), demonstrating flexibility which limits achievable resolution even at lower magnifications. For working with giant viruses, flexibility in both acquisition and processing are critical. PASR processing of the Melbournevirus data yielded maps from block-based reconstruction (Zhu *et al.*, 2018 ▸) which already exceeded the original sampling limit. When Bayesian polishing was applied (using the default σ parameters) the final gold-standard FSC reported for each block was 3.5 Å for the twofold block (Supplementary Fig. 6), 3.5 Å for the threefold block (Supplementary Fig. 7) and 3.4 Å for the fivefold block (Fig. 8 ▸, Supplementary Fig. 8), with local resolution extending to ∼3.2 Å for all three blocks (Supplementary Figs. 6–8). This is a dramatic improvement.

Other datasets (Table 1 ▸) were used to demonstrate that PASR is applicable to several complexes acquired with some of the more popular detectors [Falcon 3, Falcon 4(i), K2 Summit, K3] and microscopes (200 and 300 kV). The downsampling (Supplementary Fig. 15) and PASR (Fig. 5 ▸, Supplementary Fig. 14) reprocessing of an earlier, public apoferritin dataset (Fig. 5 ▸, Supplementary Fig. 16) was used so that we could directly compare with ‘native’ data. The downsampling processing of individual frames was necessary so that PASR could be applied to a ‘Nyquist limited’ micrograph movie dataset, as (at the time of writing), except for EMPIAR-10549 (Feathers *et al.*, 2021 ▸), there are no public datasets that exceed the physical Nyquist limit.

Similarly, the downsampling (Fig. 7 ▸, Supplementary Fig. 1) of the super resolution (Fig. 7 ▸, Supplementary Fig. 2) dataset before PASR (Fig. 7 ▸, Supplementary Fig. 3) pre-processing of the downsampled data was used to permit a comparison with ‘native’ super resolution, *i.e.* acquisition-time super resolution, data. ASR yields slightly higher resolutions than PASR (2.1 Å versus 2.3 Å) because with ASR the sub-pixels are unique, while with PASR they are identical. The same is true for per-particle alignment in Bayesian polishing.

**Figure 6:**
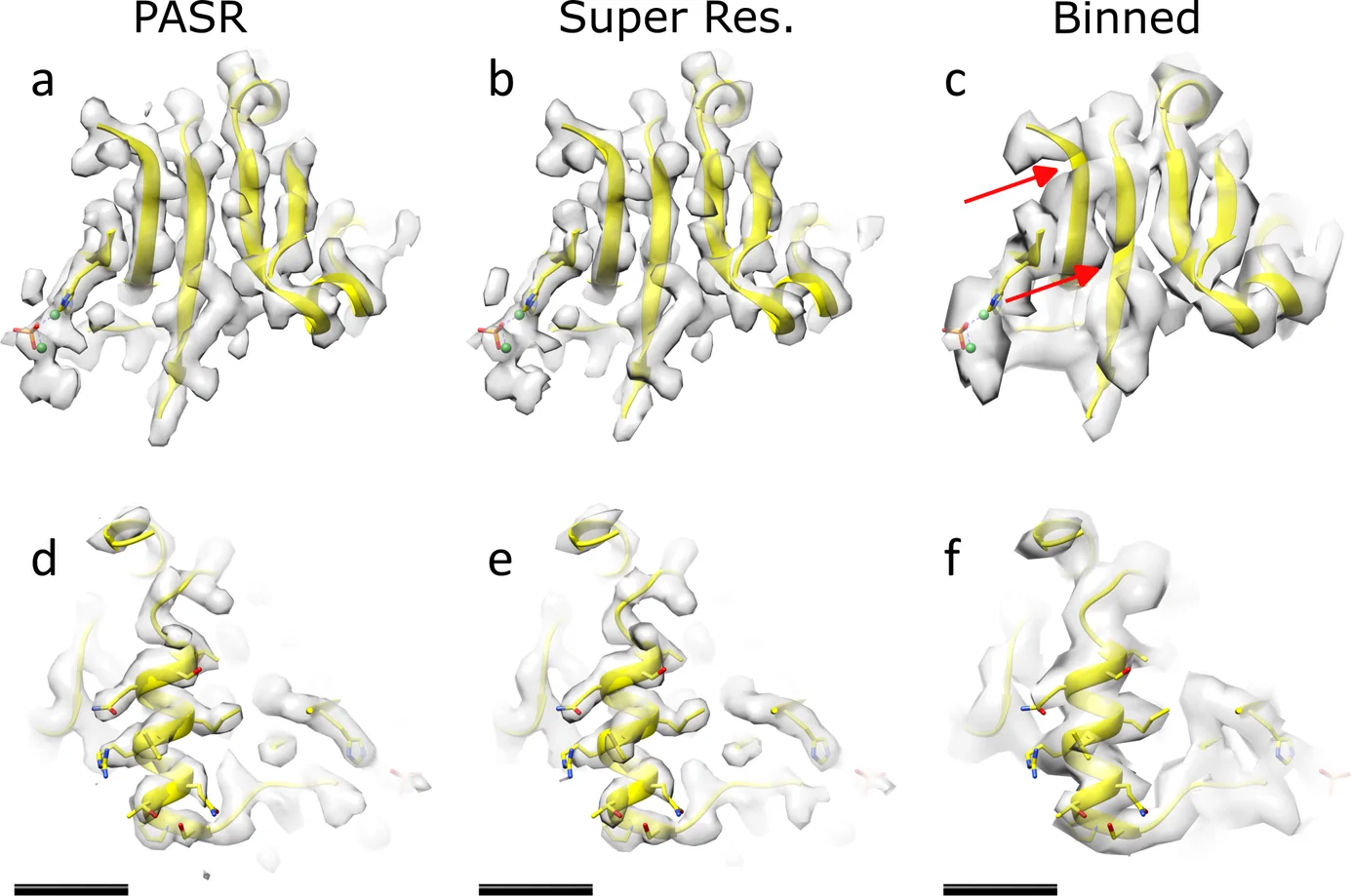
Comparing density of jack bean urease; main-chain density of jack bean urease in a β-sheet fold. (*a*) PASR-processed map, (*b*) super resolution map (original data), and (*c*) map at the physical Nyquist limit. Note the two main-chain density breaks (red arrows) present in the downsampled data, which are not evident for PASR or super resolution. (*d*) Another region of jack bean urease, focusing on an α-helix with side chains also shown. Clear side-chain density is evident for PASR (*d*) and super resolution data (*e*), commensurate with the reported resolution. The Nyquist-frequency-limited data (*f*) show ambiguous density for side chains, particularly the two glutamine residues and the arginine residue. Fitted PDB ID: 31a4. Once again, here, PASR is clearly competitive with super resolution acquisition, and a large improvement over data limited by the physical sampling frequency. Density displayed at 5σ. Scale bars are 1 nm.

The AAV dataset (Supplementary Fig. 9) was acquired with a different microscope and detector, and AAV is much smaller than Melbournevirus. Therefore, the dataset requires no block-based reconstruction (Zhu *et al.*, 2018 ▸) to process. The AAV dataset was acquired with a moderately high defocus; nevertheless, it still exceeded the original sampling limit (Supplementary Fig. 10). High symmetry means increased likelihood in achieving close to the Nyquist limit, as symmetry acts as a particle multiplier. The urease dataset (Fig. 6 ▸, Supplementary Fig. 11) was chosen as it is the only public dataset that exceeds the physical Nyquist limit; however, it had the second advantage of being (relatively) low symmetry, thus permitting us to examine the effects of PASR on lower-symmetry samples, where it still provides benefits (Fig. 6 ▸, Supplementary Fig. 12). The downsampled data provided a benchmark for how non-super-resolution data would perform (Supplementary Fig. 13) and curiously demonstrated some breaks in the main chain of the structure [Fig. 6 ▸(*c*)], which neither the super resolution nor the PASR data demonstrated [Fig. 6 ▸(*a*, *b*)].

There are now several neural-network-based approaches available for post-processing cryo-EM maps, such as *DeepEMhancer* (Sanchez-Garcia *et al.*, 2021 ▸), *SuperEM* (Venkata Subramaniya *et al.*, 2021 ▸) and *EMReady* (He *et al.*, 2023 ▸). The aim of each is to improve the clarity of less well resolved regions in the final output. The target of these tools is different to that of PASR, being already-generated maps and without the need to return to raw data, but it did cause us to ask whether zero padding the half maps would result in an improvement in the map the way PASR does. Zero padding half maps before FSC curve calculation results in no improvement in the unmasked FSC [green curve, Supplementary Fig. 21(*a*)] as the values are simply zero. However, zero padding does exhibit a clear effect on the phase-randomized FSC [red curve, Supplementary Fig. 21(*a*)], and consequently the masked map FSC [blue curve, Supplementary Fig. 21(*a*)] and corrected FSC [black curve, Supplementary Fig. 21(*a*)]. With zero-padded half maps, local resolution estimation estimated 2.2 Å for the capsid of the Melbournevirus block [Supplementary Fig. 21(*b*)], but the FSC is reported as 4.42 Å (the original sampling limit) and visual examination of the map looks like 4.4 Å [Supplementary Fig. 21(*c*)].

While initial model generation in the PASR-processed jack bean urease (Supplementary Fig. 12) dataset failed using the *RELION* 3.1 stochastic gradient descent algorithm, it succeeded using *cisTEM* (Grant *et al.*, 2018 ▸) and succeeded in a subsequent re-run. As such, given the challenges of initial model generation – and the fact that it is usually carried out on binned data – we do not consider this to be a consequence of PASR processing.

PASR does not currently compete ‘neck-and-neck’ with data collected natively at the equivalent higher magnification; however, the improvement over non-super-resolution data is clear (Figs. 5 ▸–8 ▸ ▸ ▸, Supplementary Figs. 1–16). While reporting gold-standard FSCs around 0.07–0.2 Å lower than native super resolution data or natively higher magnification data, side-chain clarity is comparable, which does make us question how valuable the reported (global) resolution is as a metric of performance. In effect, there are definitive ‘bands’ across the resolution range (Rosenthal & Rubinstein, 2015 ▸) at which point features are lost or gained, but within that band minor changes in reported resolution mean little (Figs. 5 ▸–7 ▸ ▸). The *B* factor is also marginally degraded (Supplementary Tables 1–7), although the impact of this is debatable as there is no hard and fast rule regarding what sharpening should be applied to a map. The idea that ‘closer to zero is better’ applies, but the *B* factor will vary depending on how it is estimated and between datasets or will be manually adjusted for clarity. Those aiming for record-breaking resolutions of smaller subjects should still collect data at higher magnification. Future adjustments to how PASR pre-processes data may improve upon this, but, at least initially, we feel a more cautious approach should be taken. We have several ideas for improving the current implementation of PASR which we hope to examine, including frame interpolation (roughly the same idea as cryo-ZSSR, but less demanding to implement), sub-pixel assignment and/or randomization and noise simulation as different noise models impact data differently, and raw cryo-EM data remain dominated by noise from different sources.

Collecting at a lower magnification means more particles per micrograph, which will play a significant role in increasing the particle count for giant virus cryo-EM SPA. Furthermore, if collecting data at a magnification that causes the sampling limit to fall between one of the previously defined resolution zones for map analysis (Rosenthal & Rubinstein, 2015 ▸), using PASR will aid identification of potentially unidentified capsid proteins – until recent success with interactomics work (Mühlberg *et al.*, 2025 ▸), identification of unknown capsid proteins was a laborious, ‘brute force’ process, even with help from programs such as *Model-Angelo* (Jamali *et al.*, 2023 ▸). Similarly, for heterogeneous samples [for example, V-ATPase (Burton-Smith *et al.*, 2023 ▸)], where teasing out rare states is critically dependent on collecting as many particles as possible, PASR should find utility. Indeed, processing the *E. hirae* V-ATPase would have been less demanding if we had been able to collect one quarter the number of micrographs, or improve separation of the sub-states with four times the number of particles!

The required storage space is decreased, as it is not necessary to record and archive raw super resolution micrograph movies. As these are approximately four times the size of counting mode micrographs (when using the same storage format) this should ease the burden on cryo-EM facilities for data storage and archival. What is chosen will depend on the equipment available to the facility, the nature of the sample and the ultimate objective of the study.

The PASR pre-processing step can take some time using the original scripts. Particularly, there is a significant bottleneck in reading and writing TIFF stack files using GNU Octave, although incorporating this method into a native program would solve this, as writing TIFF files is much faster in the *mrc2tif* program of *IMOD* (Kremer *et al.*, 1996 ▸), for example. The Octave version permitted step-by-step validation that the PASR pre-processing was happening as intended. A Python implementation is complete, which can be applied much more quickly and is available on GitHub, and was applied to the PASR-on-ASR dataset. Use of this version of PASR is recommended. We want to implement PASR into the processing suite pipelines natively, such that it could be applied in volatile memory during motion correction, further saving storage space. Alternatively, rather than being required to completely reprocess a dataset, it may be better to implement PASR at a later stage in the processing pipeline, perhaps as an option akin to Bayesian polishing (Zivanov *et al.*, 2019 ▸).

PASR is weakest when applied to single-frame (pre-motion corrected) micrographs, although even then it is still ahead of the resolution limited data for apoferritin (Supplementary Fig. 17) and nodavirus (Supplementary Figs. 18 and 19). However, there should be few occasions where a user is reaching the Nyquist limit with single-frame data. In further work, we would like to investigate further how much global motion across a micrograph aids or hinders PASR, but modern stages are generally stable so it would require sorting through a large quantity of data manually to isolate low-motion, medium-motion and high-motion movies.

**Figure 7:**
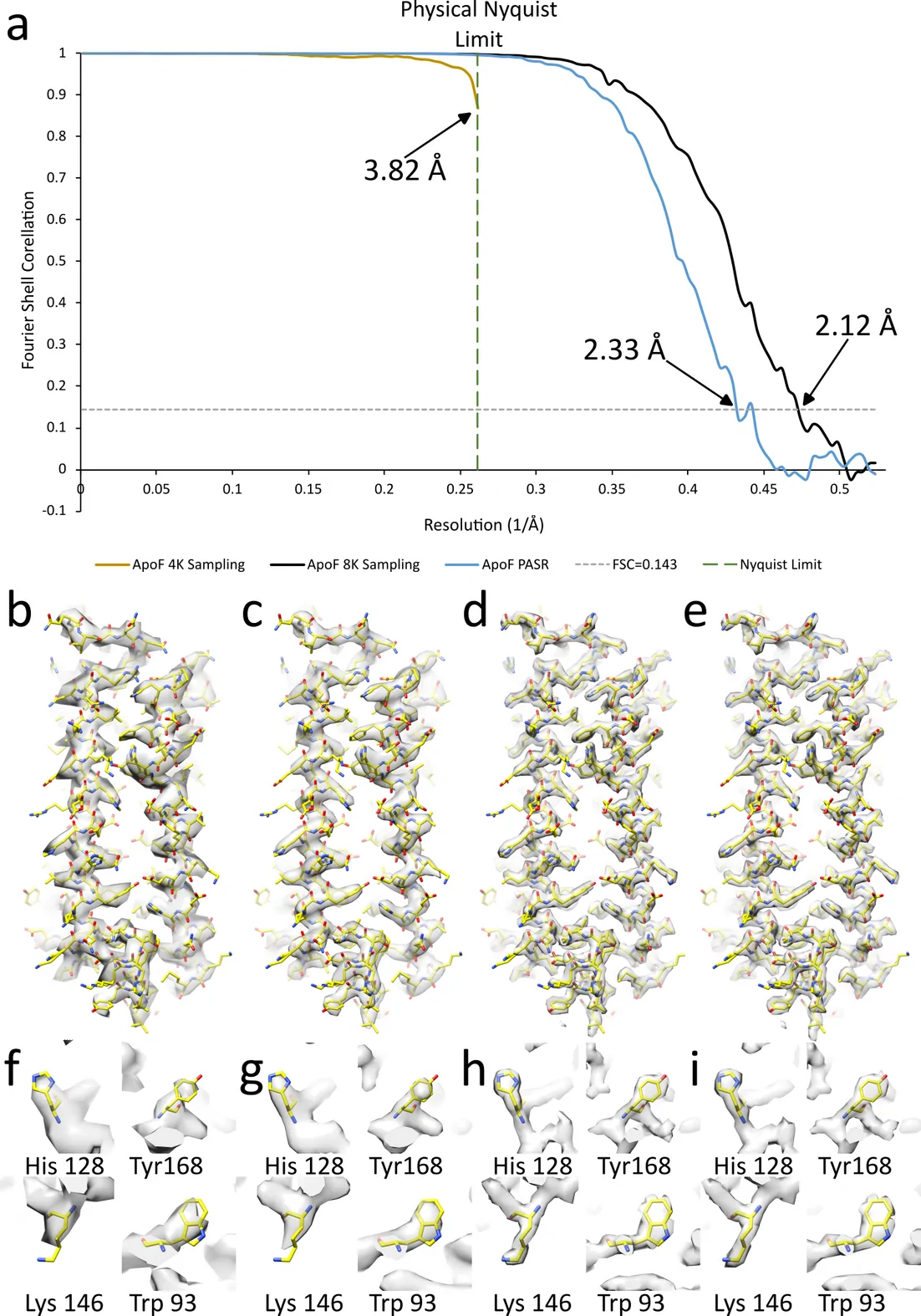
The improvement in resolution with PASR for apoferritin when acquired with conditions that image almost an entire grid hole. (*a*) FSC curves of the 4K sampled original data (mustard yellow curve), 8K sampled original data (black curve) and PASR-processed data (blue curve). FSC = 0.143 is indicated with a dashed grey line and the physical Nyquist limit is indicated with a dashed green line. This is a ‘worst case scenario’ for PASR, approximately 0.2 Å lower resolution than ‘native’ super resolution data. The same data processed with *Cryo­SPARC* are approximately 0.15 Å lower resolution than the native super resolution data. (*b*) Monomer of apoferritin with monomer rigid-body fitted, (*c*) the same monomer but resampled to the same pixel spacing as PASR/super resolution data, showing no visible improvement in map clarity, (*d*) monomer of apoferritin with monomer rigid-body fitted from super resolution (8K sampling) processing, and (*e*) monomer of apoferritin with monomer rigid-body fit from PASR processing. (*f*–*i*) Example side-chain densities for each of (*b*–*e*). PASR is clearly competitive with the super resolution (8K) sampled data. Density displayed at 5σ.

**Figure 8:**
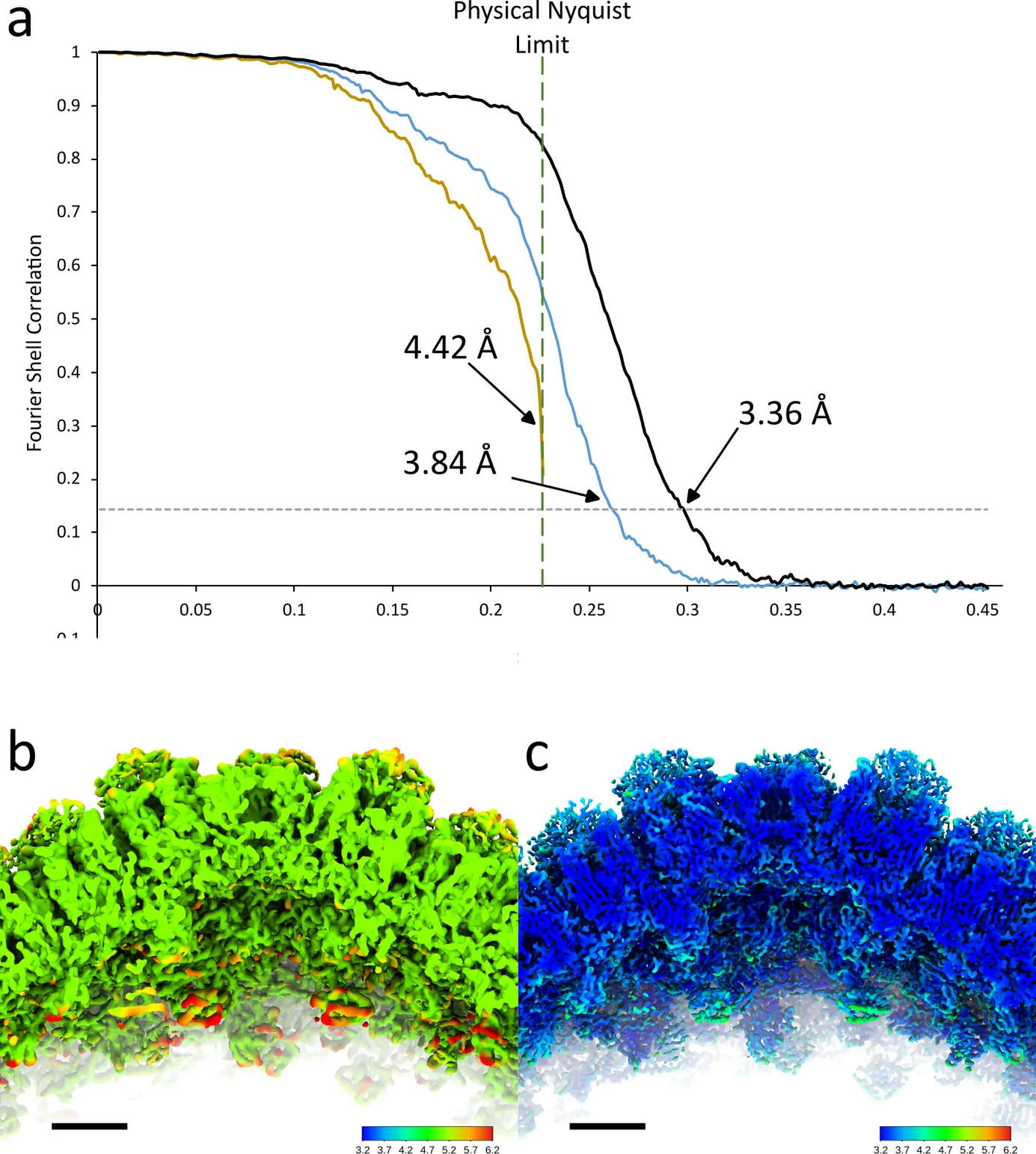
The improvement in resolution with PASR for Melbournevirus, which had originally been limited by sampling frequency during acquisition. (*a*) Gold-standard FSC curves of the fivefold block, showing the potential improvement from application of PASR. Original curve (mustard yellow line), PASR FSC before Bayesian polishing is carried out (pale blue line) and PASR FSC (black line). (*b*) Locally filtered map coloured by local resolution of a central slice of the original fivefold block. (*c*) Locally filtered map coloured by local resolution of a central slice of the PASR fivefold block. Maps shown at 3σ. Scale bars are 5 nm. Colour scales are identical and the colours range from 3.2 Å (blue) through to 6.2 Å (red). The improvement in main-chain clarity is dramatic in the capsid, making model building for proteins of undetermined sequence much more feasible.

PASR can be utilized on data already acquired with acquisition-time super resolution at lower magnifications resulting in reaching the Nyquist limit in (non-downsampled) super resolution data (Supplementary Fig. 22). This allows breaking of the super resolution Nyquist limit with data acquired from the Gatan K3 direct detector, which was previously impossible. Thus, PASR brings the K3 to parity with the Falcon 4(i) direct detector for potential at DSR modes. The only drawback for PASR-on-ASR data is the extreme memory requirements of Bayesian polishing, although this will likely not be an issue for long as computing resources continue to increase in capacity. Our work (Burton-Smith *et al.*, to be published; EMD-36418) demonstrates that it is possible to exceed the super resolution Nyquist limit. We briefly tested *RELION* 4 and the *VDAM* algorithm (Fig. 7 ▸, Supplementary Figs. 6–8), along with *Cryo­SPARC* (Supplementary Figs. 1 and 2) on PASR data with comparable results to *RELION* 3.1 (Figs. 5 ▸, 6 ▸, 8 ▸, Supplementary Figs. 3–5 and 14–21).

Concerns about overfitting are alleviated by the ‘sub-pixel flipping’ tests and comparing map-to-model FSCs and *Q*-scores of models when compared between PASR and other (counting mode and super resolution) data (Supplementary Figs. 23–27), which demonstrate similar behaviours.

Cryo-ZSSR (Huang *et al.*, 2023 ▸) follows a similar concept to PASR but utilizes a neural network implementation. Examination of the Cryo-ZSSR documentation suggests that it has several other limitations not evident with PASR. The movies require complex pre-processing to generate intermediate movies; for each intermediate movie frame generated the whole cryo-ZSSR pre-processing step must be repeated, rather than the simpler pixel doubling implementation of PASR followed by a standard cryo-EM processing pipeline. In large datasets containing micrographs with many frames this will be slower than either the current PASR implementation or PASR purely in volatile memory. In our experiments, PASR reports resolutions closer to the original data than cryo-ZSSR appears to, although we are intrigued by their method.

Large cryo-EM facilities face ever increasing storage demands for raw data, so they may find PASR attractive for samples known to be heterogeneous. Further, facilities that do not have detectors capable of super resolution acquisition may also find PASR of interest – one of the highest-resolution cryo-EM reconstructions published at time of writing is from data collected from a Falcon 3 (Yip *et al.*, 2020 ▸) – so detectors without super resolution acquisition are still extremely capable. In conclusion, we envision this technique to be of greatest utility to cryo-EM researchers working on giant structures where the number of objects that can be imaged on each micrograph is limited, but hope others may find the technique of use.

## 4. Materials and methods

### 4.1. Datasets used

Table 1 ▸ details the datasets used. We chose to test a variety of data based on the use of different detectors for acquisition. EMPIAR-10216 (Danev *et al.*, 2019 ▸) was used for apoferritin, as it was acquired using a Falcon 3 direct detector, EMPIAR-10549 (Feathers *et al.*, 2021 ▸) was used to test both a direct comparison with K3 super resolution data and a lower symmetry complex. EMPIAR-10203 (Ho *et al.*, 2018 ▸) was used for testing the efficacy of PASR when applied to single-frame K2 micrographs. We were granted permission to test the adeno-associated virus dataset by Professor Uchiyama (University of Osaka), which was collected from a JEOL CRYOARM 300 microscope using a Gatan K3 detector in CDS (correlative double sampling) mode. Melbournevirus was previously processed (Burton-Smith *et al.*, 2026 ▸). The first 32 micrographs of an apoferritin dataset acquired with a Falcon 4 DED used in Burton-Smith *et al.* (to be published) were tested as they allowed us to test both ‘native’ (4K) and super resolution (8K) processing from a single dataset without requiring binning. All datasets were processed with the ‘Ignore CTFs until first peak?’ parameter set to ‘Yes’ in the *RELION* user interface.

### 4.2. Pre-processing

A script was written in GNU Octave (Eaton *et al.*, 2019 ▸; Eaton *et al.*, 2022 ▸) supplemented by the *Image*package (https://gnu-octave.github.io/packages/image/), and the *ReadMRC*, *WriteMRC* and *WriteMRCHeader* functions developed for *MATLAB* (The MathWorks, 2022 ▸) by F. Sigworth (Sigworth, 2023 ▸) to import micrograph movies, divide each pixel into a 2 × 2 grid with each sub-pixel having an identical value to the originating pixel, and write the resulting ‘super resolution’ micrograph movie to a different file. A modified form, using the *ReadTIFFStack* function, was used for TIFF format micrographs. This script carried out ‘pixel doubling’ on each frame of every micrograph movie as described above. These pre-processed movies were passed to the standard *RELION* (Fernandez-Leiro & Scheres, 2017 ▸) processing pipeline. *RELION* 3.1 was used except for those cases for which *RELION* 4 or *Cryo­SPARC* are explicitly stated. To test EMPIAR-10216 and EMPIAR-10549 with PASR, another script was written which first binned down each 2 × 2 area to a single pixel by Fourier space cropping. These downsampled micrograph movies were then reprocessed with the PASR script. The publicly available Python-based script (see *Data Availability*) is recommended as the output is identical, but processing is an order of magnitude faster.

### 4.3. Processing of the apoferritin dataset Apo64K from the Titan Krios G4 at the National Institute for Physiological Sciences (NIPS), Japan

These data were processed in *RELION* 4. Supplementary Table 2 summarizes this. The 4K sampled original data were imported and motion corrected with the *RELION* (Zivanov *et al.*, 2018 ▸) implementation of the *MotionCor2* algorithm (Zheng *et al.*, 2017 ▸) with 32-bit MRC output and separate power spectra. Separate power spectra and the micrographs reported highly similar CTF value estimates with *CTFFIND* (Rohou & Grigorieff, 2015 ▸). A small number of particles were manually picked and classified, before template picking was used to select a total of 102 364 particles. After 2D classification, 33 468 particles were selected in clear classes and an initial model generated. After a single round of 3D refinement, the reconstruction reached the physical Nyquist limit.

The 8K sampled original data were imported and motion corrected with the *RELION* (Zivanov *et al.*, 2018 ▸) implementation of the *MotionCor2* algorithm (Zheng *et al.*, 2017 ▸) with 32-bit MRC output and separate power spectra. Separate power spectra and the micrographs reported highly similar CTF value estimates with *CTFFIND* (Rohou & Grigorieff, 2015 ▸). A small number of particles were manually picked and classified, before template picking was used to select a total of 84 586 particles. After 2D classification, 63 313 particles were selected in clear classes and an initial model generated. After cycling 3D refinement and CTF refinement (magnification anisotropy, beam tilt, per-particle defocus and astigmatism), followed by Bayesian polishing, the final gold-standard FSC reported a resolution of 2.13 Å with a *B* factor of −62.4.

The PASR-processed data were imported and motion corrected with the *RELION* (Zivanov *et al.*, 2018 ▸) implementation of the *MotionCor2* algorithm (Zheng *et al.*, 2017 ▸) with 32-bit MRC output and separate power spectra. Separate power spectra and the micrographs reported highly similar CTF value estimates with *CTFFIND* (Rohou & Grigorieff, 2015 ▸). A small number of particles were manually picked and classified, before template picking was used to select a total of 102 493 particles. After 2D classification, 64 597 particles were selected in clear classes and an initial model generated. After cycling 3D refinement and CTF refinement (magnification anisotropy, beam tilt, per-particle defocus and astigmatism), followed by Bayesian polishing, the final gold-standard FSC reported a resolution of 2.33 Å with a *B* factor of −99.

The 8K sampled original data were imported into *Cryo­SPARC* (4.2.1) (Punjani *et al.*, 2017 ▸) and patch motion correction and patch CTF estimation were carried out with the default parameters. Blob picking was used, selecting a ring blob of internal diameter 90 Å and external diameter 110 Å, which selected 57 234 particles. Particles were extracted and 2D classified into 100 classes with 250 particles per class and a maximum resolution of 4 Å, which seems to distinguish apoferritin classes more clearly. The clear classes, containing 47 695 particles, were passed to *ab initio* model generation and homogeneous refinement with defocus and beam tilt optimization enabled, resulting in a final resolution of 2.28 Å with a *B* factor of −74.8.

The PASR-processed data were imported into *Cryo­SPARC* (4.2.1) (Punjani *et al.*, 2017 ▸) and patch motion correction and patch CTF estimation were carried out with the default parameters. Blob picking was used, selecting a ring blob of internal diameter 90 Å and external diameter 110 Å, which selected 57 988 particles. Particles were extracted and 2D classified into 100 classes with 250 particles per class and a maximum resolution of 4 Å, which seems to distinguish apoferritin classes more clearly. The clear classes, containing 45 783 particles, were passed to *ab initio* model generation and homogeneous refinement with defocus and beam tilt optimization enabled, resulting in a final resolution of 2.45 Å with a *B* factor of −114.8. *Cryo­SPARC* processing parameters are summarized in Supplementary Table 7.

### 4.4. Processing of the apoferritin dataset (EMPIAR-10216)

The original data, the downsampled data and the PASR data were processed via normal *RELION* processing. Supplementary Table 2 summarizes this. The original dataset was processed by importing all micrographs into *RELION* 3.1 and following a standard processing pipeline of motion correction and CTF estimation, of which 1228 micrographs passed CTF estimation with a resolution less than 5 Å. Template-based particle picking selected 217 495 particles, of which 161 966 were selected in clear classes after 2D classification. 3D classification was carried out for initial particle alignment before 3D refinement. Parameter optimization with CTF refinement and Bayesian particle polishing was carried out before an Ewald sphere curvature correction was applied to the final reconstruction to a final resolution of 1.7 Å with an estimated *B* factor of −44.4.

The downsampled dataset was processed in the same way, with 1153 micrographs passing CTF estimation with a 5 Å cutoff, with template autopicking selecting 156 099 particles. 149 536 particles were selected in clear classes after 2D classification, before 3D classification and refinement. After post processing this refinement reached the downsampled sampling limit before any CTF refinement, polishing or Ewald sphere curvature correction was required, so they were not applied. The final resolution was 2.068 Å with an estimated *B* factor of −46.8.

The PASR dataset was also processed in the same way. After motion correction and CTF estimation, 1106 micrographs had an estimated CTF resolution of less than 5 Å. Template autopicking selected 207 388 particles. 152 367 particles were selected in clear classes, although in the 2D classification step the number of clear classes was significantly reduced compared with both the original and binned data. After CTF refinement (magnification anisotropy, beam tilt and particle defocus/astigmatism refinement), Bayesian polishing and Ewald sphere curvature correction, the final resolution was 1.78 Å with an estimated *B* factor of −50.2.

### 4.5. Processing of the jackbean urease dataset (EMPIAR-10549)

The original data, the downsampled data and the PASR data were processed via normal *RELION* processing. Supplementary Table 3 summarizes this.

The original dataset was processed by importing 286 micrographs and motion correcting with *MotionCor2* (Zheng *et al.*, 2017 ▸). After CTF estimation, 281 micrographs demonstrated good fits. LoG particle picking was used with a minimum diameter of 110 Å and a maximum diameter of 130 Å. 1 175 198 particles were extracted and 2D classified with clear classes selected containing 837 778 particles. An initial model was generated with D3 symmetry, which was used as a reference for initial alignment with 3D classification before 3D refinement. Parameter refinement was carried out to optimize per-particle defocus and astigmatism, magnification anisotropy and beam tilt. Bayesian polishing was carried out before post processing estimated a final resolution of 3.07 Å with an estimated *B* factor of −156.7.

The downsampled dataset was processed by importing the micrographs and motion correcting with *MotionCor2* (Zheng *et al.*, 2017 ▸). After CTF estimation, 279 micrographs passed. LoG picking was used, with a minimum diameter of 110 Å and a maximum diameter of 130 Å, for a total of 1 170 221 particles, of which 747 661 were within clear classes after 2D classification. An initial model was generated and used as an initial reference in 3D classification. After a single 3D refinement, the physical Nyquist limit was reached of 4.2 Å with an estimated *B* factor of −218.8.

The PASR dataset was processed by importing 286 micrographs and motion correcting with *MotionCor2* (Zheng *et al.*, 2017 ▸). After CTF estimation, 280 micrographs were passed to LoG autopicking, with a minimum diameter of 110 Å and a maximum diameter of 130 Å, for a total of 1 173 581 particles. After 2D classification, 740 059 particles were contained within clear classes. Initial model generation in *RELION* failed twice, so the stack was exported using *relion_stack_create* and *cisTEM* (Grant *et al.*, 2018 ▸) was used, which successfully generated an initial model. This initial model was used for particle alignment with a single 3D classification before 3D refinement was carried out. Parameter optimization with CTF refinement (magnification anisotropy, beam tilt and per-particle defocus and astigmatism) was carried out followed by Bayesian polishing, resulting in a final map with a resolution of 3.19 Å with an estimated *B* factor of −178.4.

### 4.6. Processing of Melbournevirus

The original dataset was not reprocessed. Please see Burton-Smith *et al.* (2026 ▸) for further details of the original processing. The PASR dataset was processed in a manner as close to the original dataset as possible; however, particle selection was LoG picking only, followed by manual removal of particles at the edges of micrographs before 2D classification. This resulted in ∼800 more particles in the whole virus consensus reconstruction compared with our original work. Supplementary Table 4 summarizes this. As the dataset was collected originally on two different occasions and the two datasets contain different numbers of frames, when Bayesian polishing was carried out it was first carried out on the first dataset, then the second with the same parameters (*RELION* defaults), and the two sets of polished particles recombined for each focused block of the virus. The twofold block was processed with a box size of 800 pixels, the threefold block was processed with a box size of 900 pixels, and the fivefold block was processed with a box size of 660 pixels. These were chosen as a good balance of coverage of the viral capsid for the volume size and processing time, while allowing full coverage of the capsid.

### 4.7. Processing of adeno-associated virus (AAV)

The original data and the PASR data were processed normally in *RELION*. Supplementary Table 5 summarizes this. The original dataset was processed by importing all 6067 micrographs and motion correcting with *MotionCor2* (Zheng *et al.*, 2017 ▸). CTF estimation was carried out with *CTFFIND* 4.1 (Rohou & Grigorieff, 2015 ▸) and particles from the 6001 micrographs which passed CTF estimation were picked with the LoG autopicker (Zivanov *et al.*, 2018 ▸), with a minimum diameter of 230 Å and a maximum of 290 Å, resulting in a total of 1 473 552 picked particles. These particles were extracted and 2D classified into 30 classes, of which clear classes were selected containing a total of 1 090 590 particles. An initial model was generated from these particles and a 3D refinement carried out, which reached the maximum resolution of 2.68 Å with an estimated *B* factor of −98.8.

The PASR dataset was processed in the same way, with the following exceptions: we tested a second round of picking with a template picker, and the imposed symmetry was I3 rather than I1. This was because we wanted to test block-based reconstruction (Zhu *et al.*, 2018 ▸) with the smaller particle. However, block-based reconstruction of AAV was abandoned as too time-intensive. 5995 micrographs passed CTF estimation with the same resolution cutoff, were again autopicked using the LoG autopicker with the same parameters, extracted and 2× downsampled before 2D classification into 30 classes. We tested template autopicking with three varied averages, which resulted in better-centred picks and a total of 1 614 326 particles were selected. After 2D classification into 100 classes, 14 clear classes were selected containing a total of 1 100 376 particles. From these, an I3 symmetry initial model was generated, and a 3D refinement carried out. Particles were re-extracted unbinned and the refined map rescaled to act as a reference structure. CTF refinement (magnification anisotropy, beam tilt and defocus/astigmatism) was carried out before Bayesian polishing. The final map reached a gold-standard FSC resolution estimate of 2.17 Å with a *B* factor of −100.8.

### 4.8. Processing of nodavirus

Supplementary Table 6 summarizes the important processing statistics. Nodavirus micrographs from EMPIAR-10203 (Ho *et al.*, 2018 ▸) were imported into *RELION* and the CTF estimated with *CTFFIND* 4 (Rohou & Grigorieff, 2015 ▸). Particles were picked with the LoG autopicker with an inner diameter of 230 Å and an outer diameter of 290 Å. There was a total of 66 615 picks. These were extracted and 2D classified, with clear classes containing 29 180 particles. An initial model was generated and cycles of 3D refinement and CTF refinement carried out until a final resolution of 2.86 Å and *B* factor of −108.1.

The micrographs were binned, and the CTF estimated with *CTFFIND* 4 (Rohou & Grigorieff, 2015 ▸). The particle locations from the final output of the original data were rescaled to the smaller micrographs and the particles re-extracted. A single 3D refinement reached the binned Nyquist limit of 4.24 Å with an estimated *B* factor of −57.

The binned micrographs were PASR-processed and the CTF estimated using *CTFFIND* 4 (Rohou & Grigorieff, 2015 ▸). The particle locations from the final output of the original data were used and after a 3D refinement and single defocus optimization pass, the highest resolution achieved was 3.21 Å with a *B* factor of −143.9.

### 4.9. Processing of PASR-on-ASR apoferritin

Supplementary Table 8 summarizes the important processing statistics and model statistics. Supplementary Fig. 22 shows a representative micrograph after motion correction, the 2D classes, the map and the FSC curve. ASR apoferritin micrographs were collected on a CRYOARM 300 with a Gatan K3 direct detector and passed to the PASR Python script. The processing followed the same basic outline of the apoferritin processing described above. However, when Bayesian polishing was reached, the high particle count (>2000 particles per micrograph) and large dimensions (23 040 × 16 368 pixels and 110 frames) resulted in extremely high system memory usage. After Ewald sphere correction, the final gold-standard FSC resolution was 1.667 Å, far exceeding both the physical and super resolution sampling limits.

### 4.10. Model building and validation

The model for apoferritin was built using the mouse heavy chain apoferritin sequence and refined using *ServalCat* (Yamashita *et al.*, 2021 ▸) and *Coot* (Emsley & Cowtan, 2004 ▸; Casañal *et al.*, 2019 ▸). *Q*-scores were calculated with the *ChimeraX* (Goddard *et al.*, 2018 ▸) *Q*-score plugin (Croll, 2024 ▸). Map-to-model FSCs were calculated with *Servalcat* (Yamashita *et al.*, 2021 ▸).

### 4.11. Visualization

Viewing of 2D classes *etc*. was carried out within either *RELION* (Kimanius *et al.*, 2021 ▸; Scheres, 2012 ▸; Zivanov *et al.*, 2020 ▸) or *Cryo­SPARC* (Punjani *et al.*, 2017 ▸). 3D volumes were visualized in *UCSF Chimera* (Pettersen *et al.*, 2004 ▸; Goddard *et al.*, 2007 ▸) and *UCSF ChimeraX* (Goddard *et al.*, 2018 ▸). *SEGGER* (Pintilie & Chiu, 2012 ▸) was used to segment maps.

## Supplementary Material

Supplementary tables and figures. DOI: 10.1107/S2052252526005348/rq5016sup1.pdf

PDB reference: apoferritin PASR-on-ASR, 9wal

EMDB reference: apoferritin PASR-on-ASR, EMD-65822

## Figures and Tables

**Table 1:** Datasets used in this work CDS = correlative double sampling.

Dataset	Apoferritin (64K)	Apoferritin	Melbournevirus	Adeno-associated virus	Jack-bean urease	Nodavirus	Apoferritin (PASR-on-ASR)
Source	This study	EMPIAR-10216 (Danev *et al.*, 2019)	Burton-Smith *et al.* (2026)	This study	EMPIAR-10549 (Feathers *et al.*, 2021)	EMPIAR-10203 (Ho *et al.*, 2018)	This study
Physical pixel size (Å pixel^−1^)	1.912	0.517	2.21	1.34	2.1	1.06	1.9708
Microscope	Titan Krios G4 (300 kV)	Titan Krios G3 (300 kV)	Titan Krios G2 (300 kV)	CRYOARM 300 (300 kV)	Tecnai Arctica (200 kV)	Titan Krios (300 kV)	CRYOARM 300 (300 kV)
Detector	Falcon 4	Falcon 3	K2 Summit	K3	K3	K2 Summit	K3 BioQuantum
Acquisition mode	Counting	Counting	Counting	CDS	Super resolution	Counting	Super resolution
Format	EER (converted to TIFF)	MRC (gain corrected)	MRC (gain corrected)	TIFF	TIFF	MRC (single frame)	TIFF
Movies or micrographs	Movies	Movies	Movies	Movies	Movies	Micrographs	Movies
Processing carried out	4K, 8K, PASR	Original, binned, PASR	PASR	Original, PASR	Original, binned, PASR	Original, PASR	PASR
Relevant figures	7, S1–5	5, S14–16	8, S6–8	S9–10	6, S11–13	S18–20	S22

## Data Availability

The PASR scripts are available from https://github.com/rbs-sci/PASR. The pixel flipping script is available from https://github.com/rbs-sci/pixelFlip. Post-processed maps, half maps and FSC curves for each of the reconstructions are available from the EMDB under the following accession codes: EMPIAR-10216 (PASR): 37171, EMPIAR-10216 (reprocessing): 37172, EMPIAR-10216 (binned): 37173, EMPIAR-10549 (PASR): 37185, EMPIAR-10549 (reprocessing): 37186, EMPIAR-10549 (binned): 37187, EMPIAR-10203 (reprocessing): 37181, EMPIAR-10203 (PASR): 37182, EMPIAR-10203 (binned): 37183, apoferritin (64 000×) (0.956 Å pixel^−1^) PASR: 37174, apoferritin (64 000×) (0.956 Å pixel^−1^) 8K: 37175, apoferritin (64 000×) (1.91 Å pixel^−1^) 4K: 37176, apoferritin (64 000×) (0.956 Å pixel^−1^) 8K (*Cryo­SPARC*): 37177, apoferritin (64 000×) (0.956 Å pixel^−1^) PASR (*Cryo­SPARC*): 37178, apoferritin (64 000×) (0.956 Å pixel^−1^) single-frame (post-motion correction) PASR: 37184, adeno-associated virus (original): 37179, adeno-associated virus (PASR): 37180, Melbournevirus twofold block PASR: 37188, Melbournevirus threefold block PASR: 37189, Melbournevirus fivefold block PASR: 37190, apoferritin (PASR-on-ASR): 65822 and PDB ID: 9wal. The following post-processed maps and FSC curves had already been deposited as previous work with the EMDB under the following accession codes: Melbournevirus twofold block (original): 31531, Melbournevirus threefold block (original): 31530, Melbournevirus fivefold block (original): 31529. The following datasets are already publicly available on the EMPIAR database: 10203, 10216, 10549.
