# Supplementary material for: Post-acquisition super resolution for cryo-electron microscopy
Source: IUCrJ. 2026 Sep 2;13(Pt 5):589–605. doi: 10.1107/S2052252526005348 (PMC13548832; doi:10.1107/S2052252526005348)
Supplement: Supplementary file 1 [file m-13-00589-sup1.pdf]

IUCrJ

Volume 13 (2026)

Supporting information for article:

Post-acquisition super resolution for cryo-electron microscopy

Raymond N. Burton Smith and Kazuyoshi Murata

Table S1 Tabulated statistics for processing of 32 micrograph apoferritin dataset, comparing 4K (counting mode), 8K (super resolution) and PASR processing.

Apoferritin (this study) (RELION 4)			
	Original (4K)	Original (8K)	PASR
Accelerating voltage	300	300	300
Spherical aberration	2.7	2.7	2.7
Micrographs (total)	32	32	32
Magnification (Å/pixel)	1.912	0.956	0.956
Passed CTF estimation	32	32	32
CTF estimation cutoff (Å)	n/a	n/a	n/a
Particles picked	102,364	84,586	102,493
Particles selected	33,468	63,313	64,597
Final resolution (Å)	3.824	2.13	2.33
Final particle count	33,468	63,313	64,597
Estimated b-factor	-116.6	-62.4	-99
EMDB accession code	37176	37175	37174
Q-score (vs. 2CIH with H2O removed)	0.57	0.84	0.82
Map-to-model FSC (=0.5, vs. 2CIH with H2O removed)	n/a	2.12	2.32

Table S2 Tabulated statistics for processing of EMPIAR-10216 apoferritin dataset, comparing original data, Fourier cropped and Fourier-cropped PASR processing.

Apoferritin (EMPIAR-10216) (RELION 3.1)			
	Original	Binned	PASR
Accelerating voltage	300	300	300
Spherical aberration	2.7	2.7	2.7
Micrographs (total)	1,647	1,647	1,647
Magnification (Å/pixel)	0.517	1.034	0.517
Passed CTF estimation	1,228	1,153	1,106
CTF estimation cutoff (Å)	5	5	5
Particles picked	217,495	156,099	207,388
Particles selected	161,966	149,536	152,367
Final resolution (Å)	1.71	2.068	1.78
Final particle count	161,966	149,536	152,367
Estimated b-factor	-44.4	-46.8	-50.2
EMDB accession code	37172	37173	37171
Q-score (vs. 2CIH with H2O removed)	0.86	0.84	0.85
Map-to-model FSC (=0.5, vs. 2CIH with H2O removed)	1.71	2.068	1.79

Table S3 Tabulated statistics for processing of EMPIAR-10549 jackbean urease dataset, comparing original data, Fourier cropped and Fourier-cropped PASR processing.

Jack bean urease (EMPIAR-10549) (RELION 3.1)			
	Original	Binned	PASR
Accelerating voltage	200	200	200
Spherical aberration	2.7	2.7	2.7
Micrographs (total)	286	286	286
Magnification (Å/pixel)	1.05	2.1	1.05
Passed CTF estimation	281	279	280
CTF estimation cutoff (Å)	8	8	8
Particles picked	1175198	1170221	1173581
Particles selected	837778	747661	740059
Final resolution (Å)	3.07	4.2	3.19
Final particle count	837778	747661	740059
Estimated b-factor	-156.7	-218.8	-178.4
EMDB accession code	37186	37187	37185
Q-score (vs. 7KNS)	0.65	0.46	0.67
Map-to-model FSC (=0.5, vs. 7KNS)	3.2	n/a	3.4

Table S4 Tabulated statistics for processing of Melbournevirus dataset, comparing original (counting mode) data, and PASR processing.

Melbournevirus (RELION 3.1)		
	Original	PASR
Accelerating voltage	300	300
Spherical aberration	2.7	2.7
Micrographs (total)	3,367	3,367
Magnification (Å/pixel)	2.21	1.105
Passed CTF estimation	3,067	3,140
CTF estimation cutoff (Å)	Manual	10
Particles picked	7,000	7,747
Particles selected	3,852	5,312
Final resolution (2F/3F/5F) (Å)	4.43/4.42/4.42	3.48/3.48/3.36
Final particle count (sym. expanded)	3,124 (187,440)	3,919 (235,140)
Estimated b-factor	-100 (imposed)	-100 (imposed)
EMDB accession codes (2F/3F/5F)	31531/31530/31529	37188/37189/37190
Q-score (7YJL) fitted to threefold block central axis	0.5	0.64

Table S5 Tabulated statistics for processing of Adeno associated virus dataset, comparing original (correlative double sampling) data, and PASR processing.

Adeno associated virus (RELION 3.1)		
	Original	PASR
Accelerating voltage	300	300
Spherical aberration	2.7	2.7
Micrographs (total)	6,067	6,067
Magnification (Å/pixel)	1.34	0.67
Passed CTF estimation	6,001	5,995
CTF estimation cutoff (Å)	Manual	Manual
Particles picked	1,473,552	1,614,326
Particles selected	1,090,590	1,100,376
Final resolution (Å)	2.68	2.16
Final particle count	1,090,590	1,023,144
Estimated b-factor	-98.8	-100.8
EMDB accession code	37179	37180
Q-score (vs. Model-Angelo PDB)	0.79	0.87
Map-to-model FSC (=0.5, vs. Model-Angelo PDB)	n/a	2.16

Table S6 Tabulated statistics for processing of EMPIAR-10203 Nodavirus dataset, comparing original data, Fourier cropped data and PASR processing.

Nodavirus (EMPIAR-10203) (RELION 3.1)			
	Original	Binned	PASR
Accelerating voltage	300	300	300
Spherical aberration	2.7	2.7	2.7
Micrographs (total)	2,459	2,459	2,459
Magnification (Å/pixel)	1.06	2.12	1.06
Passed CTF estimation	2,457	n/a	n/a
CTF estimation cutoff (Å)	Manual	n/a	n/a
Particles picked	66,615	n/a	n/a
Particles selected	29,180	Used original particle stack	Used original particle stack
Final resolution (Å)	2.86	4.24	3.21
Final particle count	29,180	n/a	n/a
Estimated b-factor	-108.1	-57	-143.9
EMDB accession code	37181	37183	37182
Q-score (vs. 6H2B)	0.56	0.49	0.54
Map-to-model FSC (=0.5, vs. 6H2B)	3.02	n/a	3.3

Table S7 Tabulated statistics for processing of apoferritin dataset (Table S1) in CryoSPARC, comparing 8K (super resolution) data, and 4K-PASR processing.

Apoferritin (this study) (CryoSPARC)		
	Original (8K)	PASR
Accelerating voltage	300	300
Spherical aberration	2.7	2.7
Micrographs (total)	32	32
Magnification (Å/pixel)	0.956	0.956
Passed CTF estimation	32	32
CTF estimation cutoff (Å)	n/a	n/a
Particles picked	57,234	57,988
Particles selected	47,695	45,783
Final resolution (Å)	2.28	2.45
Final particle count	47,695	45,783
Estimated b-factor	(-)74.8	(-)114.4
EMDB accession code	37177	37178
Q-score (vs. 2CIH with H2O removed)	0.81	0.79
Map-to-model FSC (=0.5, vs. 2CIH with H2O removed)	2.27	2.48

Table S8 Tabulated statistics for processing of super resolution apoferritin dataset with PASR-on-ASR.

	PASR-on-ASR (this study) (RELION 3.1)	
Accelerating voltage	300	
Spherical aberration	2.7	
Micrographs (total)	597	
Magnification (Å/pixel)	1.9708 (physical pixel), 0.9854 (super resolution pixel)	
Passed CTF estimation	597	
Particles picked	3,234,179	
Final resolution (Å)	1.667	
Final particle count	2,910,547	
Estimated b-factor	-60.4	
EMDB accession code	65822	
PDB accession code	9WAL	
Ramachandran outliers (and favoured)	0% (98.26% favoured)	0% (98.26% favoured)
Rotamer outliers	0	0
C-beta deviations	0	0
Clash score	0.33	0.69
MolProbity score	0.62	0.72
Q-score	0.91	0.91
Map-to-model FSC (FSC = 0.5)	1.68	1.68

Table S9 Tabulated statistics for pixel flipping tests for various reconstructions. Reported resolutions are pre-Bayesian polishing. Where two numbers are reported, the first is from simple reconstruction, the second from re-refinement.

Map	Map-to-map FSC (reconstructed/re-refined) (Å) (FSC = 0.143)	Map-to-model FSC (reconstructed/re-refined) (Å) (FSC = 0.5)	Q-score (reconstructed/re-refined)	B-factor (reconstructed/re-refined)
Jack-bean urease (original data) (super resolution)	3.19	3.45	0.65	-204
Jack-bean urease (column flipped) (super resolution)	3.5/3.5	3.7/3.7	0.63/0.62	-228/-246
Jack-bean urease (row flipped) (super resolution)	3.5/3.5	3.7/3.7	0.63/0.61	-211/-234
Jack-bean urease (column & row flipped) (super resolution)	4.06/4.06	4.2/4.2	0.55/0.43	-257/-311
Jack-bean urease (PASR)	3.40	3.63	0.66	-239
Jack-bean urease (PASR) (column flipped)	3.73/3.73	3.95/3.9	0.62/0.61	-248/-272
Jack-bean urease (PASR) (row flipped)	3.73/3.68	3.95/3.9	0.61/0.61	-260/-275
Jack-bean urease (PASR) (column & row flipped)	4.14/4.14	4.2/4.26	0.55/0.54	-332/-331
4K high-magnification Apoferritin (original)	1.98/1.95	1.99/1.98	0.84/0.84	-37-38

4K high-magnification Apoferritin (column flipped)	2.23/2.27	2.26/2.26	0.81/0.81	-48/-51
4K high-magnification Apoferritin (row flipped)	2.21/2.17	2.24/2.24	0.81/0.82	-51/-49
4K high-magnification Apoferritin (column & row flipped)	2.6/2.6	2.62/2.62	0.78/0.77	-70/-69
4K medium magnification Apoferritin (original) (vs. 2CIH with H2O removed)	3.82	n/a	0.57	-94
4K medium magnification Apoferritin (column flip) (vs. 2CIH with H2O removed)	5.1/4.99	n/a	0.03/0.35	-155/-422
4K medium magnification Apoferritin (row flip) (vs. 2CIH with H2O removed)	5.2/4.99	n/a	0.08/0.33	-148/-448
4K medium magnification Apoferritin (column and row flip) (vs. 2CIH with H2O removed)	3.82/7.42	n/a	0.14/0.02	-6.2/-136
8K medium magnification PASR Apoferritin (original)	2.33	2.35	0.82	-99

(vs. 2CIH with H2O removed)				
8K medium magnification PASR Apoferritin (column flip) (vs. 2CIH with H2O removed)	2.91/2.85	2.89/2.86	0.76/0.77	-149/-150
8K medium magnification PASR Apoferritin (row flip) (vs. 2CIH with H2O removed)	2.95/2.88	2.86/2.87	0.75/0.76	-147/-147
8K medium magnification PASR Apoferritin (column and row flip) (vs. 2CIH with H2O removed)	3.45/3.4	3.38/3.4	0.68/0.69	-196/-209

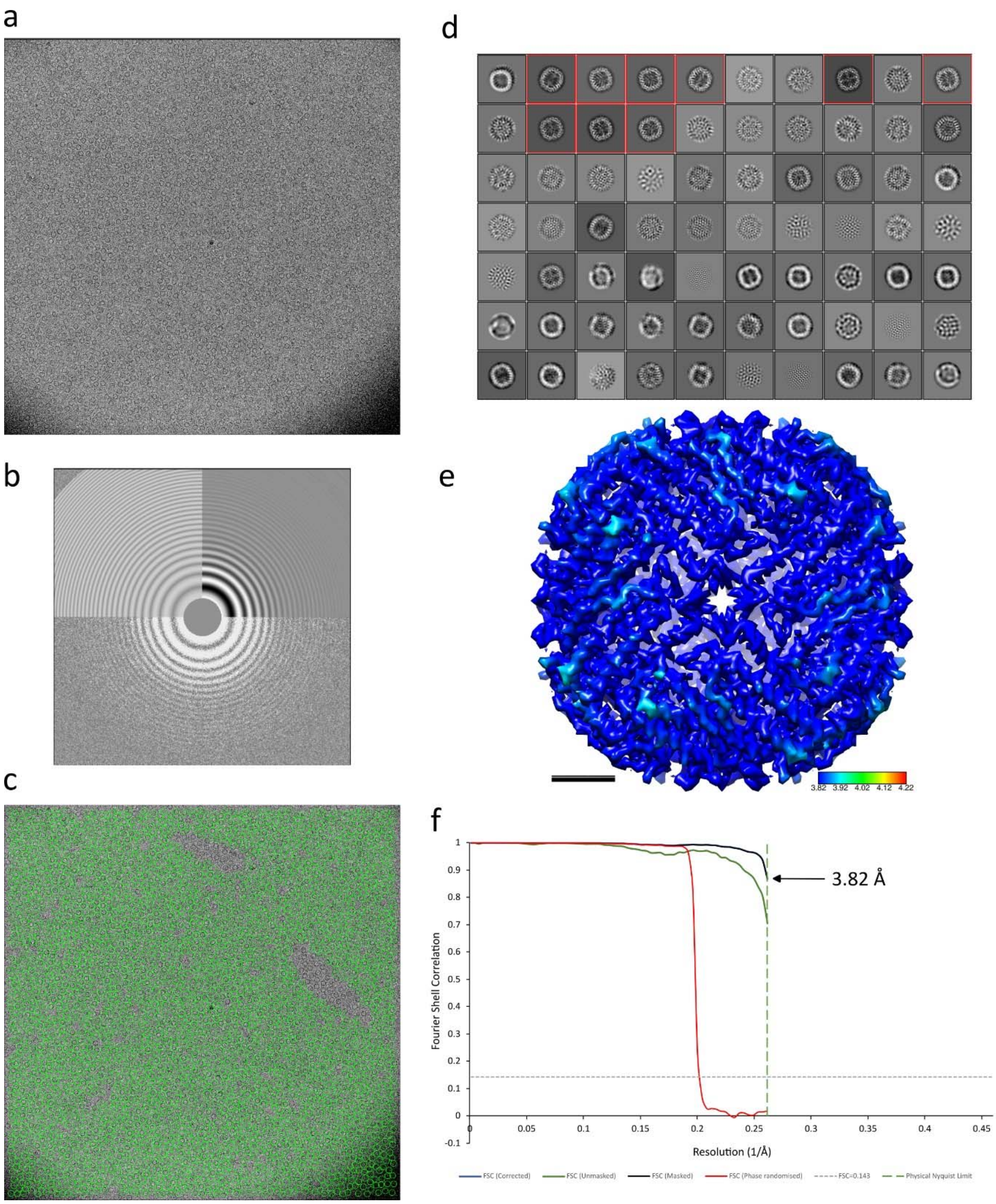

Figure S1 Processing of 4K sampled data from second apoferritin dataset. This dataset permits comparison of behaviour with respect to PASR processing for Falcon 4 detectors. (a) Representative micrograph, (b) power spectrum of (a), (c) template picks from (a) are circled in green, (d) result of 2D classification, and (e) final reconstruction, coloured by local resolution, with the entire reconstruction estimated at the binned Nyquist limit (3.82 Å) from a global gold standard FSC of 3.82 Å, (f) Fourier shell correlation curve (corrected; black line, masked; blue line, unmasked; green line,

phase randomised; red line) with physical Nyquist limit (dashed grey line) and final resolution indicated. Scale bar 2 nm. Density displayed at 5σ.

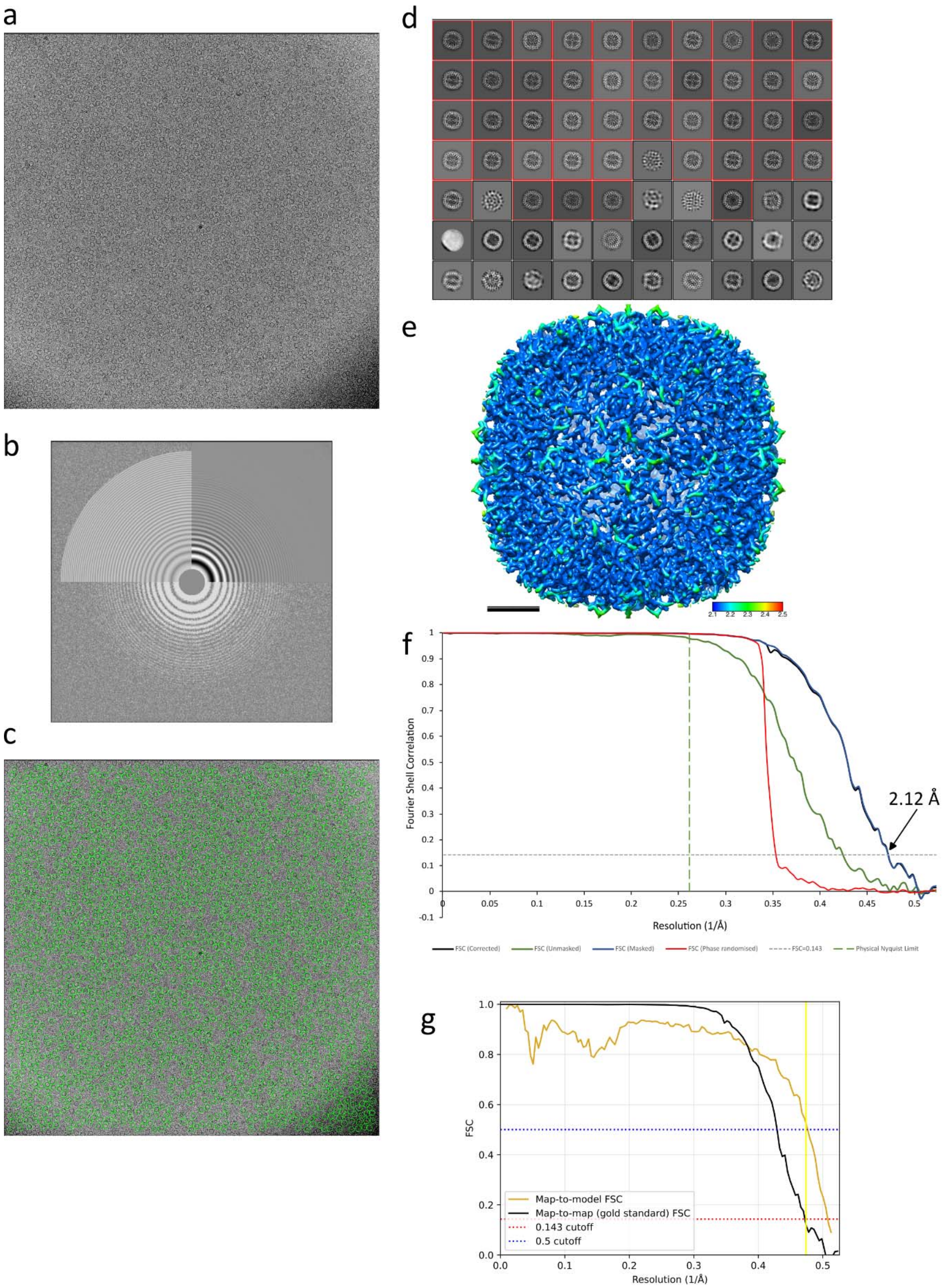

Figure S2 Processing of 8K sampled data from second apoferritin dataset. This dataset permits comparison of behaviour with respect to PASR processing for Falcon 4 detectors. (a) Representative micrograph, (b) power spectrum of (a), (c) template picks from (a) are circled in green, (d) result of 2D classification, and (e) final reconstruction, coloured by local resolution, extending to a local maximum resolution of ~2.1 Å from a global gold standard FSC of 2.13 Å, (f) Fourier shell correlation curve (corrected; black line, masked; blue line, unmasked; green line, phase randomised; red line) with physical Nyquist limit (dashed grey line) and final resolution indicated, (g) map-to-model FSC. Vertical yellow line indicates map-to-map gold-standard FSC resolution (Table S1). Scale bar 2 nm. Density displayed at 5σ.

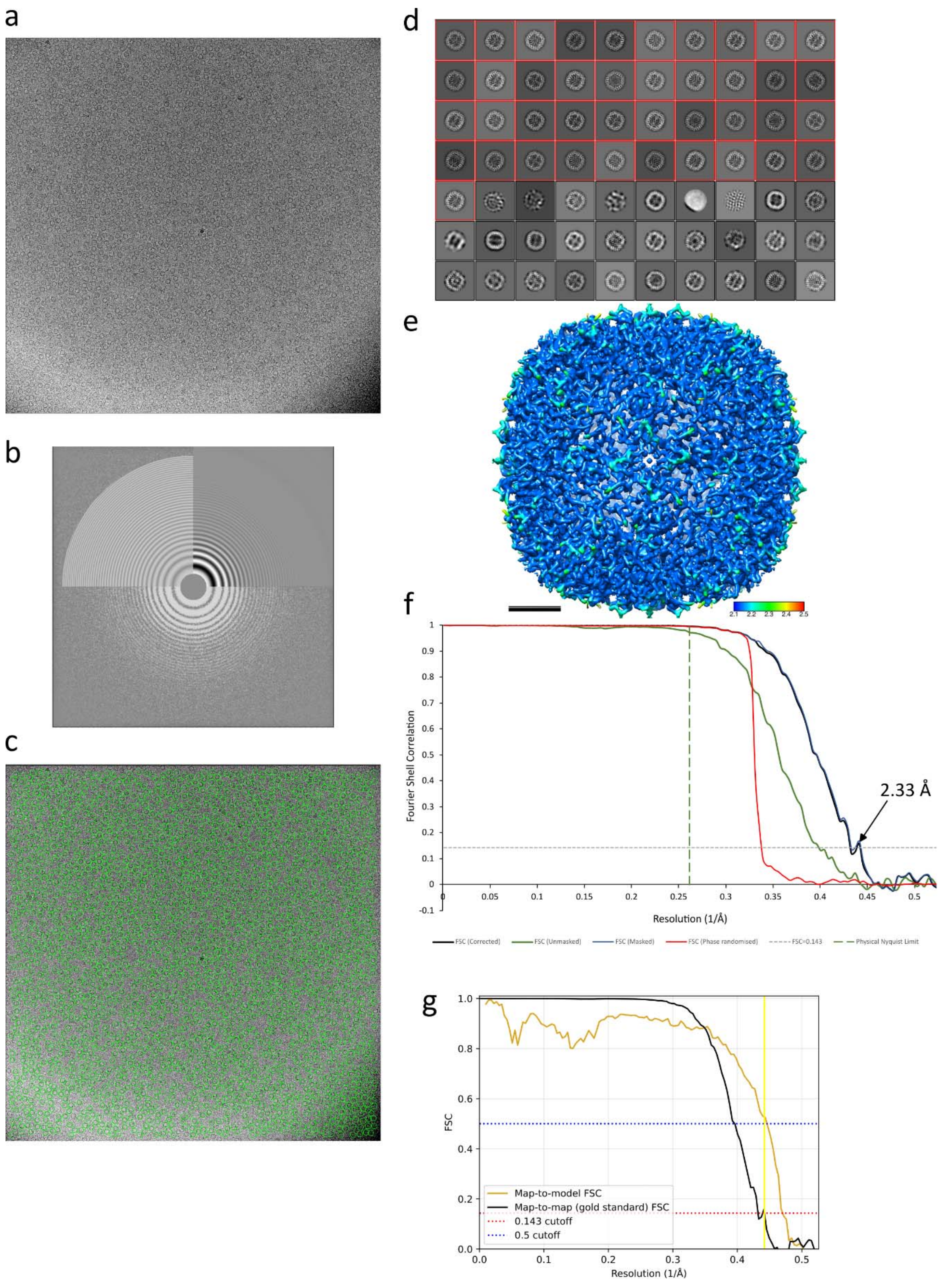

Figure S3 Processing of PASR processed, 4K sampled data from second apoferritin dataset. This dataset permits comparison of behaviour with respect to PASR processing for Falcon 4 detectors. (a) Representative micrograph, (b) power spectrum of (a), (c) template picks from (a) are circled in green, (d) result of 2D classification, and (e) final reconstruction, coloured by local resolution, extending to a

local maximum resolution of ~2.1 Å from a global gold standard FSC of 2.33 Å, (f) Fourier shell correlation curve (corrected; black line, masked; blue line, unmasked; green line, phase randomised; red line) with physical Nyquist limit (dashed grey line) and final resolution indicated, (g) map-to-model FSC. Vertical yellow line indicates map-to-map gold-standard FSC resolution (Table S1). Scale bar 2 nm. Density displayed at 5σ.

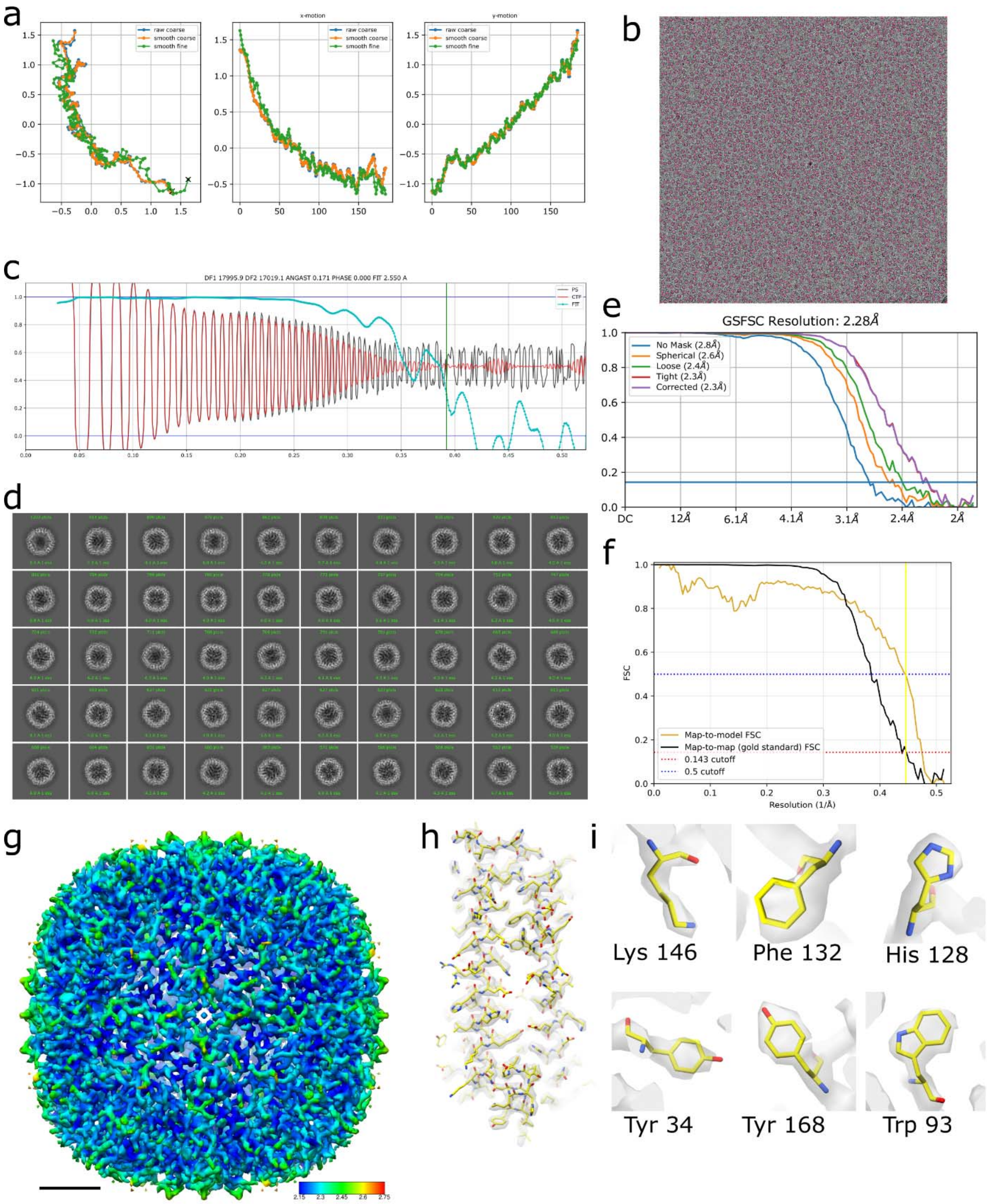

Figure S4 CryoSPARC processing of 8K sampling apoferritin dataset. This dataset permits comparison of behaviour with respect to PASR processing for Falcon 4 detectors in CryoSPARC, rather than RELION. (a) Plots of motion during motion correction, (b) representative micrograph with Gaussian blob picking locations in magenta, (c) 1D diagnostic plot of the estimated CTF, (d) representative 2D classes, (e) gold standard FSC output from homogeneous refinement achieving 2.3 Å, (f) map-to-model FSC. Vertical yellow line indicates map-to-map gold-standard FSC resolution (Table S7), (g) 3D reconstruction coloured by local resolution from 2.15-2.75 Å, scale bar equals 2 nm, (h) segmented apoferritin monomer with fitted PDB model (PDBID:7A6A), and (i) six representative side chain views. Scale bar equals 2nm.

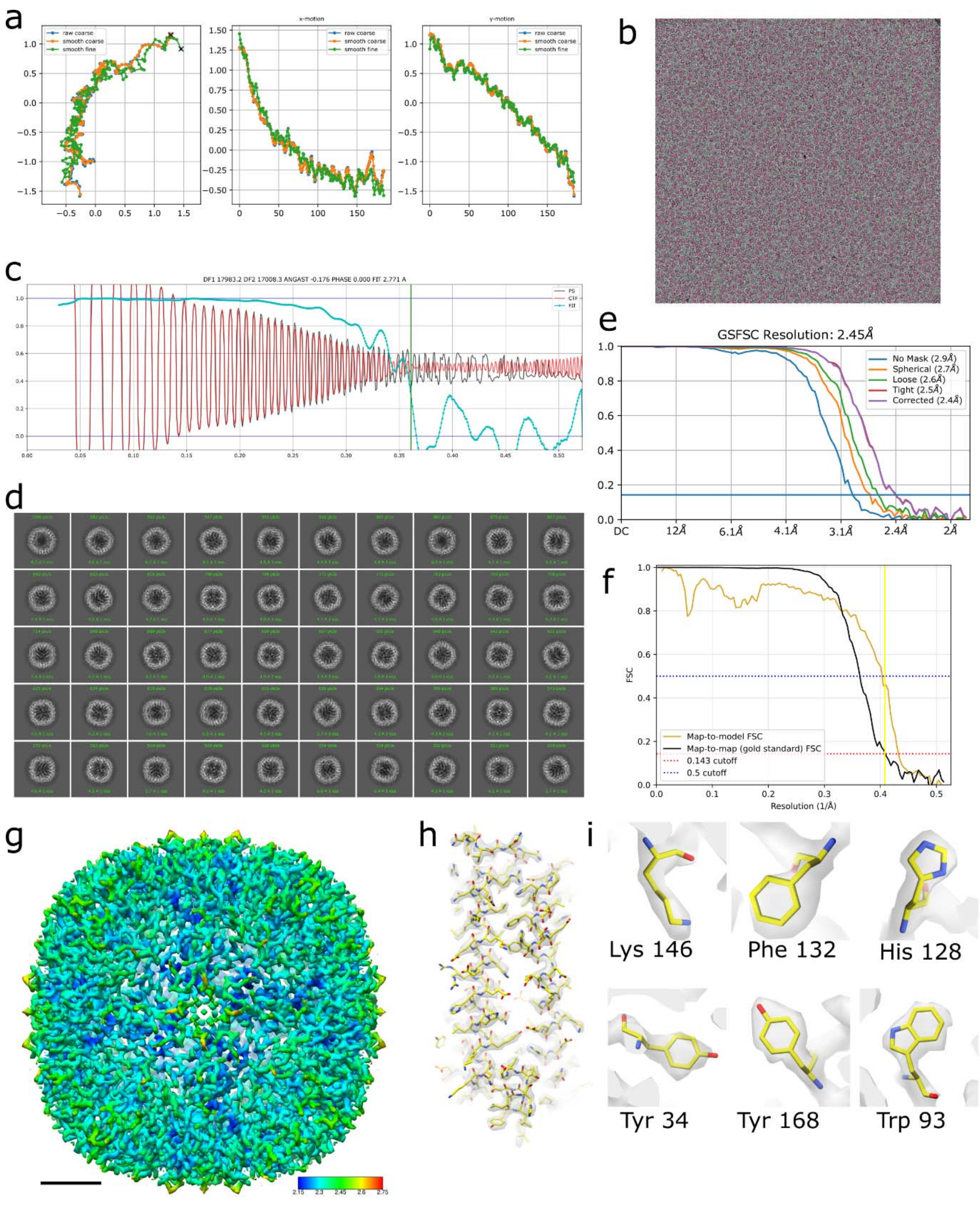

Figure S5 CryoSPARC processing of 4K sampling PASR processed apoferritin dataset. This dataset permits comparison of behaviour with respect to PASR processing for Falcon 4 detectors in CryoSPARC, rather than RELION (a) Plots of motion during motion correction. It should be noted that for speed, the PASR micrographs were loaded as MRC format, which reads in with inverted orientation relative to TIFF so the motion plots are flipped in the x-axis, (b) representative micrograph with Gaussian blob picking locations in magenta, (c) 1D diagnostic plot of the estimated CTF, (d) representative 2D classes, (e) gold standard FSC output from homogeneous refinement achieving 2.45

Å, (f) map-to-model FSC. Vertical yellow line indicates map-to-map gold-standard FSC resolution (Table S7), (g) 3D reconstruction coloured by local resolution from 2.15-2.75 Å, scale bar equals 2 nm, (h) segmented apoferritin monomer with fitted PDB model (PDBID:7A6A), and (i) six representative side chain views. Scale bar equals 2nm.

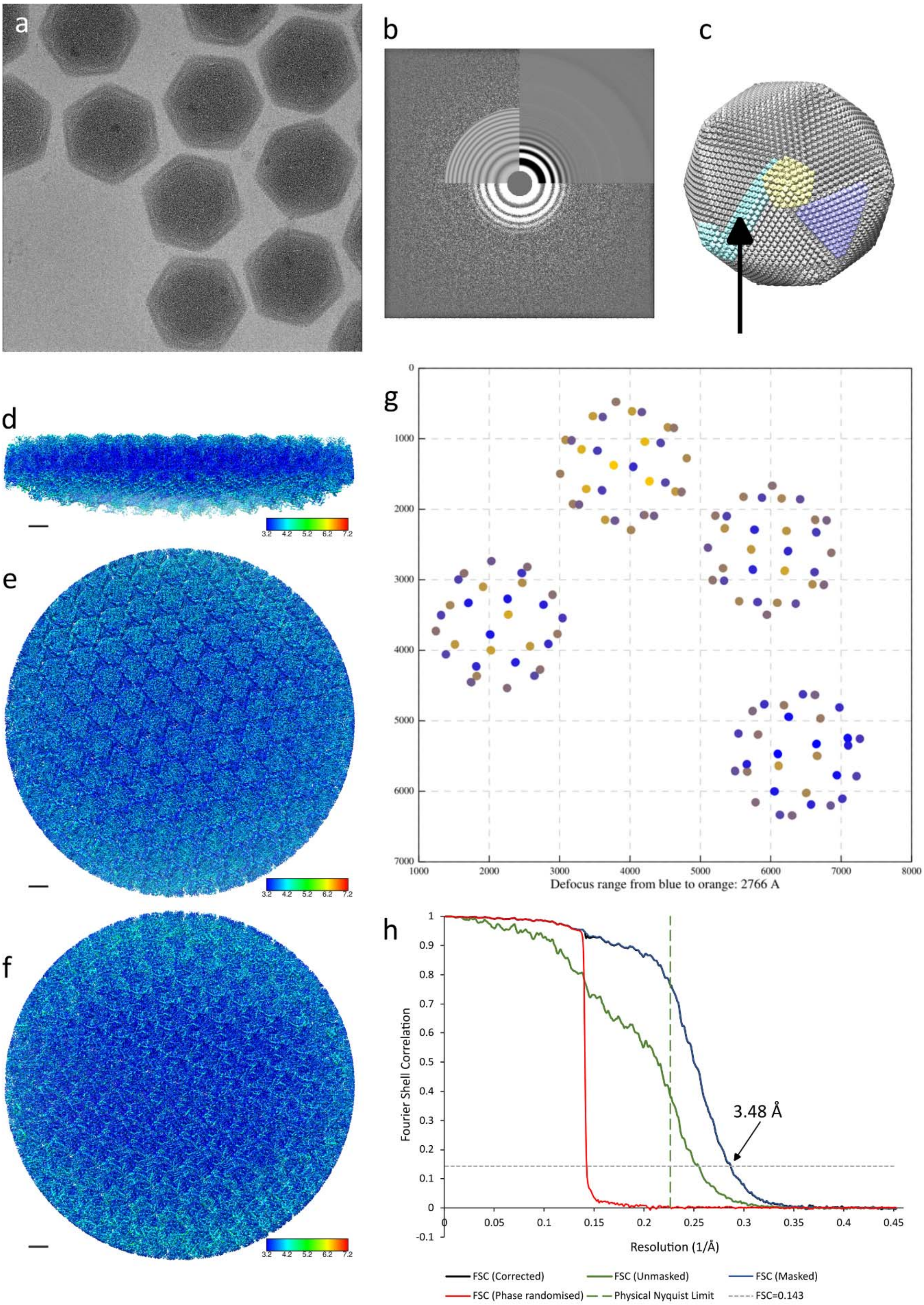

Figure S6 Twofold block-based reconstruction of PASR processed Melbournevirus. This dataset permits comparison of behaviour with respect to PASR processing for Gatan K2 Summit detectors in

counting mode. (a) PASR processed micrograph. (b) Power spectrum and simulated CTF fit from CTFFIND for (a). (c) The whole Melbournevirus with the two, three and fivefold blocks indicated, with an arrow indicating the twofold block. (d) Slice through the centre of the twofold block, showing internal estimated local resolution. (e) view of the outside of the twofold block, coloured by local resolution (f) view of the inside of the twofold block, coloured by local resolution, (g) points in one of the micrographs showing the locations of the twofold block sections, with their local defocus. (h) Gold standard Fourier shell correlation of the twofold PASR processed Melbournevirus block, indicating the corrected FSC (black curve), masked FSC (blue curve), unmasked FSC (green curve), phase randomised FSC (red curve), FSC 0.143 metric (dashed grey line) and Nyquist limit from the original data (long dashed green line). Scale bar 5 nm. Density displayed at 3σ.

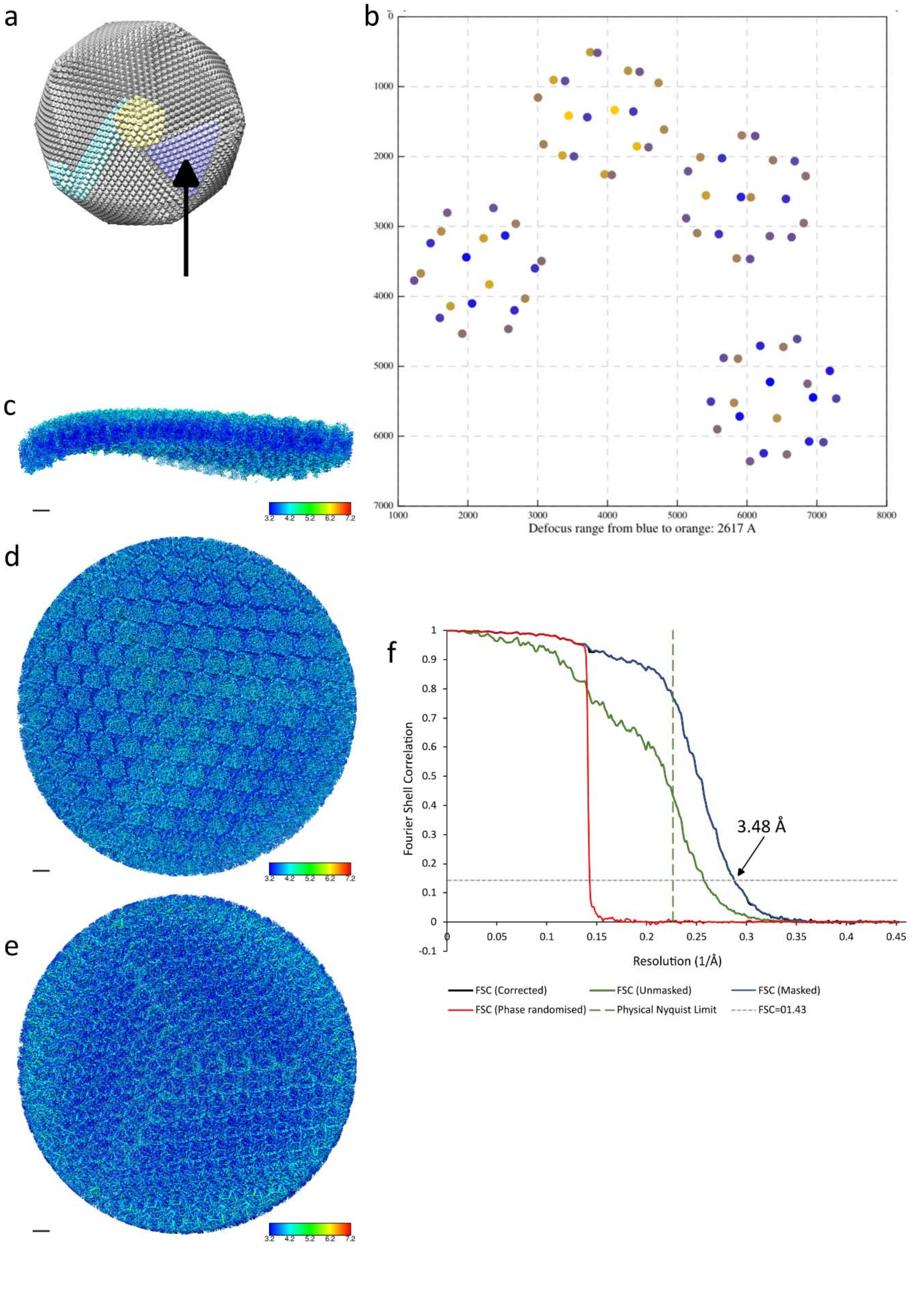

Figure S7 Threefold block-based reconstruction of PASR processed Melbournevirus. This dataset permits comparison of behaviour with respect to PASR processing for Gatan K2 Summit detectors in

counting mode. (a) The whole Melbournevirus with the two, three and fivefold blocks indicated, with an arrow indicating the threefold block. (b) Points in one of the micrographs showing the locations of the threefold block sections, with their local defocus. (c) Slice through the centre of the threefold block, showing internal estimated local resolution. (d) View of the outside of the threefold block, coloured by local resolution (e) view of the inside of the threefold block, coloured by local resolution. (f) Gold standard Fourier shell correlation of the threefold PASR processed Melbournevirus block, indicating the corrected FSC (black curve), masked FSC (blue curve), unmasked FSC (green curve), phase randomised FSC (red curve), FSC 0.143 metric (dashed grey line) and Nyquist limit from the original data (long dashed green line). Scale bar 5 nm. Density displayed at 3σ.

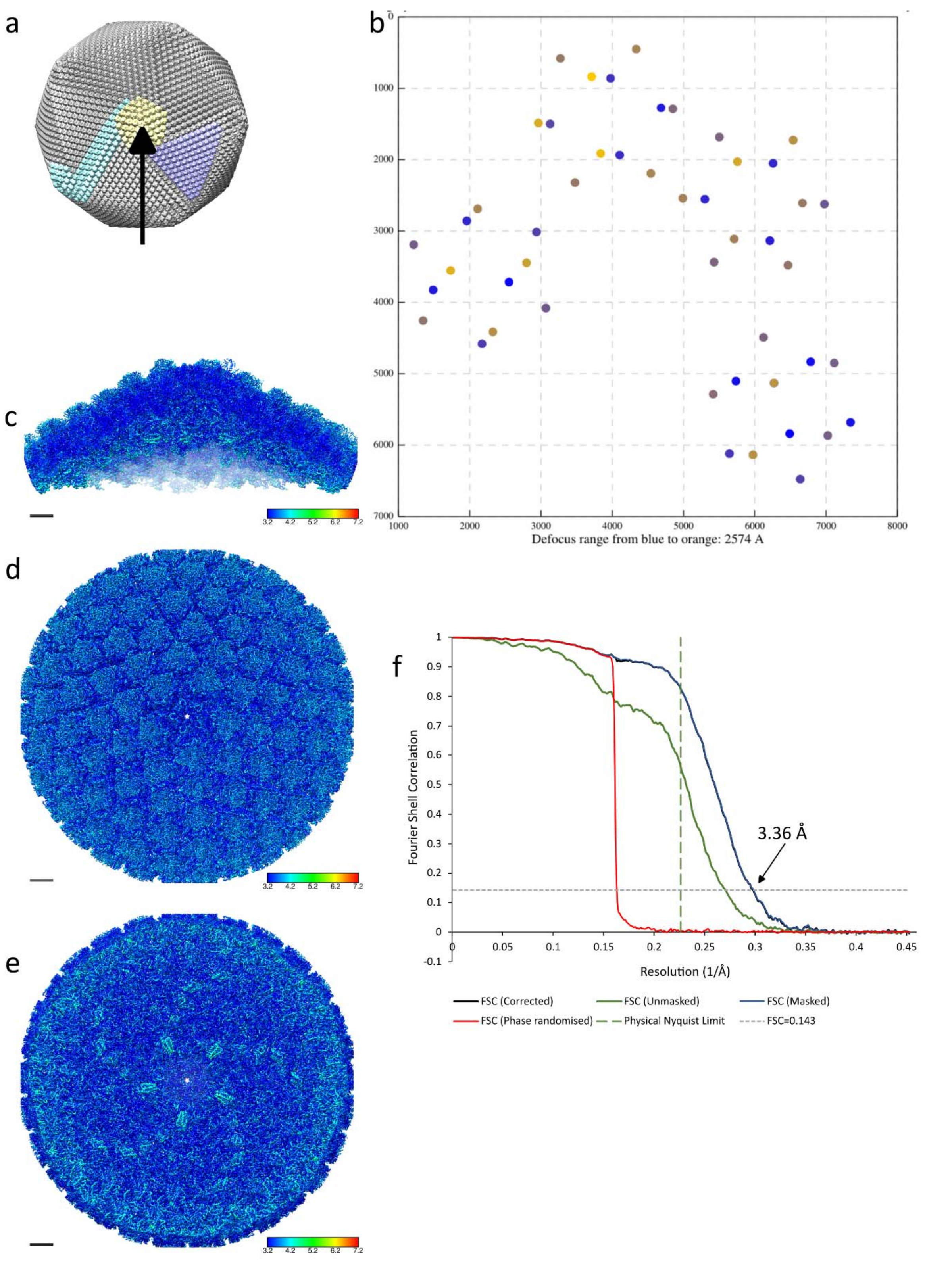

Figure S8 Fivefold block-based reconstruction of PASR processed Melbournevirus. This dataset permits comparison of behaviour with respect to PASR processing for Gatan K2 Summit detectors in

counting mode. (a) The whole Melbournevirus with the two, three and fivefold blocks indicated, with an arrow indicating the fivefold block. (b) Points in one of the micrographs showing the locations of the fivefold block sections, with their local defocus. (c) Slice through the centre of the fivefold block, showing internal estimated local resolution. (d) View of the outside of the fivefold block, coloured by local resolution (e) view of the inside of the fivefold block, coloured by local resolution. (f) Gold standard Fourier shell correlation of the threefold PASR processed Melbournevirus block, indicating the corrected FSC (black curve), masked FSC (blue curve), unmasked FSC (green curve), phase randomised FSC (red curve), FSC 0.143 metric (dashed grey line) and Nyquist limit from the original data (long dashed green line). Scale bar 5 nm. Density displayed at 3σ.

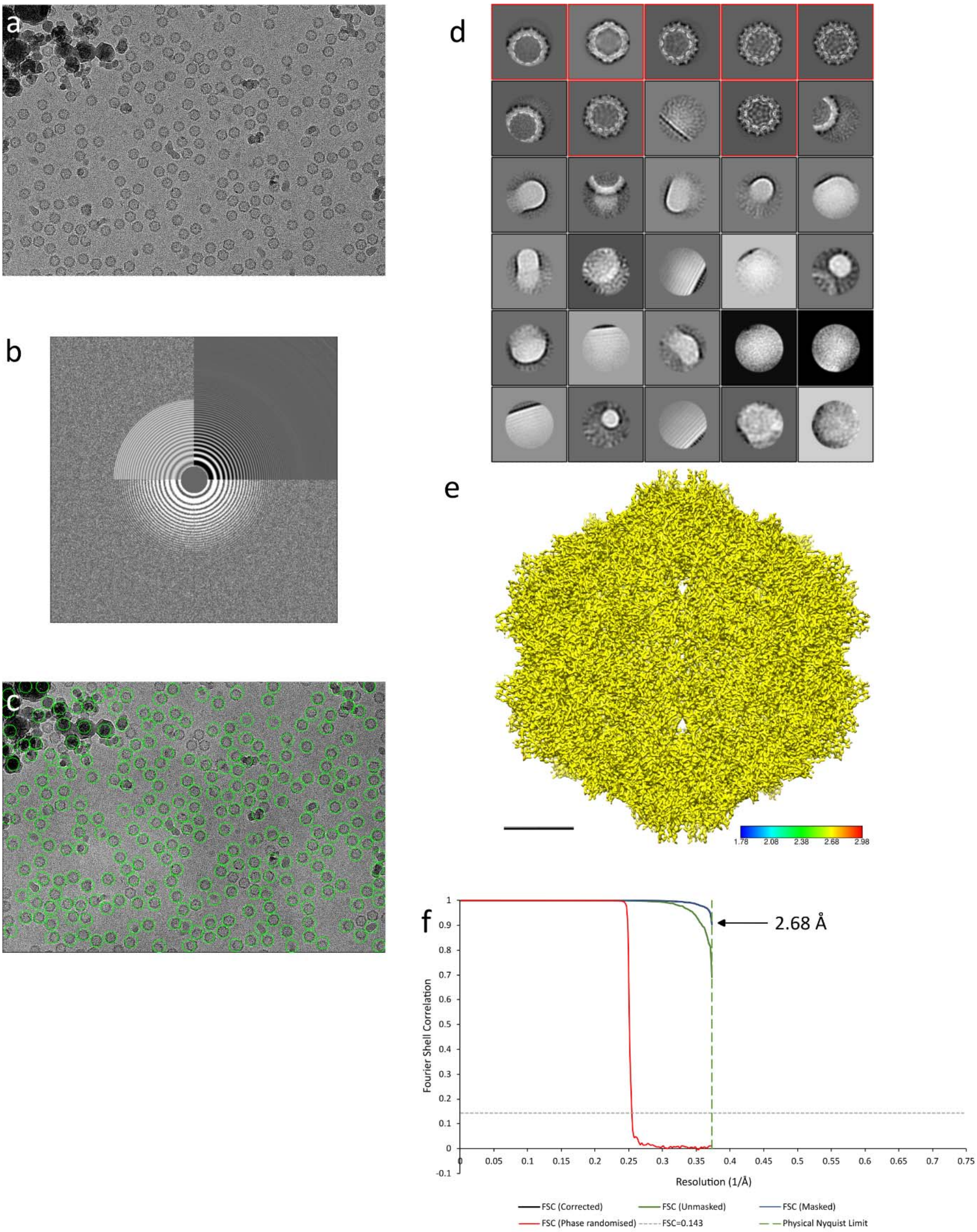

Figure S9 Processing of the original adeno-associated virus dataset. This dataset permits comparison of behaviour with respect to PASR processing for Gatan K3 detectors in correlative double sampling (CDS) mode. (a) Representative micrograph, (b) power spectrum showing good fit to Thon rings for (a), (c) LoG autopicking of (a), (d) representative 2D classes, with selected classes highlighted in red, (e) final reconstruction, coloured by local resolution at 2.68 Å, scale bar equals 5

nm, and (f) Fourier shell correlation curve (corrected; black line, masked; blue line, unmasked; green line, phase randomised; red line) with Nyquist limit (dashed grey line) and final resolution indicated.

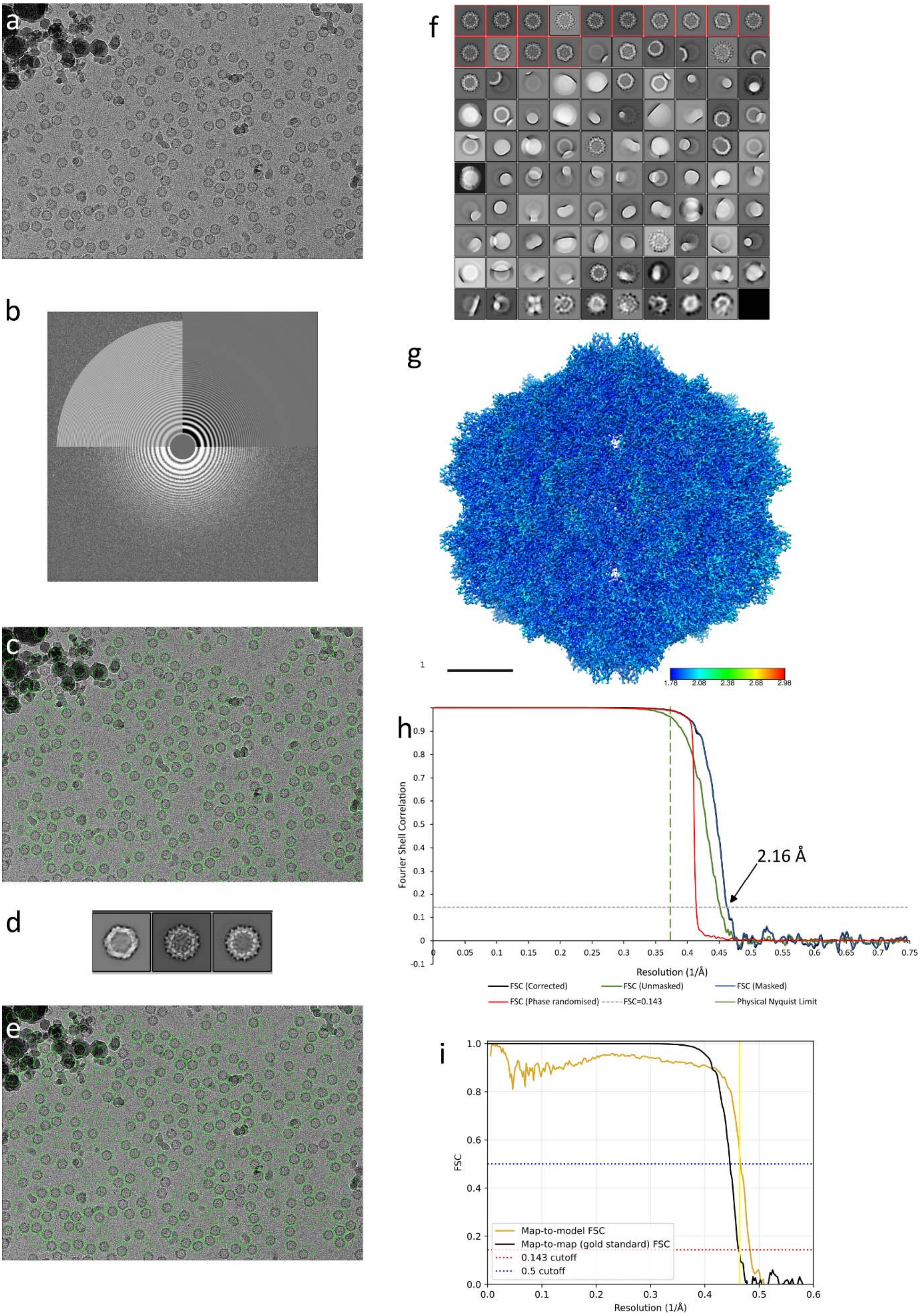

Figure S10 Processing of the PASR processed adeno-associated virus dataset. This dataset permits comparison of behaviour with respect to PASR processing for Gatan K3 detectors in correlative double sampling (CDS) mode. (a) Representative micrograph, (b) power spectrum showing good fit to Thon rings for (a), (c) LoG autopicking of (a), (d) representative 2D classes, which were used for template autopicking, (e) micrograph with template autopicks circled in green, (f) 2D classes, with selected classes highlighted in red, and (g) final reconstruction, coloured by local resolution, extending to ~1.78 Å. Scale bar equals 5 nm. (h) Fourier shell correlation curve (corrected; black line, masked; blue line, unmasked; green line, phase randomised; red line) with Nyquist limit (dashed grey line) and final resolution indicated, (g) map-to-model FSC. Vertical yellow line indicates map-to-map gold-standard FSC resolution (Table S5).

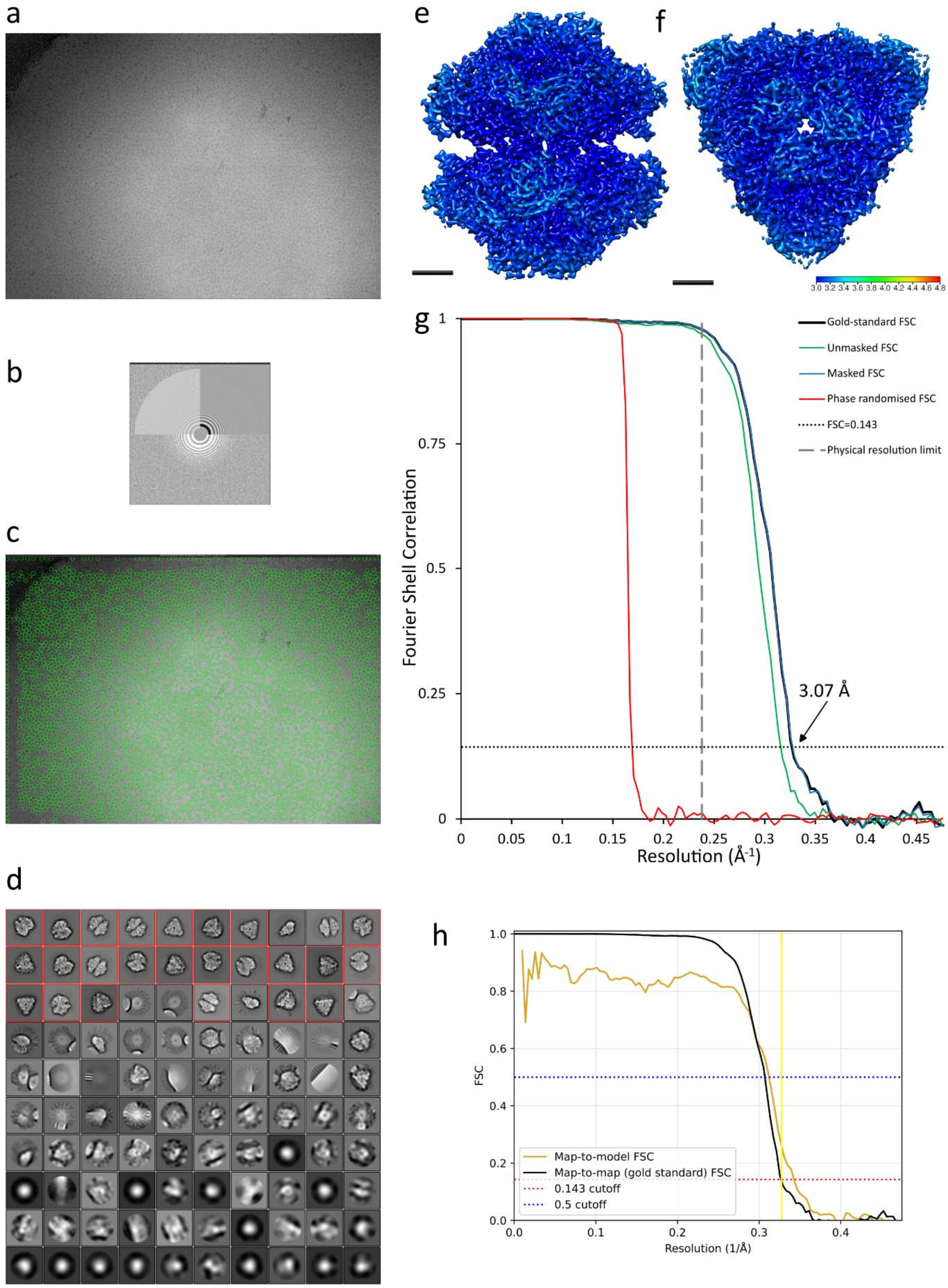

Figure S11 Processing of jack bean urease (EMPIAR-10549). This dataset permits comparison of behaviour with respect to PASR processing for Gatan K3 detectors in super resolution mode, and for 200 kV acceleration voltage microscopes. (a) A representative micrograph, (b) power spectrum of (a), (c) LoG autopicking of (a), note that the LoG autopicking algorithm “mis-picks” at the edges, (d) 2D

classes, with selected classes highlighted in red, (e) side view of jack bean urease reconstruction, coloured by local resolution, with resolution extending to ~3 Å, and (f) top view of (e). (g) RELION gold standard FSC (corrected; black line, masked; blue line, unmasked; green line, phase randomised; red line) showing the data exceeding the physical Nyquist limit, (h) map-to-model FSC. Vertical yellow line indicates map-to-map gold-standard FSC resolution (Table S3). Scale bar equals 2 nm.

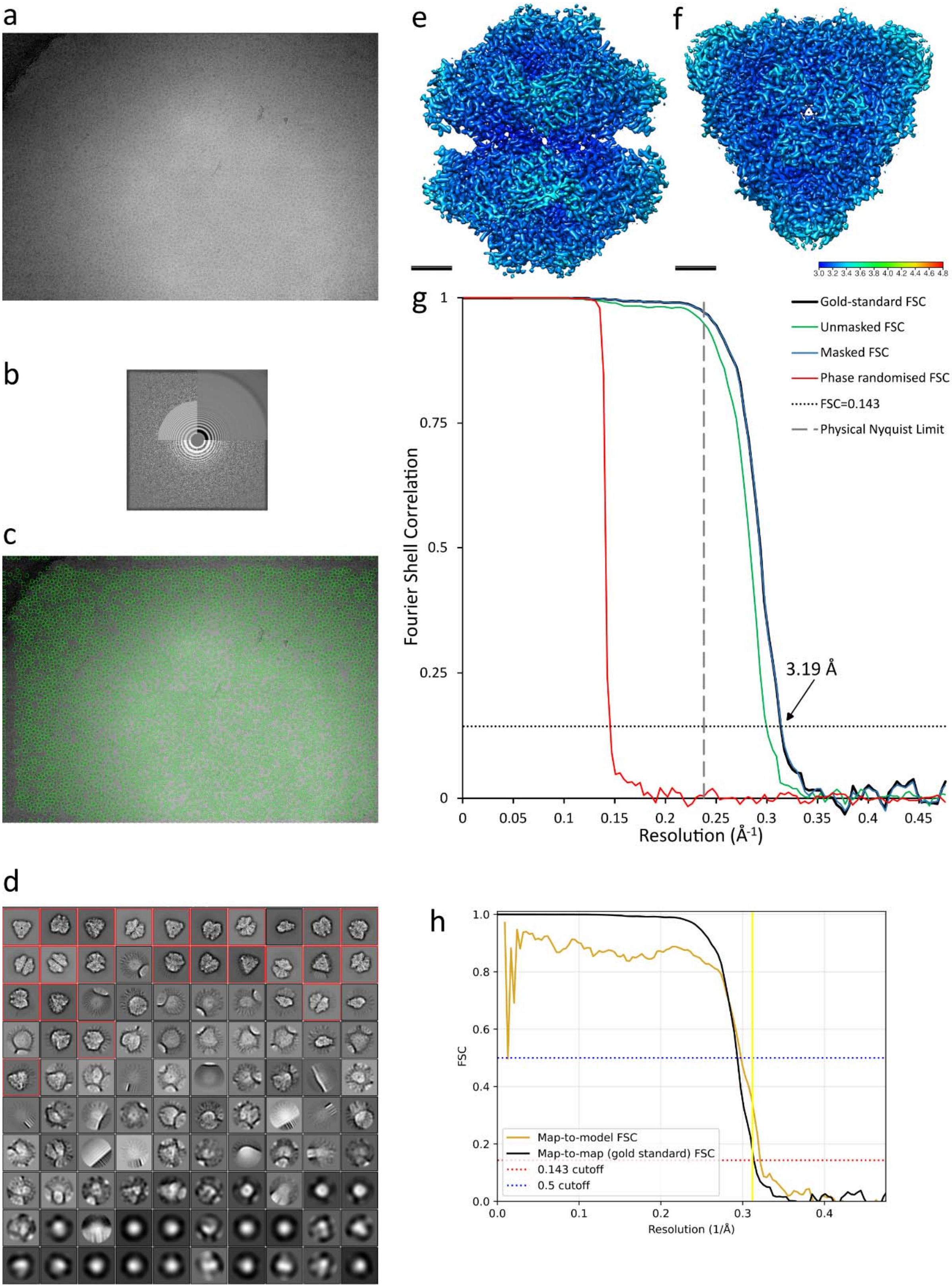

Figure S12 Processing of jack bean urease (EMPIAR-10549) after PASR processing. This dataset permits comparison of behaviour with respect to PASR processing for Gatan K3 detectors in super resolution mode, and for 200 kV acceleration voltage microscopes. (a) a representative micrograph, (b) power spectrum of (a), (c) LoG autopicking of (a), note that the LoG autopicking algorithm “mis-

picks” at the edges, (d) 2D classes, with selected classes highlighted in red, (e) side view of jack bean urease reconstruction, coloured by local resolution, with resolution extending to ~3 Å, and (f) top view of (e). (g) RELION gold standard FSC (corrected; black line, masked; blue line, unmasked; green line, phase randomised; red line) showing the data exceeding the physical Nyquist limit, (h) map-to-model FSC. Vertical yellow line indicates map-to-map gold-standard FSC resolution (Table S3). Scale bar equals 2 nm.

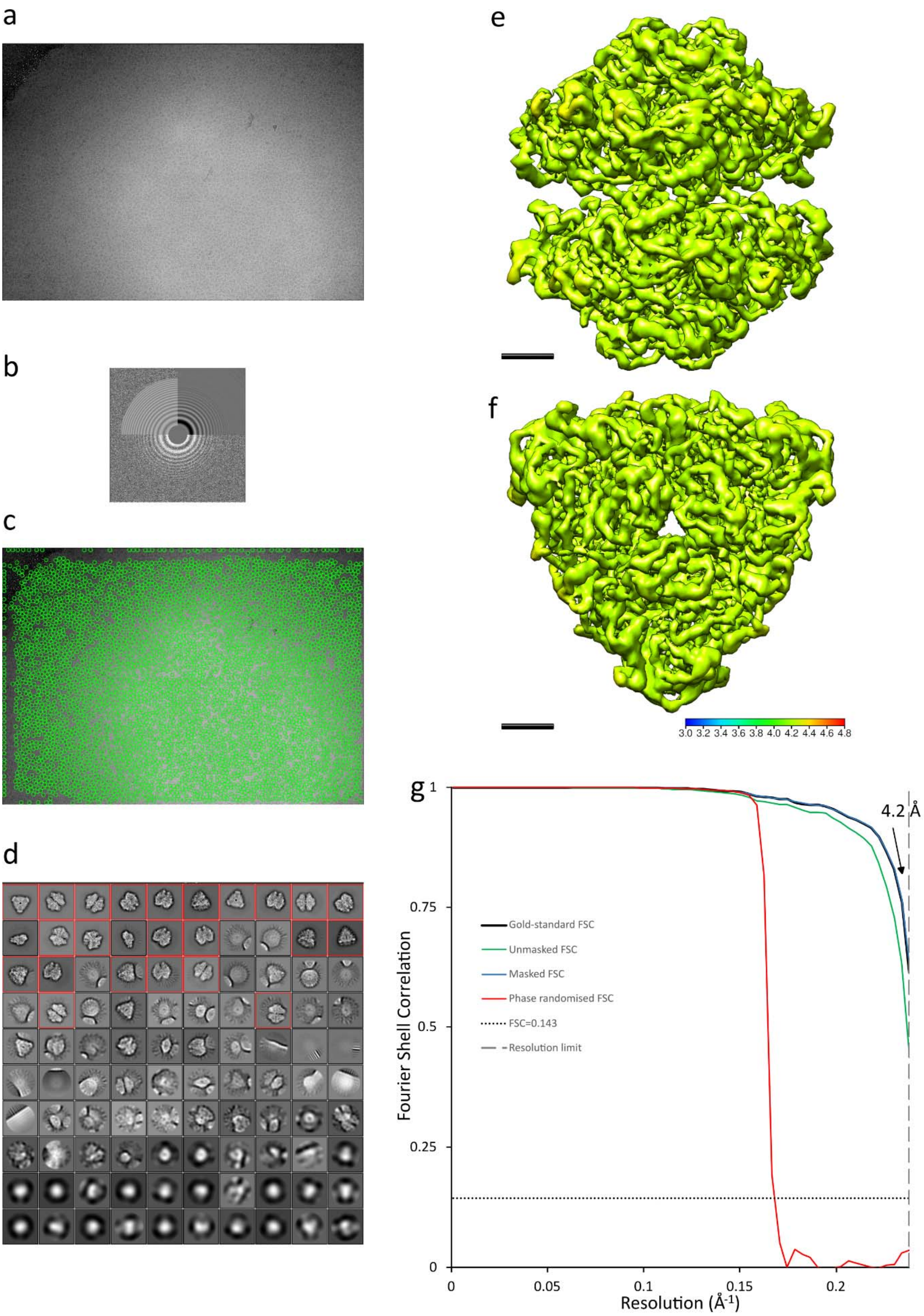

Figure S13 Processing of jack bean urease (EMPIAR-10549) after binning micrographs to physical Nyquist. This dataset permits comparison of behaviour with respect to PASR processing for Gatan K3

detectors in super resolution mode, and for 200 kV acceleration voltage microscopes. (a) A representative micrograph, (b) power spectrum of (a), (c) LoG autopicking of (a), note that the LoG autopicking algorithm “mis-picks” at the edges, (d) 2D classes, with selected classes highlighted in red, (e) side view of jack bean urease reconstruction, coloured by local resolution (structure is globally 4.2 Å), and (f) top view of (e). (g) RELION gold standard FSC (corrected; black line, masked; blue line, unmasked; green line, phase randomised; red line) showing the data reaching the physical Nyquist limit. Scale bar equals 2 nm.

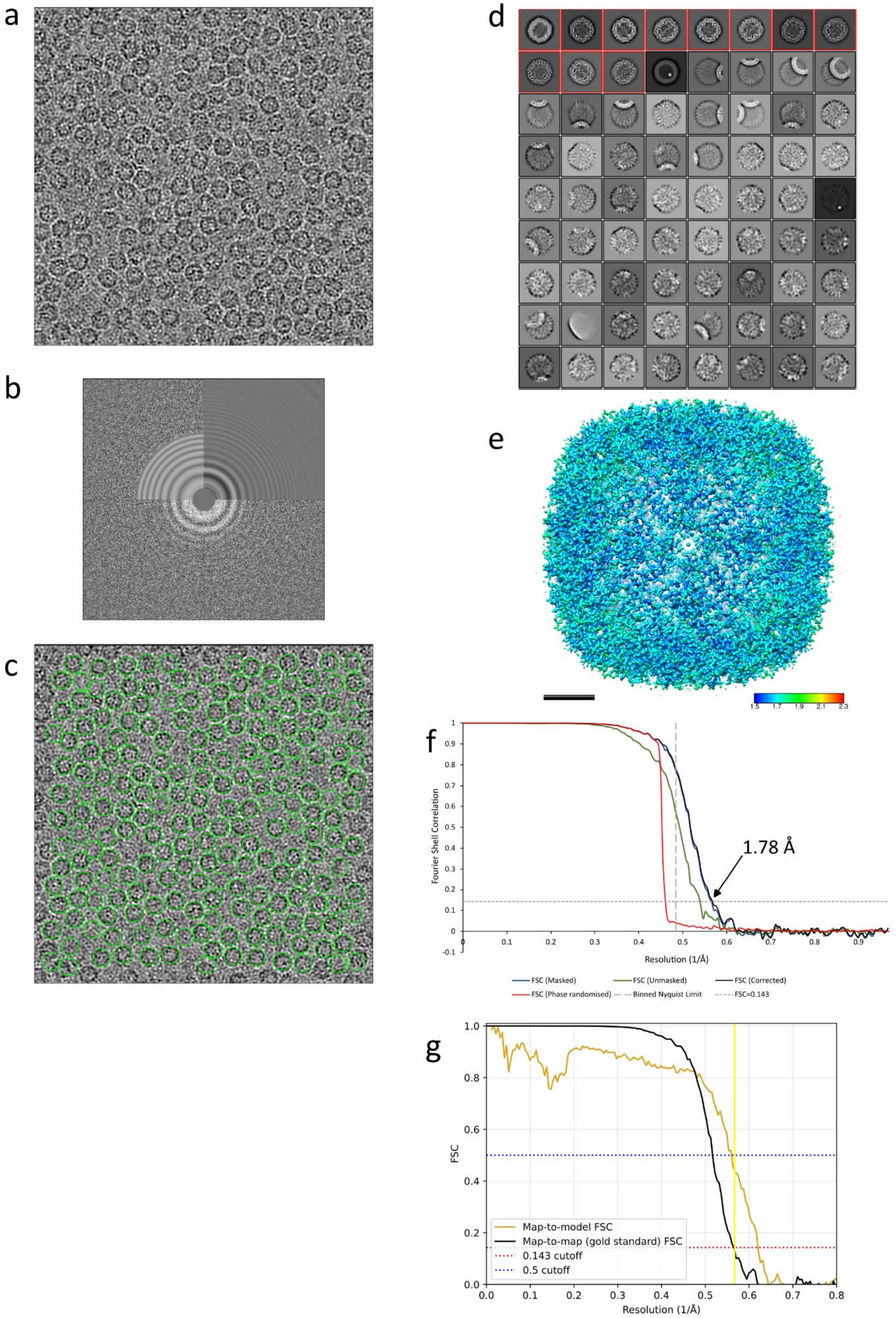
a
b
c
d
e
1.5 1.7 1.9 2.1 2.3
f
Fourier Shell Correlation
Resolution (1/Å)
1.78 Å
FSC (Masked)
FSC (Unmasked)
FSC (Corrected)
FSC (Phase randomised)
Binned Nyquist Limit
FSC=0.143
g
FSC
Resolution (1/Å)
Map-to-model FSC
Map-to-map (gold standard) FSC
0.143 cutoff
0.5 cutoff

Figure S14 Application of PASR pre-motion correction (i.e., to movies) on EMPIAR-10216. This dataset permits comparison of behaviour with respect to PASR processing for Falcon 3 detectors, which do not have a super resolution mode. (a) Representative micrograph, (b) power spectrum of (a), (c) template picks from (a) are circled in green, (d) result of 2D classification, and (e) final reconstruction, coloured by local resolution, extending to a local maximum resolution of ~1.5 Å from (f) a global gold standard FSC (corrected; black line, masked; blue line, unmasked; green line, phase randomised; red line) of 1.78 Å, (g) map-to-model FSC. Vertical yellow line indicates map-to-map gold-standard FSC resolution (Table S2). Scale bar 2 nm. Density displayed at 5σ.

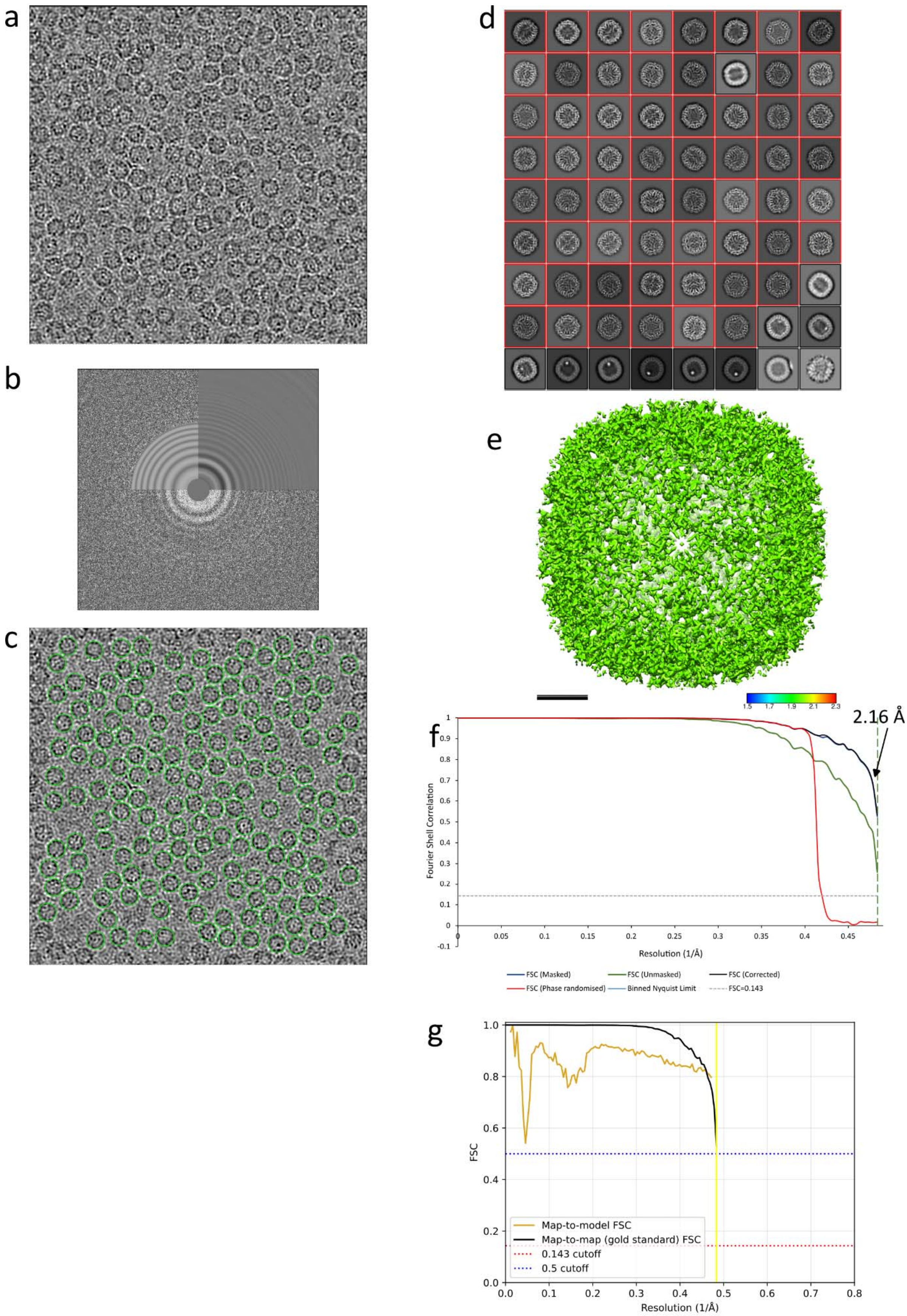
a
b
c
d
e
1.5 1.7 1.9 2.1 2.3
f
2.16 Å
Fourier Shell Correlation
Resolution (1/Å)
FSC (Masked)
FSC (Unmasked)
FSC (Corrected)
FSC (Phase randomised)
Binned Nyquist Limit
FSC=0.143
g
FSC
Resolution (1/Å)
Map-to-model FSC
Map-to-map (gold standard) FSC
0.143 cutoff
0.5 cutoff

Figure S15 Binned processing of EMPIAR-10216. This dataset permits comparison of behaviour with respect to PASR processing for Falcon 3 detectors, which do not have a super resolution mode. (a) Representative micrograph, (b) power spectrum of (a), (c) template picks from (a) are circled in green, (d) result of 2D classification, and (e) final reconstruction, coloured by local resolution, with the entire reconstruction estimated at the binned Nyquist limit (2.068 Å) from (f) a global gold standard FSC (corrected; black line, masked; blue line, unmasked; green line, phase randomised; red line) of 2.068 Å, (g) map-to-model FSC. Vertical yellow line indicates map-to-map gold-standard FSC resolution (Table S2). Scale bar 2 nm. Density displayed at 5σ.

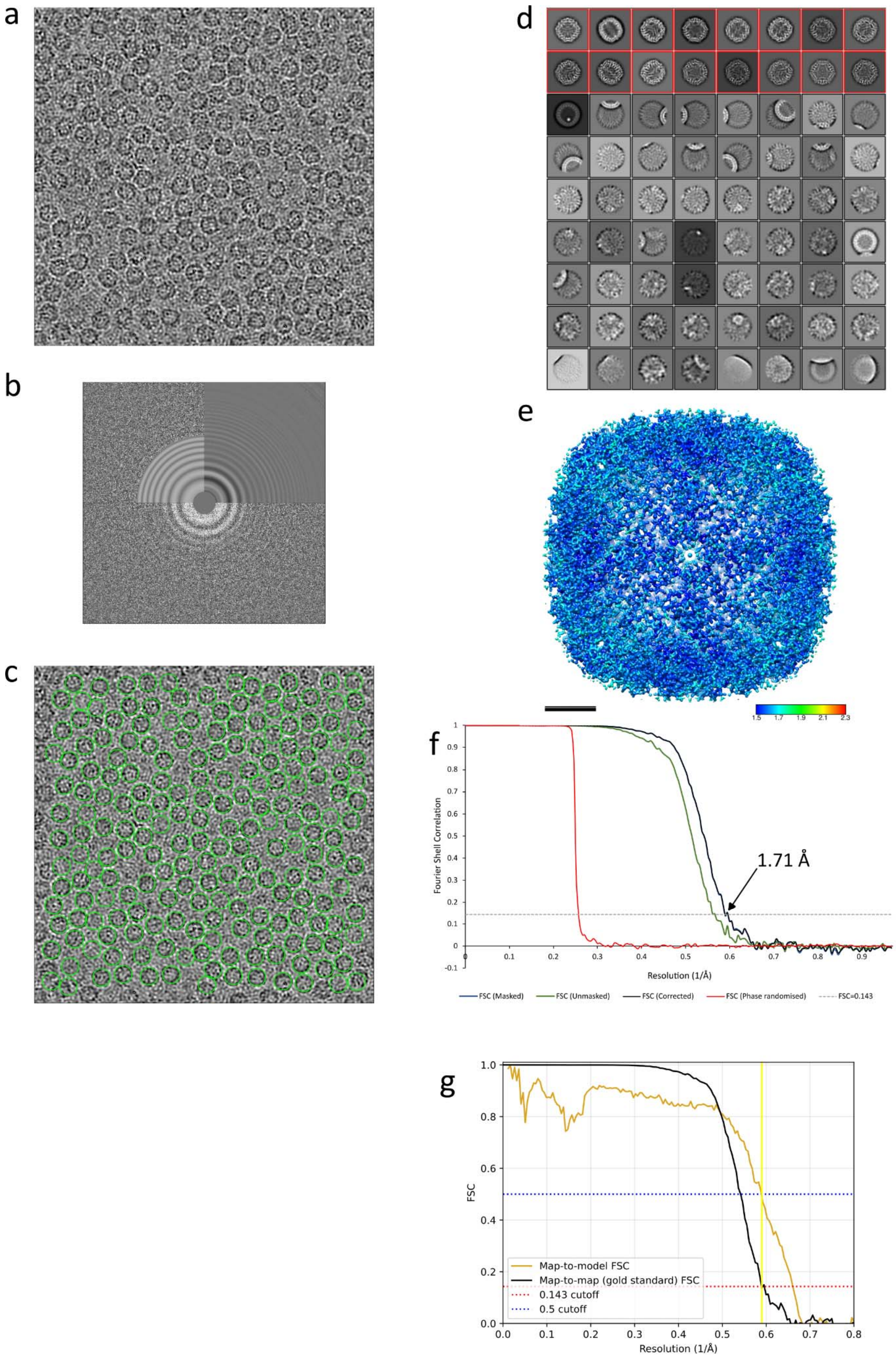
a
b
c
d
e
1.5
1.7
1.9
2.1
2.3
f
1.71 Å
Fourier Shell Correlation
Resolution (1/Å)
FSC (Masked)
FSC (Unmasked)
FSC (Corrected)
FSC (Phase randomised)
FSC=0.143
g
FSC
Resolution (1/Å)
Map-to-model FSC
Map-to-map (gold standard) FSC
0.143 cutoff
0.5 cutoff

Figure S16 Reprocessing of the original EMPIAR-10216 dataset. This dataset permits comparison of behaviour with respect to PASR processing for Falcon 3 detectors, which do not have a super resolution mode. (a) Representative micrograph, (b) power spectrum of (a), (c) template picks from (a) are circled in green, (d) result of 2D classification, and (e) final reconstruction, coloured by local resolution, extending to a local maximum resolution of ~1.5 Å from (f) a global gold standard FSC (corrected; black line, masked; blue line, unmasked; green line, phase randomised; red line) of 1.71 Å, (g) map-to-model FSC. Vertical yellow line indicates map-to-map gold-standard FSC resolution (Table S2). Scale bar 2 nm. Density displayed at 5σ.

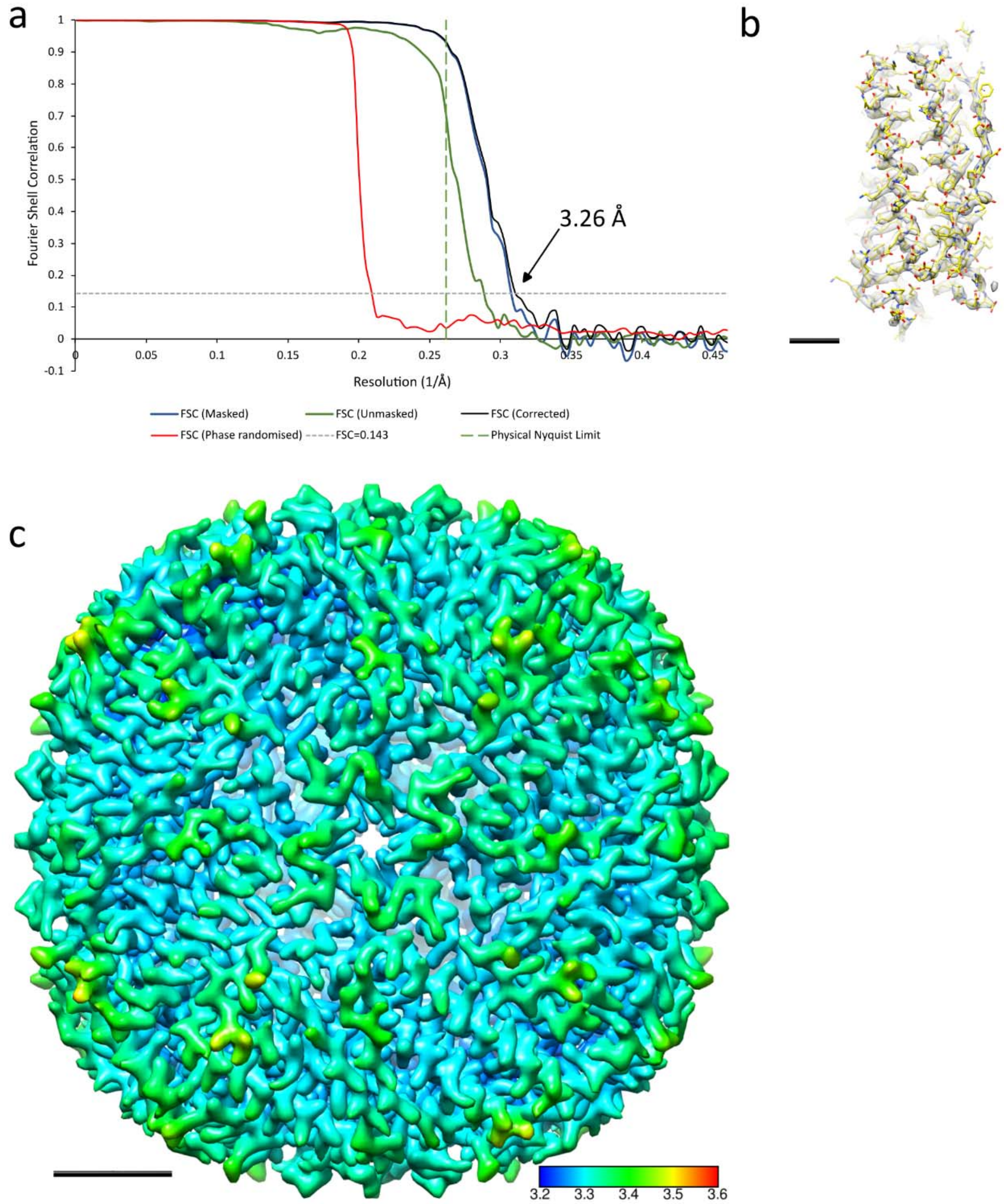

Figure S17 Application of PASR post-motion correction (i.e., to single frame micrographs) on the second apoferritin (64,000×) dataset. This dataset permits comparison of behaviour with respect to PASR processing for single frame micrograph data for smaller particles. (a) RELION gold standard FSC (corrected; black line, masked; blue line, unmasked; green line, phase randomised; red line). The corrected curve falls to 3.26 Å at FSC = 0.143. (b) Apoferritin monomer, with fitted PDB showing what would be expected at ~3.3 Å; side chain density but lacking clear features. (c) Post-motion correction PASR reconstruction with resolution only extending to ~3.2 Å, rather than the ~2.1 Å if applying PASR prior to motion correction. Scale bar for (b) equals 1 nm, for (c) equals 2 nm.

a

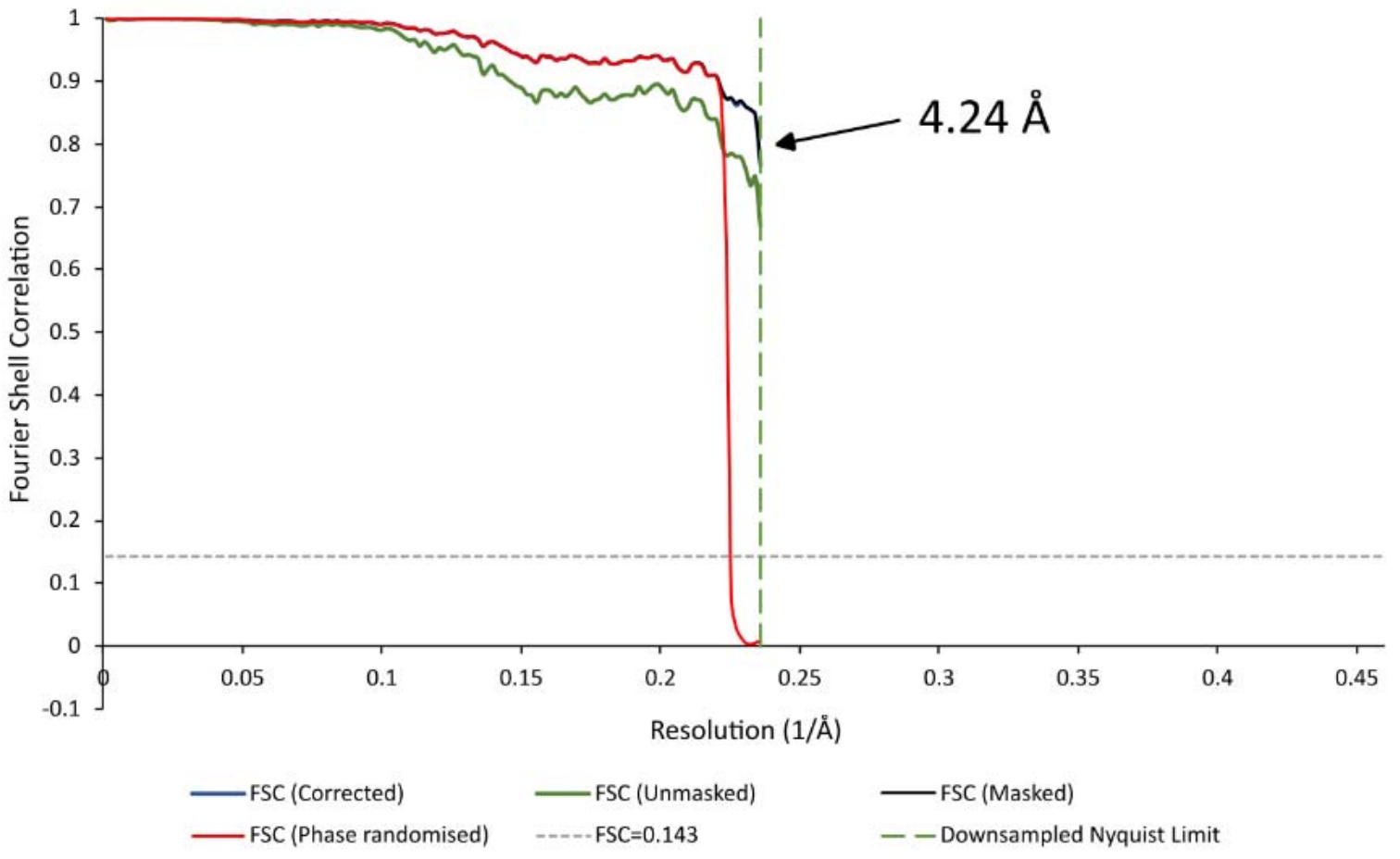
4.24 Å
Fourier Shell Correlation
Resolution (1/Å)
FSC (Corrected)
FSC (Unmasked)
FSC (Masked)
FSC (Phase randomised)
FSC=0.143
Downsampled Nyquist Limit

b

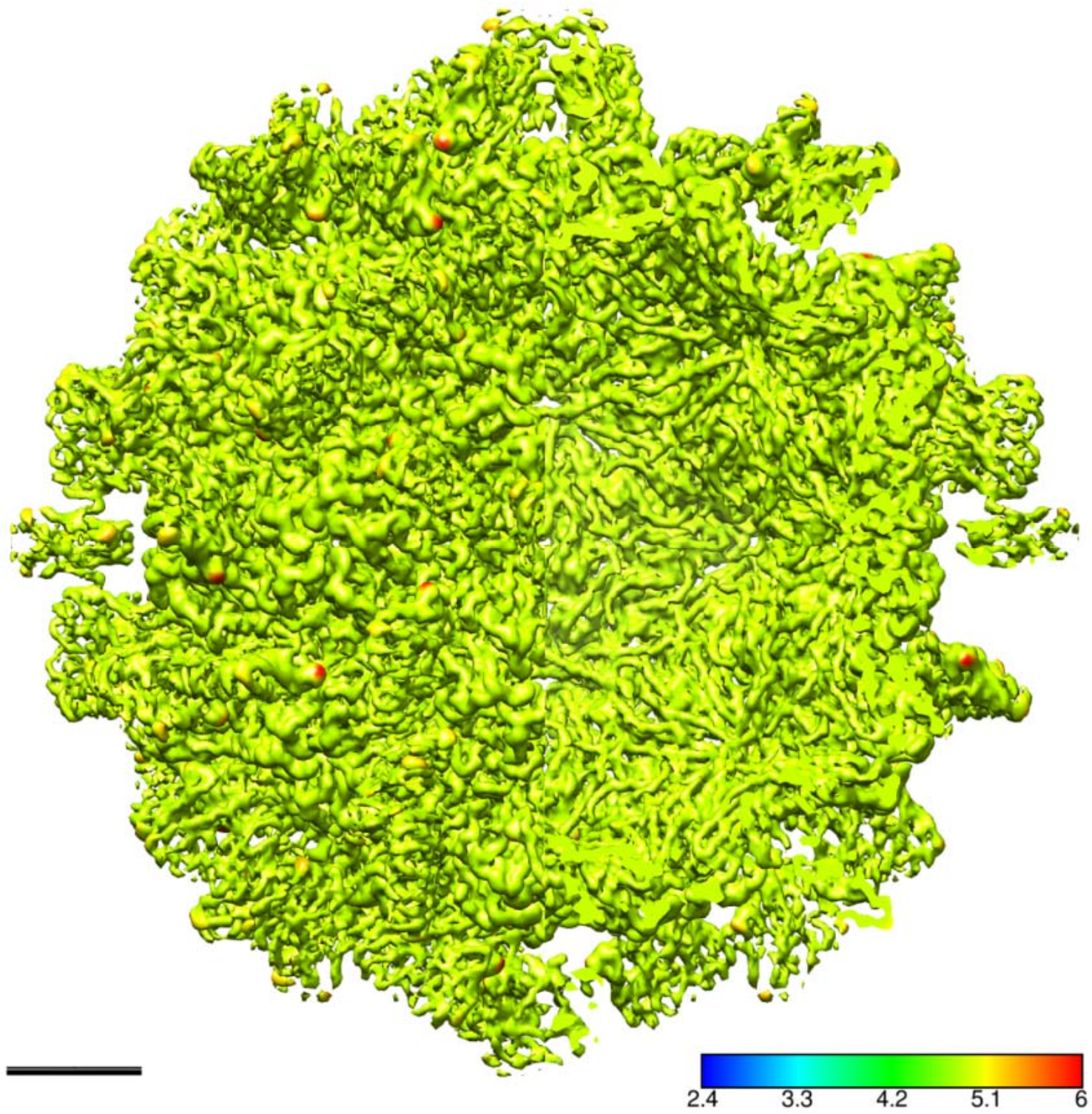
2.4
3.3
4.2
5.1
6

c

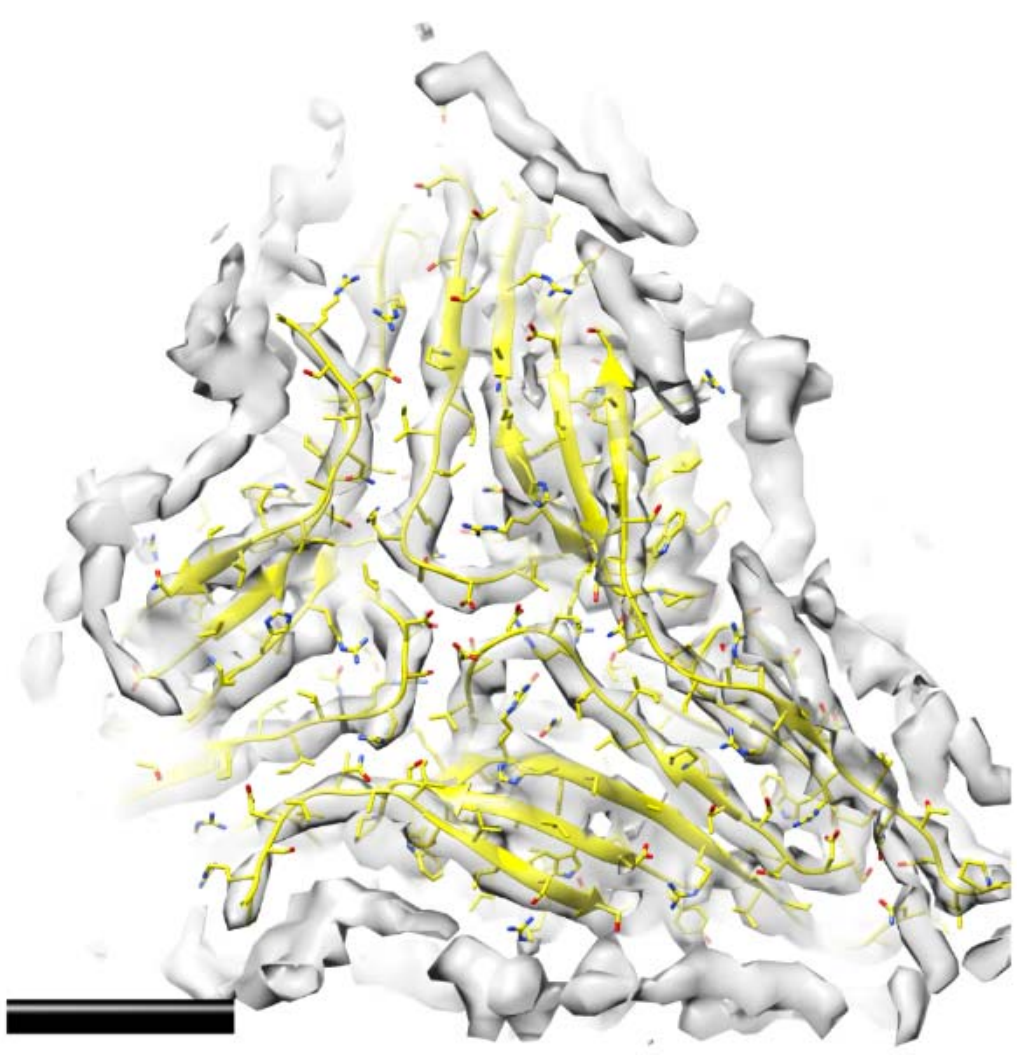

Figure S18 Nodavirus (EMPIAR-10203) downsampled before PASR processing. This dataset permits comparison of behaviour with respect to PASR processing for single frame micrographs which have already undergone motion correction, however it is not recommended. (a) RELION gold standard FSC (corrected; black line, masked; blue line, unmasked; green line, phase randomised; red line) curve demonstrating the downsampled Nyquist limit, and (b) binned reconstruction, coloured by local resolution showing almost the entire map is at the Nyquist limit, scale bar 5 nm. Density displayed at 3σ. (c) Focussed view of the internal surface of the capsid, scale bar 2 nm. Density displayed at 6σ.

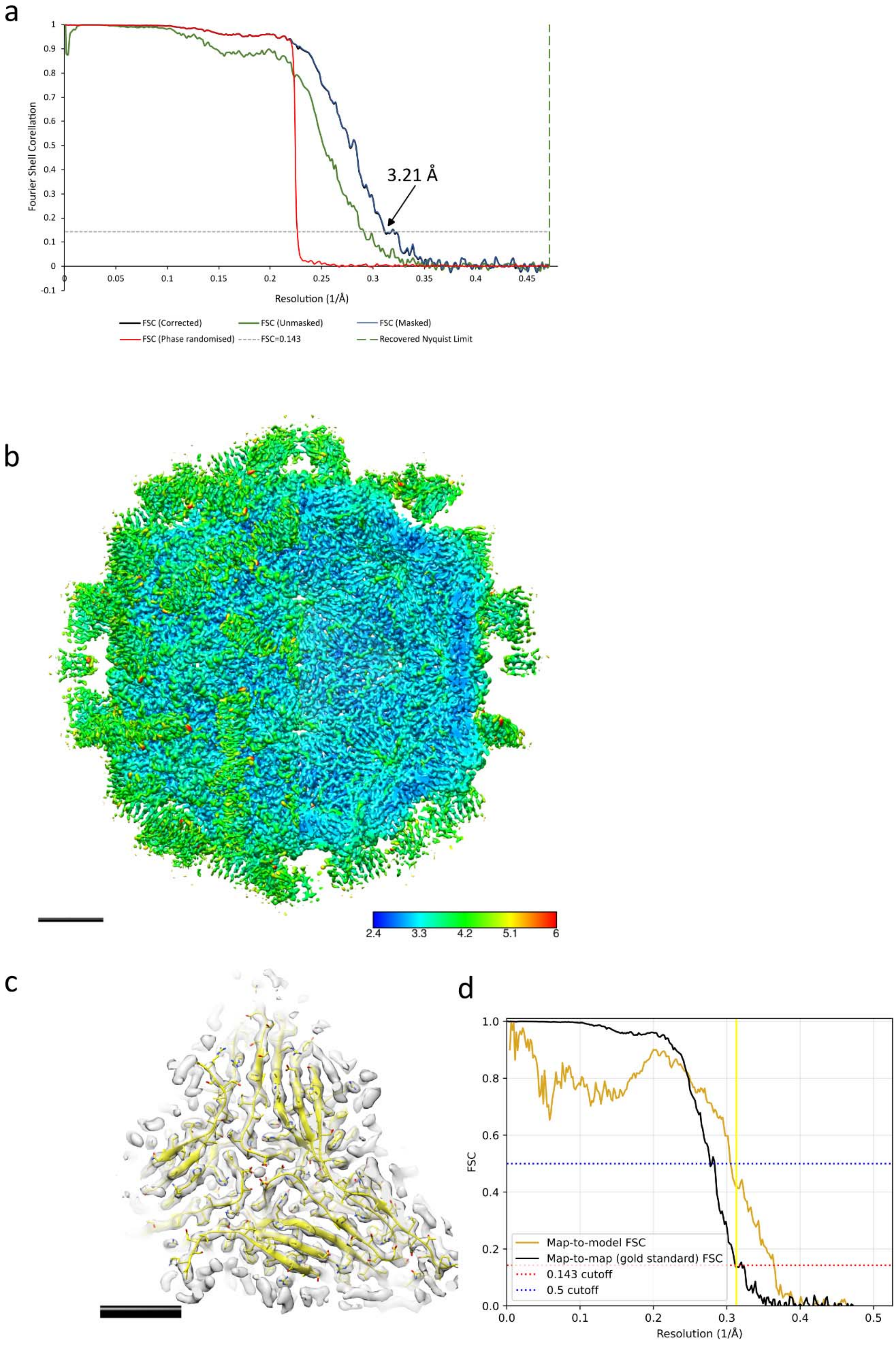

Figure S19 Nodavirus (EMPIAR-10203) after downsampled then upscaled with PASR processing. This dataset permits comparison of behaviour with respect to PASR processing for single frame

micrographs which have already undergone motion correction, however it is not recommended. (a) RELION gold standard FSC (corrected; black line, masked; blue line, unmasked; green line, phase randomised; red line) curve, reaching 3.21 Å, and (b) PASR reconstruction, coloured by local resolution which extends to ~2.4 Å, scale bar 5 nm. Density displayed at 3σ. (c) Focussed view of the internal surface of the capsid, scale bar 2 nm, (d) map-to-model FSC. Vertical yellow line indicates map-to-map gold-standard FSC resolution (Table S6). Density displayed at 6σ.

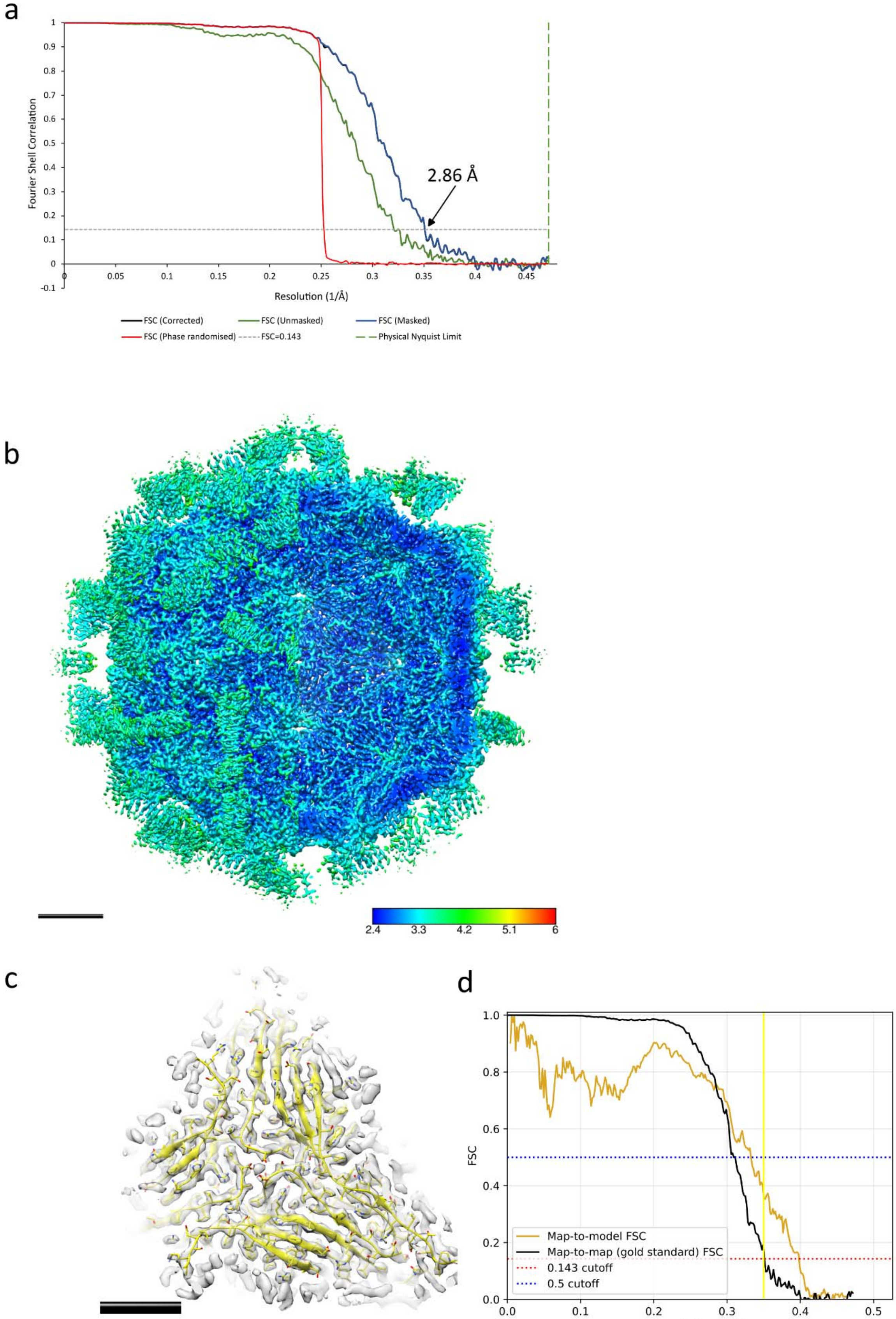

Figure S20 Nodavirus (EMPIAR-10203) original data reprocessed for comparison with a modern cryo-EM processing suite. This dataset permits comparison of behaviour with respect to PASR

processing for single frame micrographs which have already undergone motion correction, however it is not recommended. (a) RELION gold standard FSC (corrected; black line, masked; blue line, unmasked; green line, phase randomised; red line) curve, reaching 2.86 Å, and (b) Reconstruction of original data, coloured by local resolution with extends to ~2.4 Å, scale bar 5 nm. Density displayed at 3σ. (c) Scale bar 2 nm, (d) map-to-model FSC. Vertical yellow line indicates map-to-map gold-standard FSC resolution (Table S6). Density displayed at 6σ.

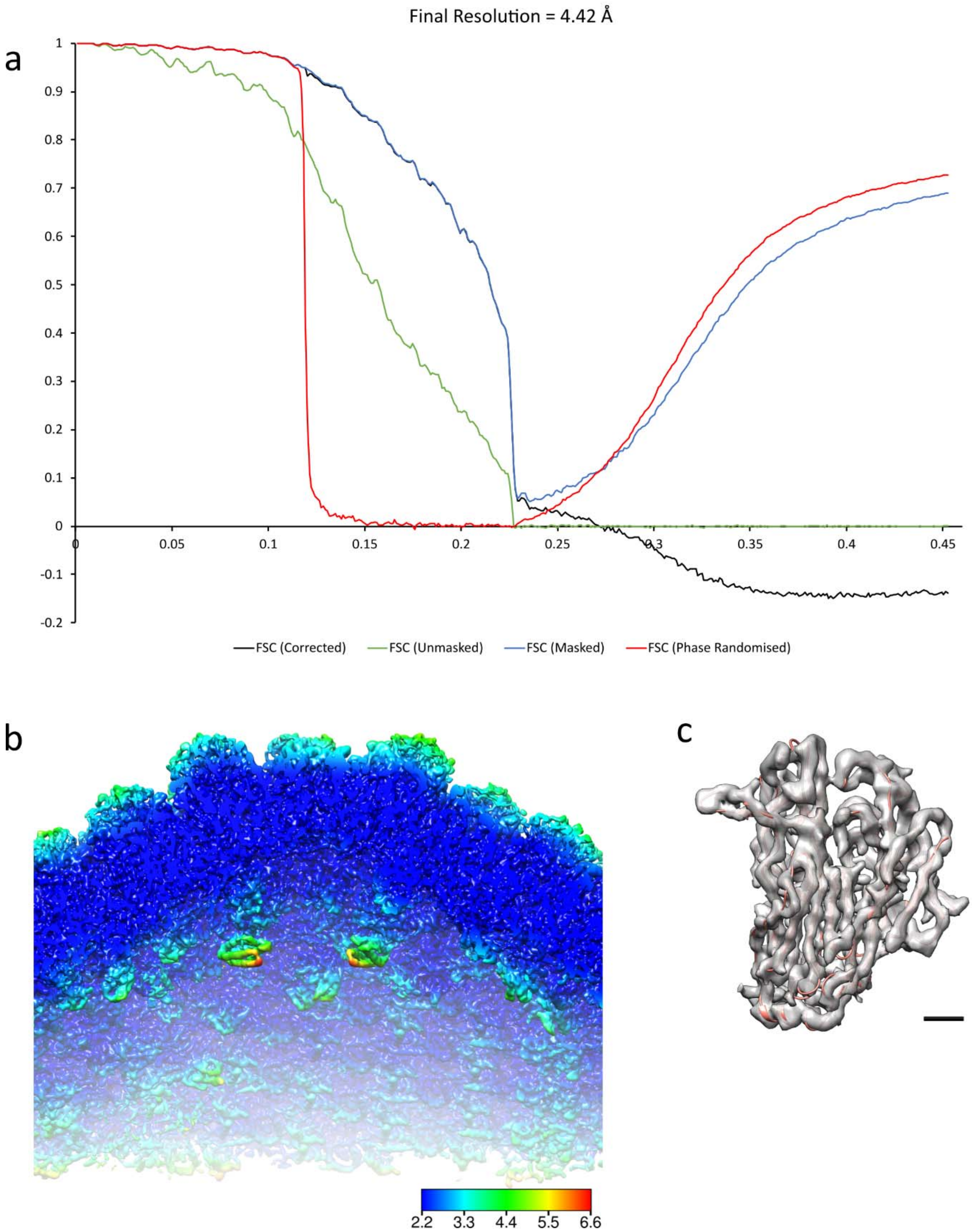

Figure S21 The effect of zero padding on the half maps of original data, demonstrating that PASR is acting as if acquired with super resolution. (a) RELION gold standard FSC (corrected; black line, masked; blue line, unmasked; green line, phase randomised; red line) showing immediately that zero padding is harmful; while the unmasked half map FSC acts as expected, the mask, corrected and phase randomised FSCs display behaviour which should immediately alert the user that something is awry, and (b) sliced view, coloured by estimated local resolution from the zero padded half maps, estimating resolution extending to the zero padded sampling limit. Map features do not support this resolution estimate and appear to be the same as the original map of 4.4 Å. (c) Extracted major capsid protein monomer showing complete lack of features one would expect from a 2.2 Å map. Scale bar equals 1 nm.

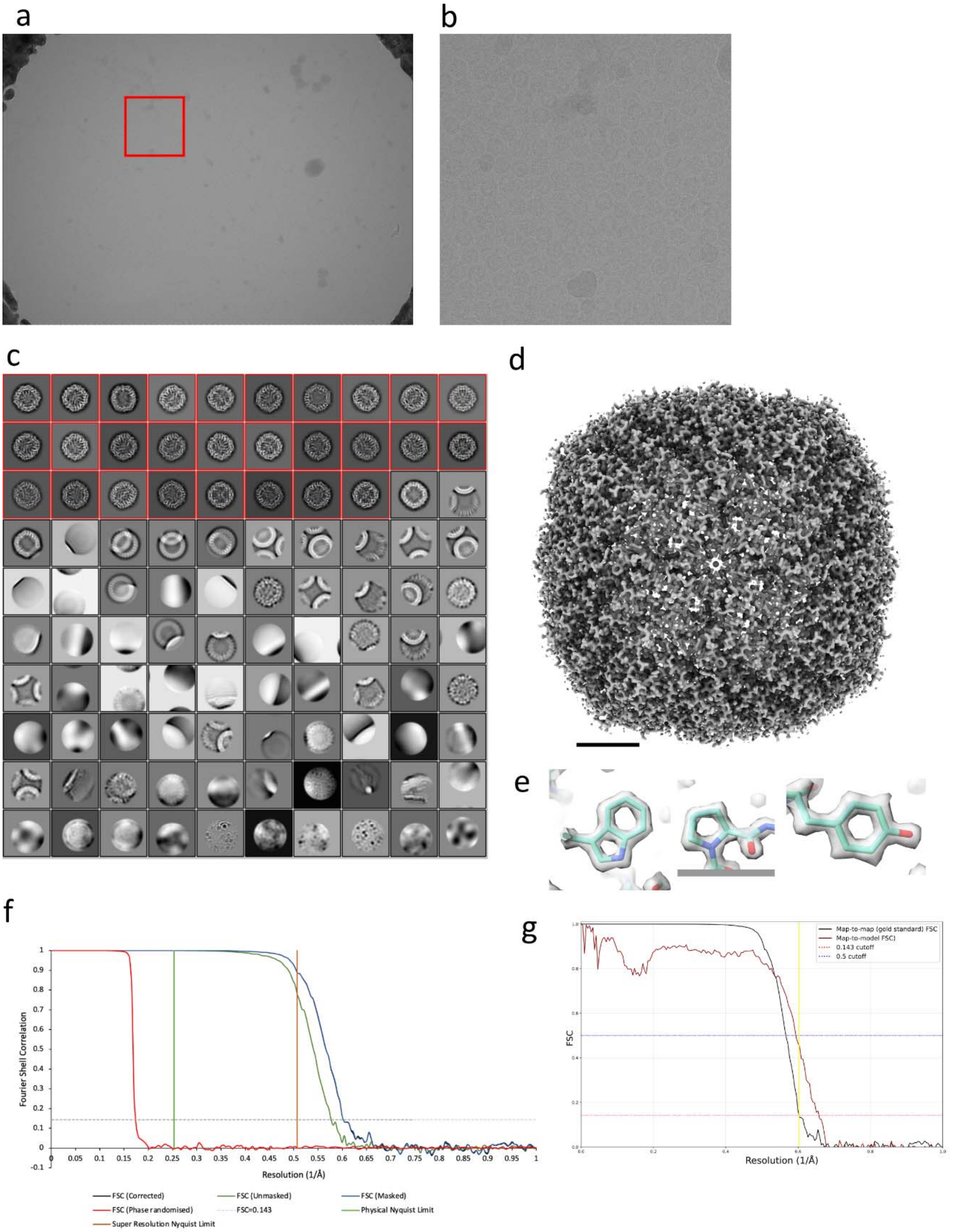

Figure S22 The application of PASR to Acquisition-time Super Resolution data. (a) Representative micrograph, (b) expanded view of red boxed area in (a), (c) 2D classes, selected classes boxed in red, (d) final apoferritin reconstruction, (e) FSC curve, showing the final map has far exceeded both the physical and super resolution Nyquist limits, (f) representative side chains: tryptophan, proline and tyrosine, (g) map-to-model FSC. Vertical yellow line indicates map-to-map gold-standard FSC resolution (Table S8).

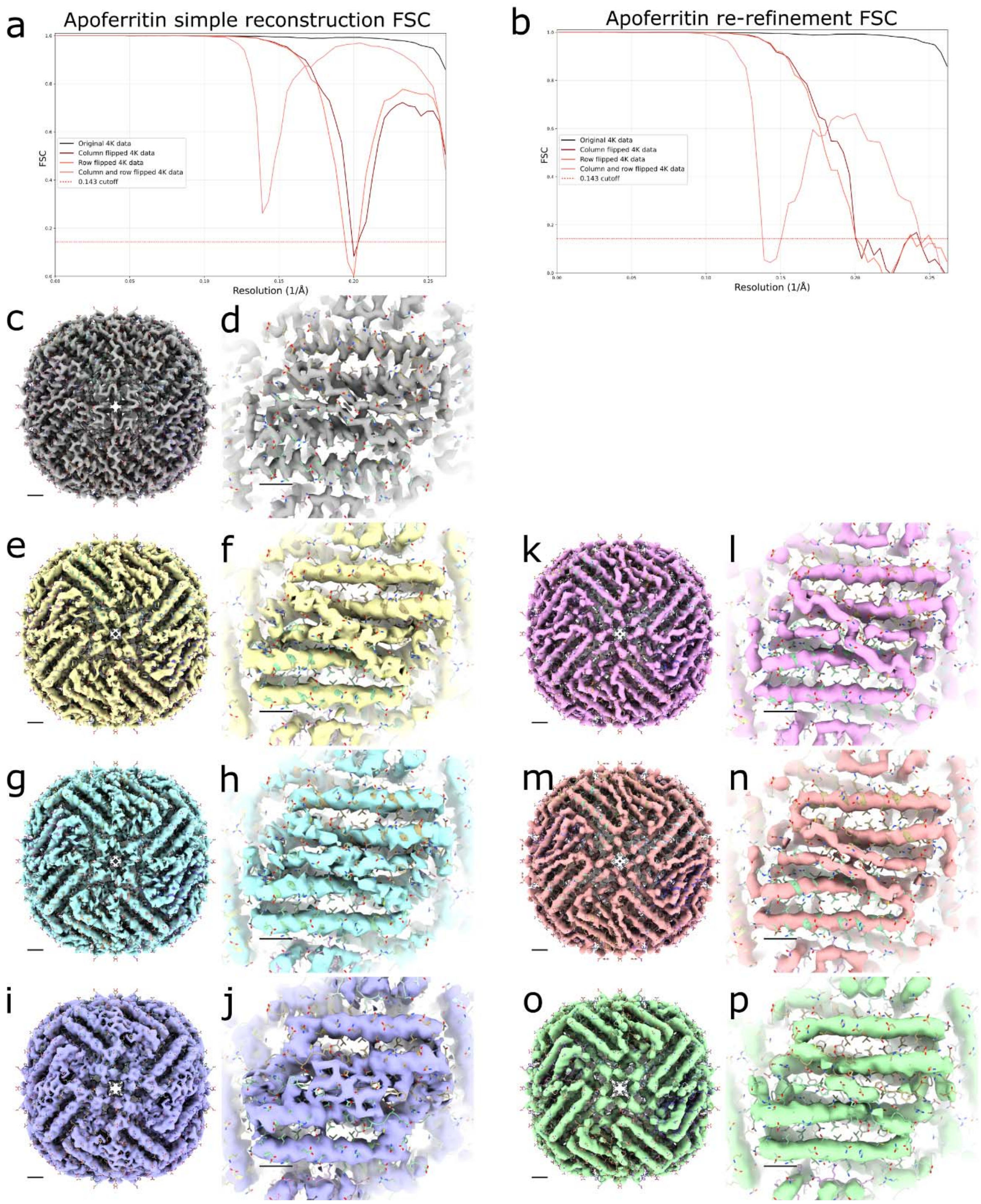

Figure S23 Pixel flipping tests for apoferritin at 1.912 Å/pixel, 4K counting mode data. (a) Gold standard FSC curves for simple reconstructions of original data (black line), column-flipped data (dark red line), row-flipped data (red line) and column-and-row flipped data (coral line). (b) Gold standard FSC curves for re-refinements of original data (black line), column-flipped data (dark red line), row-flipped data (red line) and column-and-row-flipped data (coral line). (c) whole view of reconstructed original apoferritin, (d) focussed view of reconstructed original apoferritin. While clearly limited by sampling rate, the reconstruction shows clear features for side chains commensurate

with reported resolution and strong backbone density. (e) whole view of reconstructed column-flipped apoferritin, (f) focussed view of column-flipped apoferritin, (g) whole view of reconstructed row-flipped apoferritin, (h) focussed view of row-flipped apoferritin, (i) whole view of reconstructed column-and-row-flipped apoferritin, (j) focussed view of column-and-row-flipped apoferritin. The with simple reconstructions (where angles have already been assigned optimally for original data) the pixel flipped maps become nearly unrecognisable. (k) whole view of re-refined column-flipped apoferritin, (l) focussed view of re-refined column-flipped apoferritin, (m) whole view of re-refined row-flipped apoferritin, (n) focussed view of row-flipped apoferritin, (o) whole view of re-refined column-and-row-flipped apoferritin, (p) focussed view of re-refined column-and-row flipped apoferritin. Re-refining angular assignments for individually column- or row-flipped apoferritin recovers some of the pathology of the FSC at the cost of final map resolution, however the column-and-row flipped data is unrecoverable. See Table S9 for FSC = 0.143, B-factor and Q-scores.

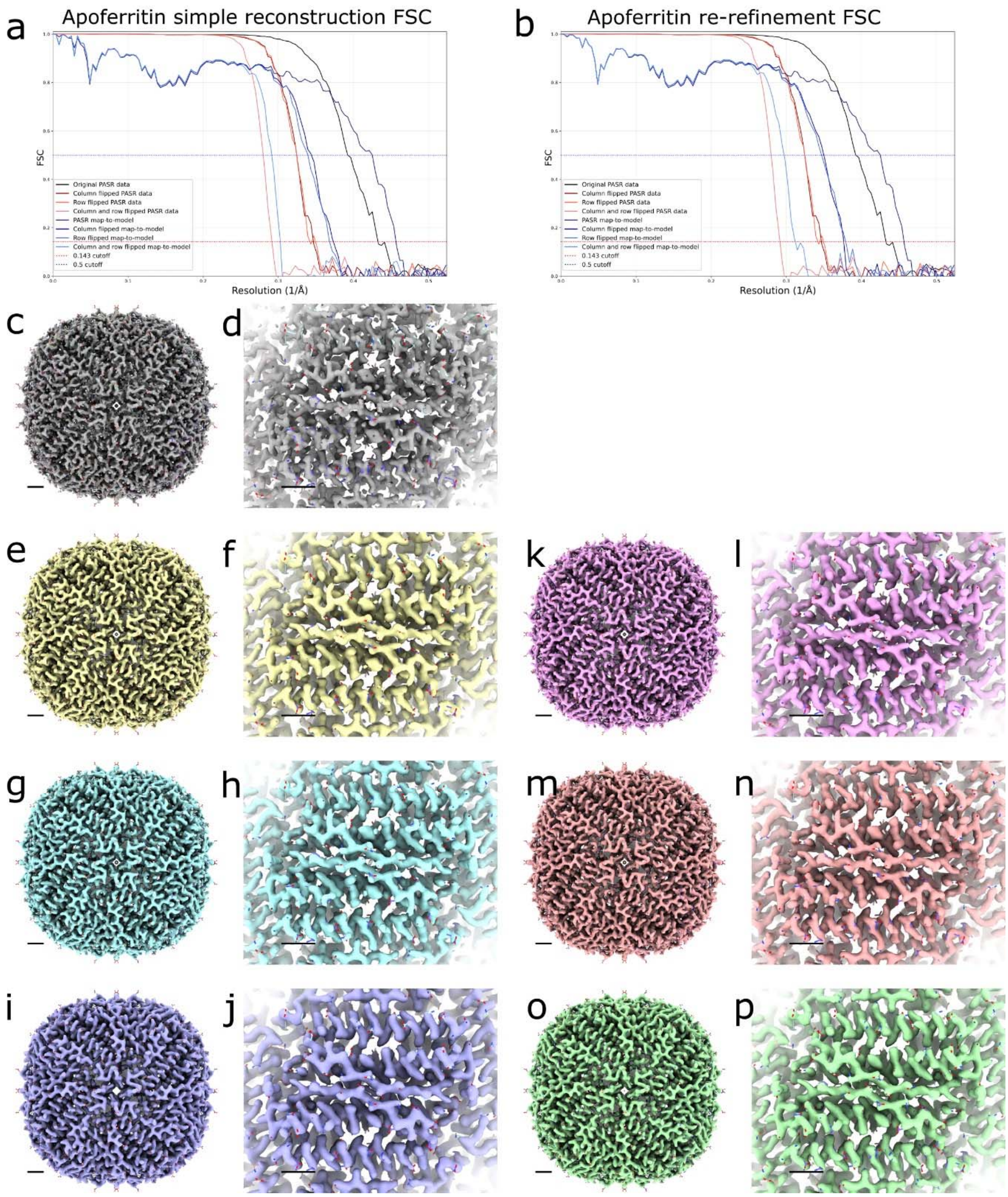

Figure S24 Pixel flipping tests for apoferritin at 0.956 Å/pixel and PASR processed. (a) Gold standard FSC curves for simple reconstructions of original data (black line), column-flipped data (dark red line), row-flipped data (red line) and column-and-row flipped data (coral line). Map-to-model FSC curves for simple reconstructions of the 8K PASR data (dark blue), column-flipped (royal blue), row-flipped (blue) and column-and-row-flipped (cornflower blue). (b) Gold standard FSC curves for re-refinements of original data (black line), column-flipped data (dark red line), row-flipped data (red line) and column-and-row-flipped data (coral line). Map-to-model FSC curves for simple reconstructions of the 8K PASR data (dark blue), column-flipped (royal blue), row-flipped

(blue) and column-and-row-flipped (cornflower blue). (c) whole view of reconstructed original apoferritin, (d) focussed view of reconstructed original apoferritin. The original reconstruction is reaching resolutions where side chains are easy to distinguish, and small molecules may be reliably assigned to density. (e) whole view of reconstructed column-flipped apoferritin, (f) focussed view of column-flipped apoferritin, (g) whole view of reconstructed row-flipped apoferritin, (h) focussed view of row-flipped apoferritin, (i) whole view of reconstructed column-and-row-flipped apoferritin, (j) focussed view of column-and-row-flipped apoferritin. With simple reconstructions (where angles have already been assigned optimally for original data) the pixel flipped maps lose some resolution and with column-and-row-flipping the FSC displays some pathology (an extreme drop). (k) whole view of re-refined column-flipped apoferritin, (l) focussed view of re-refined column-flipped apoferritin, (m) whole view of re-refined row-flipped apoferritin, (n) focussed view of row-flipped apoferritin, (o) whole view of re-refined column-and-row-flipped apoferritin, (p) focussed view of re-refined column-and-row flipped apoferritin. Re-refining angular assignments for individually column- or row-flipped apoferritin does nothing to recover the loss of resolution. Map-to-model FSCs demonstrate a degree of overfitting. B-factors are impacted, increasing 50% (for individually column or row flipped data) and 100% for column-and-row flipped data. See Table S9 for FSC = 0.143, FSC= 0.5, B-factor and Q-scores.

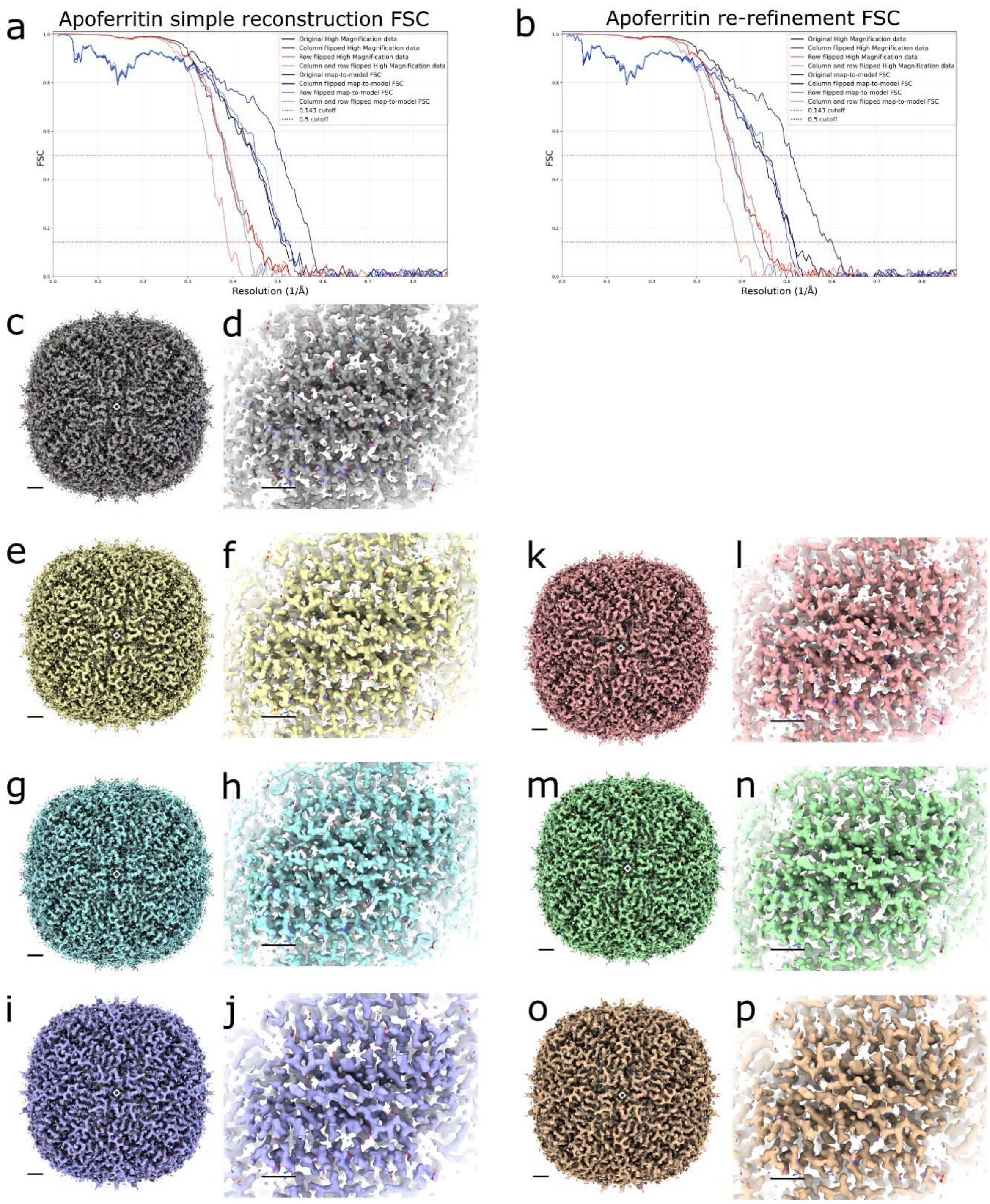

Figure S25 Pixel flipping tests for apoferritin at 0.57 Å/pixel in 4K counting mode. (a) Gold standard FSC curves for simple reconstructions of original data (black line), column-flipped data (dark red line), row-flipped data (red line) and column-and-row flipped data (coral line). Map-to-model FSC curves for simple reconstructions of the 4K high magnification data (dark blue), column-flipped (royal blue), row-flipped (blue) and column-and-row-flipped (cornflower blue). (b) Gold standard FSC curves for re-refinements of original data (black line), column-flipped data (dark red line), row-flipped data (red line) and column-and-row-flipped data (coral line). Map-to-model FSC curves for

simple reconstructions of the 4K high magnification data (dark blue), column-flipped (royal blue), row-flipped (blue) and column-and-row-flipped (cornflower blue). (c) whole view of reconstructed original apoferritin, (d) focussed view of reconstructed original apoferritin. Despite being a small dataset of 30 micrographs (~4,000 particles), the original reconstruction is reaching resolutions where side chains are easy to distinguish, and small molecules may be reliably assigned to density. (e) whole view of reconstructed column-flipped apoferritin, (f) focussed view of column-flipped apoferritin, (g) whole view of reconstructed row-flipped apoferritin, (h) focussed view of row-flipped apoferritin, (i) whole view of reconstructed column-and-row-flipped apoferritin, (j) focussed view of column-and-row-flipped apoferritin. With simple reconstructions (where angles have already been assigned optimally for original data) the pixel flipped maps lose some resolution and with column-and-row-flipping further losing resolution. Some densities, which could be attributable to small molecules, water or noise deviate from their original locations. (k) whole view of re-refined column-flipped apoferritin, (l) focussed view of re-refined column-flipped apoferritin, (m) whole view of re-refined row-flipped apoferritin, (n) focussed view of row-flipped apoferritin, (o) whole view of re-refined column-and-row-flipped apoferritin, (p) focussed view of re-refined column-and-row flipped apoferritin. Re-refining angular assignments for individually column- or row-flipped apoferritin does nothing to recover the loss of resolution but does not do further harm. Map-to-model FSCs demonstrate no overfitting. B-factors are impacted, but less so than with the lower magnification data; increasing ~25% (for individually column or row flipped data) and ~75% for column-and-row flipped data. See Table S9 for FSC = 0.143, FSC= 0.5, B-factor and Q-scores.

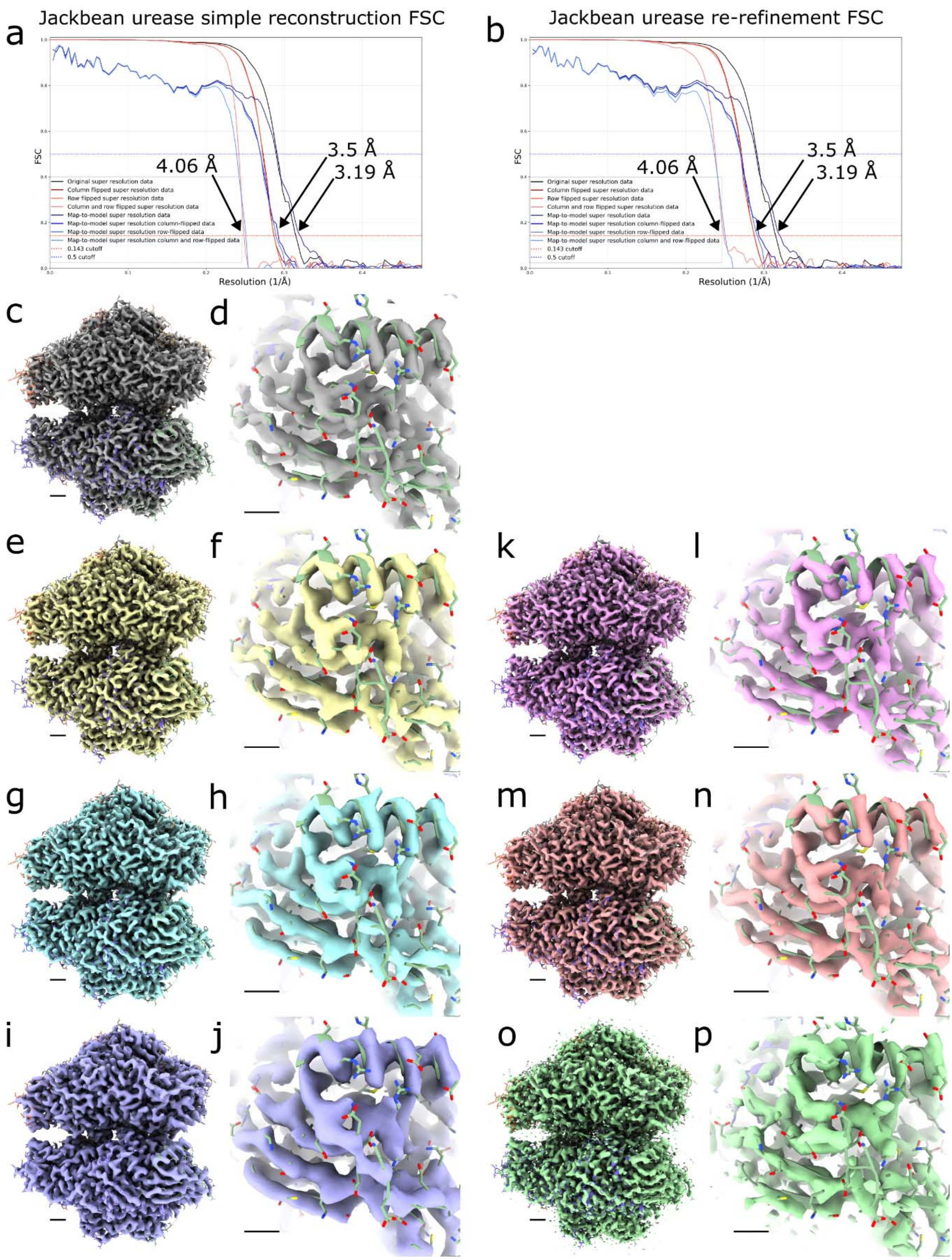

Figure S26 Pixel flipping tests for jackbean urease (EMPIAR 10549) super resolution data prior to Bayesian polishing. (a) Gold standard FSC curves for simple reconstructions of original data (black line), column-flipped data (dark red line), row-flipped data (red line) and column-and-row flipped data (coral line). Map-to-model FSC curves for simple reconstructions of the super resolution data

(dark blue), column-flipped (royal blue), row-flipped (blue) and column-and-row-flipped (cornflower blue). (b) Gold standard FSC curves for re-refinements of original data (black line), column-flipped data (dark red line), row-flipped data (red line) and column-and-row-flipped data (coral line). Map-to-model FSC curves for simple reconstructions of the super resolution data (dark blue), column-flipped (royal blue), row-flipped (blue) and column-and-row-flipped (cornflower blue). (c) whole view of reconstructed original super resolution jackbean urease, (d) focussed view of reconstructed original super resolution jackbean urease. The original reconstruction is reaching resolutions where side chains can be distinguished distinguish. (e) whole view of reconstructed column-flipped jackbean urease, (f) focussed view of column-flipped jackbean urease, (g) whole view of reconstructed row-flipped jackbean urease, (h) focussed view of row-flipped jackbean urease, (i) whole view of reconstructed column-and-row-flipped jeackbean urease, (j) focussed view of column-and-row-flipped jeackbean urease. With simple reconstructions (where angles have already been assigned optimally for original data) the pixel flipped maps lose some resolution and with column-and-row-flipping further losing resolution. (k) whole view of re-refined column-flipped jackbean urease, (l) focussed view of re-refined column-flipped jackbean urease, (m) whole view of re-refined row-flipped jackbean urease, (n) focussed view of row-flipped jackbean urease, (o) whole view of re-refined column-and-row-flipped jackbean urease, (p) focussed view of re-refined column-and-row flipped apoferritin. Re-refining angular assignments for individually column- or row-flipped apoferritin does nothing to recover the loss of resolution but does not do further harm. For both simple reconstruction and re-refinement, combined column-and-row flipping creates some main chain breaks (j, p). Map-to-model FSCs demonstrate underfitting. B-factors are impacted; increasing ~10-20% (for individually column or row flipped data for simple reconstruction or re-refinement respectively) and ~25-100% for column-and-row flipped data (simple reconstruction or re-refinement respectively). See Table S9 for FSC = 0.143, FSC= 0.5, B-factor and Q-scores.

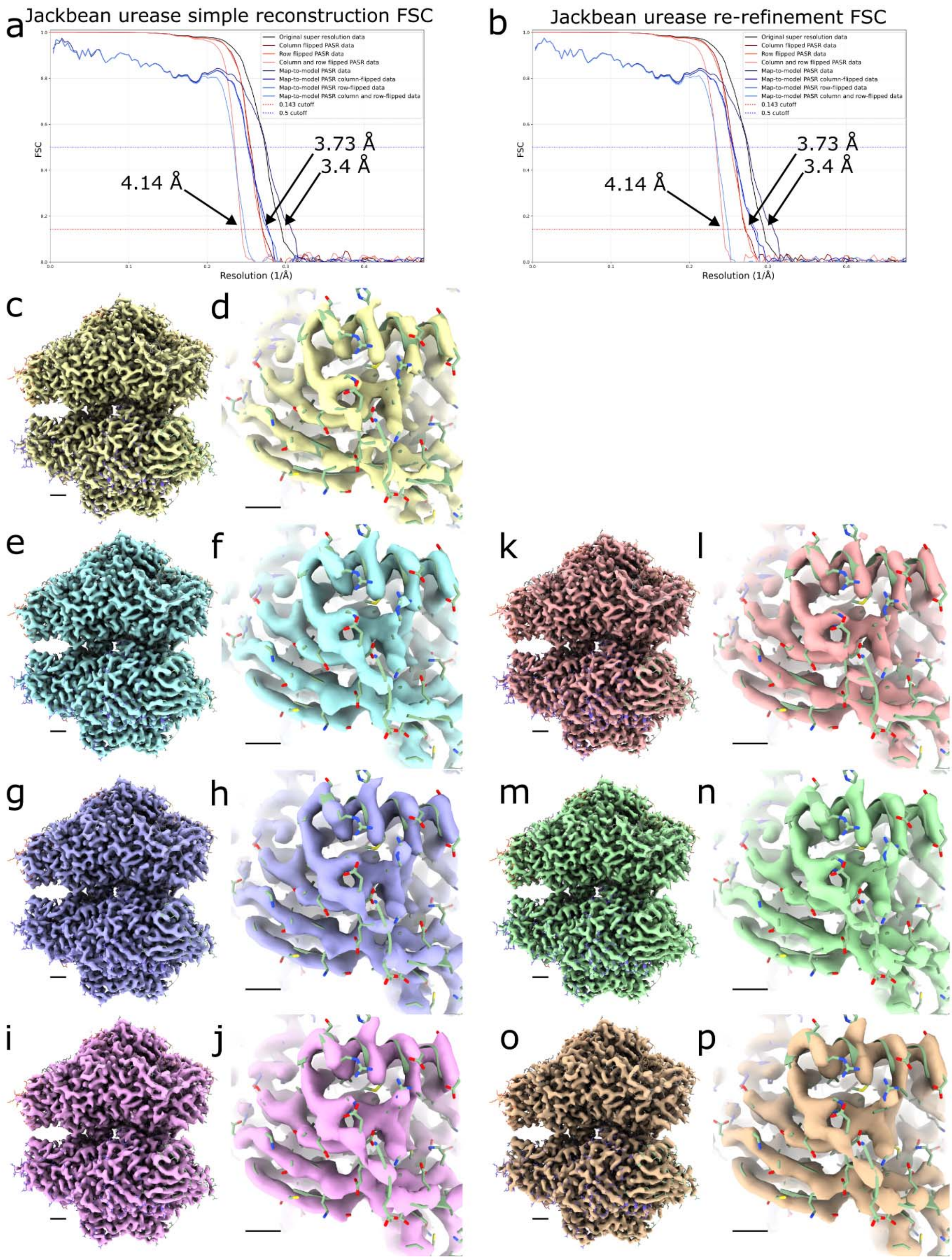

Figure S27 Pixel flipping tests for jackbean urease (EMPIAR 10549) PASR data prior to Bayesian polishing. (a) Gold standard FSC curves for simple reconstructions of original data (black line), column-flipped data (dark red line), row-flipped data (red line) and column-and-row flipped data (coral line). Map-to-model FSC curves for simple reconstructions of the PASR data (dark blue),

column-flipped (royal blue), row-flipped (blue) and column-and-row-flipped (cornflower blue). (b) Gold standard FSC curves for re-refinements of original data (black line), column-flipped data (dark red line), row-flipped data (red line) and column-and-row-flipped data (coral line). Map-to-model FSC curves for simple reconstructions of the super resolution data (dark blue), column-flipped (royal blue), row-flipped (blue) and column-and-row-flipped (cornflower blue). (c) whole view of reconstructed original super resolution jackbean urease, (d) focussed view of reconstructed original super resolution jackbean urease. The original reconstruction is reaching resolutions where side chains can be distinguished distinguish. (e) whole view of reconstructed column-flipped jackbean urease, (f) focussed view of column-flipped jackbean urease, (g) whole view of reconstructed row-flipped jackbean urease, (h) focussed view of row-flipped jackbean urease, (i) whole view of reconstructed column-and-row-flipped jeackbean urease, (j) focussed view of column-and-row-flipped jeackbean urease. With simple reconstructions (where angles have already been assigned optimally for original data) the pixel flipped maps lose some resolution and with column-and-row-flipping further losing resolution. (k) whole view of re-refined column-flipped jackbean urease, (l) focussed view of re-refined column-flipped jackbean urease, (m) whole view of re-refined row-flipped jackbean urease, (n) focussed view of row-flipped jackbean urease, (o) whole view of re-refined column-and-row-flipped jackbean urease, (p) focussed view of re-refined column-and-row flipped apoferritin. Re-refining angular assignments for individually column- or row-flipped apoferritin does nothing to recover the loss of resolution but does not do further harm. For both simple reconstruction and re-refinement, combined column-and-row flipping creates some main chain breaks (j, p). Map-to-model FSCs demonstrate underfitting, similarly to the super resolution data. B-factors are impacted, although slightly less than for super resolution data; increasing ~5-14% (for individually column or row flipped data for simple reconstruction or re-refinement respectively) and ~40% for column-and-row flipped data (simple reconstruction or re-refinement respectively). See Table S9 for FSC = 0.143, FSC= 0.5, B-factor and Q-scores.
